# Genetic structure of the commercial stingless bee *Heterotrigona itama* (Apidae: Meliponini) in Thailand

**DOI:** 10.1371/journal.pone.0312386

**Published:** 2024-12-04

**Authors:** Kanyanat Wongsa, Ekgachai Jeratthitikul, Pisit Poolprasert, Orawan Duangphakdee, Atsalek Rattanawannee

**Affiliations:** 1 Department of Entomology, Faculty of Agriculture, Kasetsart University, Chatuchak, Bangkok, Thailand; 2 Animal Systematic and Molecular Ecology Laboratory, Department of Biology, Faculty of Science, Mahidol University, Bangkok, Thailand; 3 Native Honeybee and Pollinator Research Center, King Mongkut’s University of Technology Thonburi, Bangkok, Thailand; 4 Research and Lifelong Learning Center for Urban and Environmental Entomology, Kasetsart University Institute for Advanced Studies, Kasetsart University, Bangkok, Thailand; University of Minnesota, UNITED STATES OF AMERICA

## Abstract

Stingless beekeeping, also known as meliponiculture, has gained increasing popularity in many tropical and subtropical countries for its use in commercial pollination and high-value honey and propolis production. However, this rising interest in stingless beekeeping has led to significant geographical displacements of bee colonies by beekeepers, occasionally surpassing their native ranges. Consequently, this affects local bee populations by disrupting gene flow across unnaturally large geographic scales. For *Heterotrigona itama*, one of the most common stingless bee species in Southeast Asian countries, including Thailand, there is concern that large-scale artificial propagation by beekeepers utilizing a limited number of bee colonies will lead to inbreeding. This practice leads to increased inbreeding within managed populations and introgression into wild populations. These concerns highlight the need for careful management practices in stingless beekeeping to mitigate potential adverse effects. To assess the genetic structure of *H*. *itama* in Thailand, 70 colonies were sampled, and partially sequenced cytochrome c oxidase subunit 1 (*COI*) gene, large ribosomal subunit rRNA gene (*16S rRNA*), and 28S large ribosomal subunit rDNA gene (*28S rRNA*) were analyzed. Our results showed slightly lower nuclear genetic variability, but higher mitochondrial genetic variability, which can be attributed to gene flow, colony transport, and nest division. We suggest that increasing the number of colonies maintained through nest division does not negatively affect genetic variability, as it is maintained by small-scale male dispersal and human-mediated nest transport. However, caution should be exercised when transporting nests from distant localities, considering the high genetic differentiation observed between samples from Narathiwat and those from Krabi and Nakhon Si Thammarat provinces, which might indicate local adaptation.

## 1. Introduction

Stingless bees are eusocial insects that are widely distributed across subtropical and tropical regions [1]. These eusocial bees, with more than 500 valid species [1], are diverse in terms of their external morphology, body size, colony size, and foraging strategies [2, 3]. Moreover, stingless bees serve as major pollinators for numerous native and cultivated plant species across tropical and subtropical regions [4–6]. Their advantages as pollinators include floral constancy, maintenance of perennial populations and colonies, non-functional stings, ease of handling, and marked worker recruitment behavior [2, 7].

In Thailand, at least 33 species of stingless bees belonging to 10 genera have been reported; however, many of these species are poorly documented [8, 9]. Over the past two decades, stingless beekeeping, also known as meliponiculture, for both pleasure and profit has become popular among professional and amateur beekeepers alike in Thailand. Currently, at least six species, including *Tetragonula pagdeni*, *T*. *laeviceps*, *T*. *fuscobalteata*, *Lepidotrigona terminata*, *Geniotrigona thoracica*, and *Heterotrigona itama*, are successfully managed in standard wooden hive boxes for commercial pollination services in greenhouses and open fields for honey and propolis productions, and the commercial sale of colonies [9, 10]. Among these, *H*. *itama* is one of the most important stingless bee species with a limited distribution in southern Thailand, Malaysia, Singapore, and Indonesia [8]. *Heterotrigona itama* is commonly sued in meliponiculture in Thailand, where it is propagated in apiaries for the purpose of selling colonies, producing honey products, and pollinating crops [9]. Notably, the selling price of *H*. *itama* honey is approximately 1200–1500 Thai Baht (32–40 USD) per kilogram, which is ten times higher than that of honey produced by Thai *Apis mellifera* [9]. In addition, colonies of *H*. *itama* are typically priced between 3,000 and 5,000 Thai Baht (80–134 USD) per colony (personal communication). With the growing demand for stingless bee honey, meliponiculture has garnered increasing interest. Consequently, *H*. *itama* meliponiculture has the substantial potential to augment household income in numerous rural communities across Thailand.

In 2014, stingless bee hive products were estimated to have contributed 5.76 million Thai Baht (about 177,500 USD) to the regional economy [9]. With the burgeoning interest in stingless beekeeping, the number of new stingless beekeepers in Thailand has been steadily rising to meet the demands of both domestic and international markets. Consequently, stingless beekeepers are expanding their apiaries by acquiring colonies not only from other commercial apiaries, but also from natural colonies.

Trade in stingless bee colonies enable their geographic displacement both within and outside their natural range [11, 12]. There is ample evidence to suggest that the introduction of non-native bee species into new ecosystems can disrupt native biodiversity [13–16] and ecosystem processes [17, 18]. Additionally, the anthropogenic-associated geographic displacement of stingless bee colonies can also contribute to the spread of pests and diseases [12, 16, 19–21]. Therefore, evaluating the genetic structure and variation of stingless bee populations is essential for their effective management and long-term success in domestication [21, 22]. This data can also help stingless beekeepers avoid meliponicultural practices that may lead to inbreeding and the loss of genetic diversity [12]. While some research has begun exploring the genome data [23] and transcriptome profile [24] of the Malaysian stingless bee, *H*. *itama*, this field remains in its infancy.

Hybridization and mating interference are recognized as the genetic consequences of displacing bees across their natural hybrid zones and geographic barriers [16]. Hybridization refers to the genetic mixing of two previously isolated populations [16], which can result in genetic homogenization and the loss of unique ecotypes present in the original population [25]. Therefore, the loss of allele combinations unique to an indigenous population can lead to the genomic extinction of locally-adapted genotypes [16, 26]. Mating interference refers to sexual interactions between individuals of different species that cause a decline in reproductive success [27]. Thus, mating interference can result in the reduced fitness of indigenous bee populations due to reduced fertility [12, 16, 28–30].

The levels of inbreeding and population subdivision in captive and wild stingless bee populations vary across studies [31–33], indicating that results from one species and location cannot be extrapolated to another species [12]. However, the effects of management practices appear to be more significant to the genetic structure of a bee population than species dispersal [34, 35], habitat fragmentation, deforestation, altitude and climate [11]. In this study, we determined the genetic structure of managed and wild populations of the stingless bee *H*. *itama*, a species commonly maintained for honey production and as a pollination vector in commercial crops in Thailand. Moreover, we examined whether managed and wild *H*. *itama* populations were genetically distinct from each other. Additionally, we investigated whether the managed *H*. *itama* population in Thailand was more inbred than wild populations. The results provide valuable insights into the influence of meliponicultural practices as well as the fundamental biology of this stingless bee species.

## 2. Materials and methods

### 2.1 Sample collection and identification

**Fig 1:**
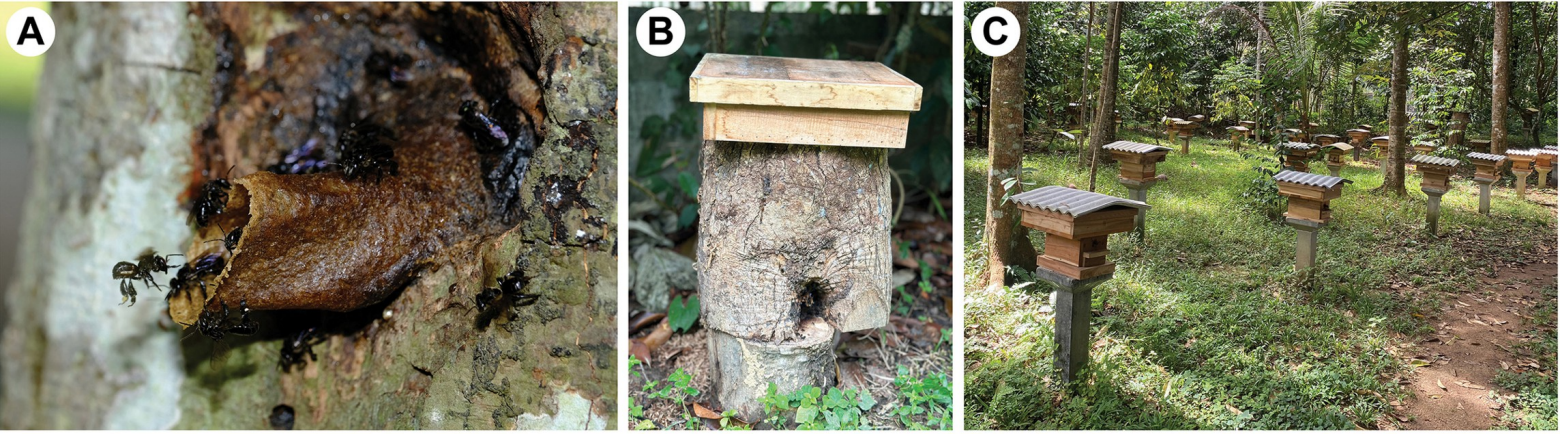
The colonies of *Heterotrigona itama* were categorized as either wild and managed primarily based on the origins of the stingless bee colonies. Wild colonies were characterized by colonies as those with original, undivided nests located either in trees/natural cavities (A), or in the original tree, which had been cut and moved to a nearby vicinity, often with a honey super placed on top (B). On the other hand, managed colonies were identified by a divided nest structure and ongoing maintenance (including the trade of honey) (C). Photo by A. Rattanawannee.

Between 2022 and 2023, we sampled 39 managed colonies of *H*. *itama* belonging to six beekeepers from eight commercial meliponaries (S1 Table). Additionally, we surveyed and collected 31 wild colonies from across the distribution range of *H*. *itama* in southern Thailand (S1 Table) [8, 9]. Then, the colony samples were categorized as wild or managed, primarily based on the origins of the stingless bee colonies. The wild colonies (indigenous wild) were characterized by original undivided nests those either in trees/natural cavities (Fig 1A) or in the original tree, which had been cut and moved to a nearby vicinity, often with a honey super placed on top (Fig 1B). In contrast, managed colonies were identified by divided nest structures and ongoing maintenance within artificial hives (Fig 1C), including those used in the honey trade. The origin of all bee colonies was confirmed through personal communication with beekeepers who own the nests. GPS coordinates were recorded for each collection locality. At least five adult worker bees were collected directly from the nest-entrance tubes of each colony, and separated into two sets. In the first set, two bee specimens were preserved in 70% (v/v) ethanol and used in the species confirmation through morphological examination. In the second set, at least three specimens were immediately preserved in 95% (v/v) ethanol, stored in −20°C, and used for genetic analyses. Morphological identification was made based on previous taxonomic literature of the Indo-Malayan/Australasian stingless bees, following the guidelines provided by Rasmussen, 2008 [8], Schwarz, 1939 [36], Sakagami *et al*., 1985 [37], Samsudin *et al*., 2018 [38], Trianto *et al*., 2023 [39], and Siti-Fatimah *et al*., 2018 [40].

### 2.2 Ethics statement

Stingless beekeepers welcomed the study of this commercial stingless bee species. No specific permits were obtained as the field study did not include endangered or protected species. The number of samples collected was minimal, and ethical treatment was properly applied according to standard research methods. All animal experiments conformed to the guidelines established by the Animal Experiment Committee of Kasetsart University, Thailand (Approval no. ACKU66−AGR−015).

### 2.3 DNA extraction, amplification, and sequencing

Whole genomic DNA was extracted from the thoracic muscle of one worker bee per colony using a DNeasy® Blood & Tissue kit (Qiagen, Germantown, MD, US) according to the manufacturer’s instructions. Fragments from the mitochondrial cytochrome c oxidase subunit I gene (*COI*), mitochondrial large ribosomal subunit rRNA gene (*16S rRNA*), and nuclear 28S large ribosomal subunit rDNA gene (*28S rRNA*) were amplified and sequenced using the primers LoboF1 and LoboR1 for *COI* [41], 16sar-L-myt and 16Sbr-H-myt for *16S rRNA* [42], and C1 and D2 for *28S rRNA* [43]. Primer names, references, and sequences are shown in Table 1. Polymerase chain reaction (PCR) was conducted using a T100™ thermal cycler (BIO-RAD) with a final reaction volume of 30 μL containing 1× Multiplex PCR Master Mix (Green HotStart PCR Master Mix, Biotechrabbit), 20 μmol/L of each primer, and at least 10 ng of the genomic DNA template, and distilled water up to 30 μL to reach the total volume. PCR amplification was performed with the following conditions: an initial denaturation step at 94°C for 10 min; 30 cycles of 60 s at 94°C, 30 s of annealing (case-by-case between 48°C and 55°C, depending on the primer pair) and 120 s at 72°C; and a final extension at 72°C for 10 min. The amplified PCR products were purified using a MEGA quick-spin TM plus (Fragment DNA purification kit) and sequenced in both directions using an automated sequencer (ABI prism 3730XL).

**Table 1:** Table of primer sequences.

Target gene	Primer sequence (5′ → 3′)	Annealing temperature	Product size	Reference
*COI*	LOBOF1: KBTCHACAAAYCAYAARGAYATHGG	46°C	660 bp	Lobo *et al*., (2013) [41]
	LOBOR1: TGRTTYTTYGGWCAYCCWGARGTTTA			
*16s*	LR13943: CACCTGTTTATCAAAAACAT	45°C	440 bp	Lydeard *et al*., (1996) [42]
	LR13392: CGTCGATTTGAACTCAAATC			
*28s*	28sD2: AAGAGAGAGTTCAAGAGTACGTG	55°C	605 bp	Jovelin and Justine (2001) [43]
	28sD3: TAGTTCACCATCTTTCGGGTCCC			

### 2.4 Sequence alignment and diversity indices analyses

Contigs of the forward and reverse sequences were edited and assembled using MEGA11 [44], and confirmed by visual inspection. The novel sequences obtained in this study were uploaded to the GenBank Nucleotide sequence database under the accession numbers listed in S1 Table. No insertions, deletions, or stop codons were observed in the *COI* gene. The sequences were aligned separately for each gene using the ClustalW algorithm in MEGA11 [44]. The final matrix sequences of *COI*, *16S rRNA* and *28S rRNA* after editing were 660, 440, and 605 bp, respectively.

The number of nucleotide substitutions and compositions of the partial *COI*, *16s rRNA* and *28s rRNA* gene sequences were calculated using MEGA11 [44]. Subsequently, genetic diversity indices were investigated as the number of polymorphic sites (*S*), average number of nucleotide differences (*k*), number of haplotypes (*No*), haplotype diversity (*hd*), and average number of pairwise differences (*Pi*) using the DNAsp v5.0 program [45].

### 2.5 Neutrality test

To investigate the historical demographics of the *H*. *itama* populations in Thailand, we conducted Tajima’s *D* [46] and Fu’s *Fs* [47] neutrality tests. These statistical analyses were computed using ARLEQUIN [48]. A positive and significant Tajima’s *D* value indicates population subdivision or contraction, whereas a significantly negative value suggests a recent population size expansion. Furthermore, a large negative Fu’s *Fs* value suggests an excess of rare alleles in the population, implying a recent increase in the population size. In addition, we calculated Ramos-Onsins and Rozas’s R2 [49] using DNAsp v5.0 program [45], and significance was assessed through 1,000 coalescent simulations.

### 2.6 Population genetic differentiation and gene flow analyses

To analyze the genetic structure of the population, the Kimura 2-parameter (K2P) method [50] was utilized to examine the genetic distances between populations (wild and managed) using ARLEQUIN. To determine the effects of stingless beekeeping activities (colony movements) on the genetic structure of *H*. *itama*, molecular variance (AMOVA) [51] were generated for all gene sequences using ARLEQUIN. The population pairwise genetic distance (*F*_*st*_) was determined and used in the AMOVA with 1,000 permutations as a significance test (α = 0.05). Additionally, *F*-statistics were calculated to assess the degree of genetic differentiation, testing statistical significance with 1,000 permutations (α = 0.05). To assess gene flow (*Nm*), we performed pairwise migration rate analysis between groups (wild and managed populations) based on the following equilibrium relationship: *Nm* = (1–*F*_*st*_)/4 *F*_*st*_ [52]. We also estimated the Jost’s *D*_*est*_ [53] value between pairs of collection sites with 1,000 permutations using GENALEX 6.5 [54]. The *D*_*est*_ value of the population was unaffected by heterozygosity [53]. To verify isolation by distance, Mental tests between geographical and genetic distances among populations were calculated with 1,000 permutations using GENALEX.

### 2.7 Phylogenetic and haplotype network reconstruction

Details of the taxon sampling used in the phylogenetic analysis are listed in S1 Table. The dataset included 70 workers of *H*. *itama* as ingroups. Additionally, six workers from *Heterotrigona bakeri*, one worker from *Heterotrigona erythrogastra*, and seven workers from other related stingless bee taxa were used as outgroups (S1 Table and S1 File). The final concatenated alignment was divided into five partitions (three codons each for *COI*, *16S rRNA* and *28S rRNA*). The best-fit substitution model for each partition was determined using PartitionFinder2 v.2.3.4 [55] with the corrected Akaike Information Criterion (AICc). The best-fit model was identified as GTR+I for the first and second codons of *COI*, GTR+G for the third codon of *COI*, and GTR+I+G for *16S rRNA* and *28S rRNA* genes. These models were applied to each gene for subsequent phylogenetic analysis.

The phylogenetic tree was constructed using maximum likelihood (ML) and Bayesian inference (BI) with the online CIPRES Science Gateway [56]. The ML tree was estimated using IQ-TREE 2.2.2.7 [57] with 10,000 replicates of ultrafast bootstrap analysis (UFBoot) to assess topology bootstrap support (BS) [58]. Bayesian inference (BI) analysis was performed using MrBayes 3.2.7 [59]. Four Monte Carlo Markov Chains of 10,000,000 generations were run, with sampling at every 1,000 generations. The effective sample size was > 200 for all the parameters. A clade in the obtained phylogenetic trees was considered well supported if the ultrafast BS was ≥95% and the Bayesian bipartition posterior probability (bpp) was ≥ 0.95 [58, 60].

The median-joining network [61] was employed in PopART [62] to construct the haplotype network of each *16s rRNA* and *28s rRNA* genes (S2–S4 Files). The median-joining method uses the maximum parsimony approach to identify the shortest phylogenetic trees [61].

## 3. Results

### 3.1 DNA sequence variation

After removing the primers used in the PCRs, we obtained effective sequences for the mitochondrial *COI* and *16s rRNA* genes consisting of 660 bp and 440 bp, respectively. Analysis of the average nucleotide composition of these two mitochondrial genes revealed a high A+T nucleotide bias (*COI* = 58.0%; *16s rRNA* = 72.9%), which is a typical characteristics of animal mitochondrial genomes [63–69]. Multi-alignment and pairwise sequence comparisons of the *COI* sequences demonstrated 41 parsimony-informative single-base substitutions, comprising 32 transitions (78.05%) and 9 transversions (21.95%). For the *16s rRNA* genes, only four parsimony-informative sites were detected, consisting of three transitions and one transversion. We identified 42 unique *COI* haplotypes and 10 unique *16s rRNA* genes. Among the managed colony samples, we identified 30 *COI* haplotypes and nine *16s rRNA*-haplotypes from a total of 39 samples. In contrast, we detected only 15 *COI* haplotypes and nine *16s rRNA*-haplotypes in 30 and 31 samples from the wild colony, respectively. Based on mitochondrial *COI* and *16s rRNA* gene sequences, the mean value of *hd* and *Pi* of all colony samples were high (*COI*: *hd* = 0.966 ***±*** 0.012 and *Pi* = 0.0556 ***±*** 0.0010; *16s rRNA*: *hd* = 0.822 ***±*** 0.021 and *Pi* = 0.0081 ***±*** 0.0005). When the samples were divided into two groups based on their origin, managed and wild colonies, *H*. *itama* samples collected from managed apiaries showed higher haplotype diversity (*hd*: *COI* = 0.985 ± 0.009; *16s rRNA = 0.808* ± 0.041), but lower nucleotide diversity (*Pi*: *COI* = 0.0575 ***±*** 0.0033; *16s rRNA =* 0.063 ± 0.0011) than those from wild populations (*hd*: *COI* = 0.860 ± 0.053; *16s rRNA =* 0.646 ± 0.058 and *Pi*: *COI* = 0.0474 ***±*** 0.0032; *16s rRNA =* 0.077 ± 0.0008). A summary of the genetic diversity indices for mitochondrial genes is presented in Table 2.

**Table 2:** Molecular diversity indices and population expansion test statistics of mitochondrial cytochrome oxidate subunit I (*COI*) and 16s ribosomal RNA (*16s rRNA*), and nuclear 28s ribosomal RNA (*28s rRNA*) gene sequence data of commercial and wild *Heterotrigona itama* population of Thailand.

Gene		*N*	*No*	*S*	*k*	*hd (±SD)*	*P*_*i*_ *(±SD)*	*D*	*Fs*	*R*_*2*_
*COI*	Province									
	Krabi	13	10	72	28.128	0.962(0.041)	0.0426(0.0036)	–0.673	1.744	0.178
	Nakhon Si Thammarat	22	17	67	17.169	0.965(0.028)	0.0260(0.0049)	–0.371	–1.635	0.119
	Narathiwat	35	15	85	26.239	0.881(0.042)	0.0398(0.0049)	0.697	–1.516	0.153
	***Colony origin***									
	Managed	39	30	116	34.663	0.985(0.009)	0.0525(0.0033)	0.603	–1.904	0.146
	Wild	31	15	80	31.267	0.860(0.053)	0.0474(0.0032)	1.650	–1.352	0.194
	***All samples***	70	***42***	***121***	***36.718***	***0.966(0.012)***	***0.0556(0.0010)***	–***1.037***	–***0.830***	***0.150***
***16s rRNA***	***Province***									
	Krabi	13	6	21	2.179	0.859(0.063)	0.00495(0.0023)	–1.293	–0.912	0.1691
	Nakhon Si Thammarat	21	4	4	0.943	0.543(0.166)	0.00214(0.0007)	–0.433	–0.093	0.1179
	Narathiwat	35	5	9	2.867	0.687(0.055)	0.00652(0.0009)	0.554	0.951	0.1574
	***Colony origin***									
	Managed	39	9	24	3.828	0.808 (0.041)	0.0063(0.0011)	–1.135	–0.396	0.0901
	Wild	30	4	9	3.386	0.646(0.058)	0.0077(0.0008)	–1.524	1.787	0.1881
	***All samples***	69	***10***	***19***	***3.557***	***0.822(0.021)***	***0.0081(0.0005)***	**–0.*443***	***1.004***	***0.0955***
***28s rRNA***	***Province***									
	Krabi	13	4	6	1.051	0.526(0.153)	0.0017(0.0007)	–1.685	–0.406	0.1556
	Nakhon Si Thammarat	21	3	2	0.505	0.467(0.113)	0.0008(0.0002)	–0.212	–0.161	0.1262
	Narathiwat	35	3	2	0.387	0.375(0.086)	0.0006(0.0002)	–0.399	–0.341	0.1171
	***Colony origin***									
	Managed	39	5	6	0.686	0.487(0.082)	0.0011(0.0003)	–1.387	–1.364	0.0837
	Wild	30	3	2	0.354	0.343(0.097)	0.0006(0.0002)	–0.612	–0.594	0.1150
	***All samples***	***69***	***5***	***6***	***0.537***	***0.422(0.064)***	***0.0009(0.0002)***	–1.364	–1.537*	***0.0660***
**Concatenated genes**	***Province***									
	Krabi	13	11	88	31.359	0.974(0.039)	0.0184(0.0015)	–0.263	0.779	0.1540
	Nakhon Si Thammarat	20	17	73	18.421	0.979(0.024)	0.0108(0.0021)	–0.525	–2.453	0.1133
	Narathiwat	35	20	96	29.492	0.946(0.024)	0.0173(0.0021)	–0.669	2.534	0.1513
	***Colony origin***									
	Managed	39	33	139	38.132	0.991(0.008)	0.0224(0.0014)	0.277	–3.875	0.1323
	Wild	29	18	91	33.542	0.931(0.034)	0.0197(0.0017)	1.321	–2.768	0.1785
	***All samples***	***68***	***48***	***146***	***40.749***	***0.983(0.007)***	***0.0239(0.0004)***	–***0.711***	***–3.550***	***0.1365***

Number of individuals (*N*), number of haplotypes (*No*), number of polymorphic (segregation) sites (*S*), average number of nucleotide differences (*k*), haplotype diversity (*hd*) and nucleotide diversity (Pi) with standard deviation (*SD*), Tajima’s *D*, Fu’s *Fs* and Ramos-Onsins and Rozas’ *R*_*2*_.

* indicate significant at *p*<0.05.

For the nuclear *28s rRNA* gene, 605 bp were analyzed from 69 individual bees in the final dataset. Unlike mitochondrial genes, a high G + C nucleotide content of 64.5% was detected in the *28s rRNA* genes. Six variable nucleotide sites were identified, including two parsimony-informative sites and four singleton sites. The total number of unique haplotypes of the *28s rRNA* gene was five. The managed colony samples revealed six haplotypes among the 39 samples, whereas the wild colony samples showed only two haplotypes among the 30 samples. All genetic diversity indices for the *28s rRNA* gene are presented in Table 2, indicating a moderately high mean value of haplotype diversity (*hd* = 0.422 ± 0.064) but low nucleotide diversity (*Pi* = 0.0009 ± 0.0002). Upon grouping the samples based on colony management, the stingless bees collected from managed apiaries exhibited higher haplotype diversity (*hd* = 0.487 ± 0.082) but lower nucleotide diversity (*Pi* = 0.0011±0.0003) compared to samples collected from wild colonies (*hd* = 0.343±0.097 and *Pi* = 0.0009±0.0002).

The three gene sequences were concatenated to a total length of 1705 bp, and 48 haplotypes were identified (Table 2). Twelve haplotypes were identified in two or more individuals, whereas 36 haplotypes were unique to one individual. The most common haplotype (h43) was identified in seven samples. We found a higher number of haplotypes in the managed population than in the wild stingless bees. We also found no correlation between *Pi* and the sample size (*r* = 0.412; *p* = 0.214), which allowed us to compare *Pi* between and among populations. Both mtDNAs and the nDNA diversity was high in both the managed (*Pi* = 0.0224±0.0014) and the wild (*Pi* = 0.0194±0.0017) colonies of *H*. *itama* samples collected from Thailand (Table 2).

### 3.2 Neutrality test

To assess the neutrality of the population, Tajima’s *D*, Fu’s *Fs*, Ramos-Onsins, and Rozas’ *R*_*2*_ statistics were obtained and are presented in Table 2. When considering all *H*. *itama* samples as a single group, Tajima’s *D* exhibited a negative and non-significant value (*p* > 0.05) for all genes. Conversely, a significantly large negative Fu’s *Fs* value was observed in the nuclear *28s rRNA* gene (*Fs* = −1.537; *p* = 0.032), while negative and non-significant values were detected for mitochondrial genes (*p* > 0.05). This suggests that excess rare alleles were specifically identified in the nuclear genes of the *H*. *itama* population in Thailand. Additionally, the Ramos-Onsins and Rozas *R*_*2*_ values were small and positive for all gene sequence data, indicating population growth of *H*. *itama* (Table 2).

When the samples were separated into three provincial populations, non-significant negative Tajima’s *D* and Fu’s *Fs* values were found. Only samples from Narathiwat Province had positive Tajima’s *D* values calculated from mitochondrial genes, indicating a demographically stable population; however, these values were not significant (Table 2). The Ramos–Onsin and Rozas’ *R*_*2*_ values were positive, but none were significant.

When considering the *H*. *itama* samples as two populations based on colony management activities (managed and wild colonies), most values of Tajima’s *D* and Fu’s *Fs* were negative and those of Ramos-Onsins and Rozas’ *R*_*2*_ were positive; however, none of them were significant (Table 2). In summary, most *H*. *itama* populations in Thailand showed no signs of expansion.

### 3.3 Genetic differentiation, gene flow estimates and analysis of molecular variation

We examined the genetic differentiation among *H*. *itama* populations in Thailand using pairwise *F*_*st*_ values. The stingless bee samples were designated as sampling locations within each province (S1 Table). The estimated values of *F*_*st*_ and per-generation migration rates (*Nm*) were calculated and are presented in Table 3.

**Table 3:** Genetic distance (*F*_*st*_) values and the estimate of migration rate (*Nm*) among populations of *Heterotrigona itama* in Thailand. Population abbreviations as in S1 Table.

	KMB	KMK	KKK	NKT	NKN	NKL	NKC	NRY	NRT	NRC	NRW	NRS
**KMB**	−	1.035	4.75	4.038	4.038	2.25	3.232	2.25	2.984	4.038	1.879	0.650
**KMK**	0.1945	−	1.579	1.249	1.249	0.500	1.479	1.035	1.400	1.249	0.944	0.408
**KKK**	0.0500	0.13669	−	*inf*	*inf*	*inf*	8.282	4.75	7.017	*inf*	3.347	0.816
**NKT**	0.0583	0.16667	0.0000	−	*inf*	*inf*	7.303	4.038	6.160	*inf*	2.852	0.670
**NKN**	0.0583	0.16667	0.0000	0.0000	−	*inf*	7.303	4.038	6.160	*inf*	2.852	0.670
**NKL**	0.1000	0.3333	0.0000	0.0000	0.0000	−	4.623	2.25	3.875	*inf*	1.625	0.375
**NKC**	0.0718*	0.1446*	0.0293	0.0331	0.0331	0.0513	−	3.232	4.222	7.303	2.613	0.903
**NRY**	0.1000	0.1945	0.0500	0.0583	0.0583	0.1000	0.0718*	−	2.984	2.588	1.879	0.650
**NRT**	0.0773	0.1515*	0.0344	0.0390	0.03890	0.0606	0.0559**	0.0773	−	6.177	2.444	0.865
**NRC**	0.0583	0.1667	0.0000	0.0000	0.0000	0.0000	0.0331	-0.0881	0.0389	−	2.852	0.670
**NRW**	0.1174	0.2093	0.0695	0.0806	0.0806	0.1333	0.0873*	0.1174	0.0928*	0.0806	−	0.986
**NRS**	0.2777**	0.3800**	0.2345**	0.2716	0.2716	0.4000	0.2169**	0.2777**	0.2242*	0.2716	0.2022*	−

The data above and below the diagonal correspond to *Nm* and *F*_*st*_, respectively.

*Inf*, infinite, * and ** indicate significant difference at *p* < 0.05 and *p* < 0.01, respectively.

Based on the mitochondrial *COI* and *16S rRNA* genes and nuclear *28S rRNA*, the pairwise *F*_*st*_ among the 66 pairs of *H*. *itama* populations ranged from 0–0.4, indicating low to moderately high genetic differentiation. For most of the genetically differentiated pairs (14 pairs), the differences were not significant (*p* > 0.05), suggesting that several population pairs of *H*. *itama* established a unique genetic group. The highest value (*F*_*st*_ = 0.4000), which was not significant (*p* = 0.18), was observed between the Nakhon Si Thammarat (Lan Saka) and Narathiwat (Sukhirin) provinces (Table 3). Surprisingly, the stingless bee samples collected from Sukhirin district, Narathiwat Province (NRS), showed statistically significant genetic differentiation when compared to other locations, except for Tha Ngio subdistrict, (NKT), Na Khian subdistrict (NKN), and Lan Saka district (NKL) of Nakhon Si Thammarat province, and Cho-airong district (NRC) of Narathiwat province (*p* > 0.05; Table 3). According to the pairwise *Nm* values, most population pairs had values greater than one, with the exception of the pair between the NRS and the other populations (ranging from 0.375–0.986; Table 3).

According to the AMOVA analysis based on the mitochondrial gene dataset, the results showed that most of the genetic variation was distributed among populations within the province (*COI*: 40.59%, *F*_*st*_ = 0.719, *p* < 0.01; *16S rRNA*: 37.51%, *F*_*st*_ = 0.749, *p* < 0.01) rather than within the population (*COI*: 28.09%; *16S rRNA*: 25.09%; Table 4). In contrast to the nuclear *28S rRNA* gene sequences, a greater proportion of variation (98.25%, *F*_*st*_ = 0.019, *p* > 0.05) was distributed within the population and only 1.40% was distributed among the populations (Table 4). After concatenating all the genes, AMOVA showed that 40.58% (*F*_*st*_ = 0.719, *p* < 0.01) of the variation was among populations within the province rather than within (28.11%) or among (31.31%) populations (Table 4).

**Table 4:** Analysis of molecular variance (AMOVA) using mitochondrial cytochrome c oxidase subunit-I gene (*COI*), the mitochondrial large ribosomal subunit rRNA gene (*16S rRNA*), and the nuclear 28S large ribosomal subunit rDNA gene (*28S rRNA*) among *Heterotrigona itama* populations under grouping criteria of (A) geographical provinces within Thai populations (Krabi, Nakhon Si Thammarat, and Narathiwat), and (B) colony types (wild and managed colonies).

Gene	Source of variation	*df*	Sum of squares	Variance components	Percentage of variation	Statistics
***COI***						
	**(A) Provinces within Thailand**					
	Among provinces	2	471.687	6.89813	31.32	*F*_*ct*_ = 0.31324
	Among populations within province	7	423.966	8.93844	40.59	*F*_*sc*_ = 0.59101**
	Within population	60	371.133	6.18556	28.09	*F*_*st*_ = 0.71912**
	**(B) Colony types**					
	Among colony types	1	139.196	0.42828	2.14	*F*_*ct*_ = 0.02137
	Among populations within colony types	9	758.674	13.36398	66.67	*F*_*sc*_ = 0.68125**
	Within population	59	368.915	6.25280	31.19	*F*_*st*_ = 0.68806**
***16s***						
	**(A) Provinces within Thailand**					
	Among provinces	2	49.694	0.81546	37.40	*F*_*ct*_ = 0.37401
	Among populations within province	7	38.972	0.81779	37.51	*F*_*sc*_ = 0.59918**
	Within population	59	32.276	0.54705	25.09	*F*_*st*_ = 0.74909**
	**(B) Colony types**					
	Among colony types	1	18.454	0.22299	11.02	*F*_*ct*_ = 0.11018
	Among populations within colony types	9	69.463	1.25085	61.81	*F*_*sc*_ = 0.69461**
	Within population	57	31.348	0.54996	27.17	*F*_*st*_ = 0.72825**
***28S***						
	**(A) Provinces within Thailand**					
	Among provinces	2	0.212	0.05091	1.40	*F*_*ct*_ = 0.03182
	Among populations within province	7	2.059	0.00682	0.31	*F*_*sc*_ = 0.00244
	Within population	59	14.700	0.34170	98.29	*F*_*st*_ = 0.01874
	**(B) Colony types**					
	Among colony types	1	0.102	0.00509	1.91	*Fct* = 0.01912
	Among populations within colony types	9	2.459	0.00044	0.16	*F*_*sc*_ = 0.00161
	Within population	58	15.700	0.27069	98.65	*F*_*st*_ = 0.01748
**Concatenated dataset**						
	**(A) Provinces within Thailand**					
	Among provinces	2	500.563	7.64717	31.31	*F*_*ct*_ = 0.31310
	Among populations within province	7	466.264	9.91067	40.58	*F*_*sc*_ = 0.59072**
	Within population	58	398.262	6.86658	28.11	*F*_*st*_ = 0.71886**
	**(B) Colony types**					
	Among colony types	1	170.989	1.36739	6.05	*Fct* = 0.06055
	Among populations within colony types	9	797.617	14.26099	63.15	*F*_*sc*_ = 0.67216**
	Within population	57	396.482	6.95582	30.80	*F*_*st*_ = 0.69201**

When the samples were grouped based on colony management, the results of the AMOVA based on mitochondrial genes demonstrated that more than 60% of the genetic variation was among populations within colony types. Consistent with these results, AMOVA based on all concatenated genes revealed that the greatest amount of variation (63.15%) was distributed among populations within colony types, with only 6.05% variation among colony types (*F*_*st*_ = 0.692; *p* < 0.01; Table 4). In contrast, the estimated genetic differentiation among the colony type groups based on the nuclear gene dataset was very low (*F*_*st*_ = 0.0175) and not statistically significant (*p* > 0.05; Table 4).

The number of base substitutions per site was calculated for both mitochondrial and nuclear genes by averaging all sequence pairs between populations using the Kimura 2-parameter (K2P) model, and is presented in Table 5. The K2P values for the 66 population pairs ranged from 0.033 [(Khlong yang subdistrict, Ko Lanta district, Krabi province (KKK)/ NKT)] to 3.789% [(NKT/ Yi-ngo district, Narathiwat province (NRY)]. Additionally, we estimated the pairwise index of differentiation (*D*_*est*_) by concatenating all the gene datasets (Table 6). The average *D*_*est*_ value was high (0.637, *p* < 0.01) and ranged from 0.0121 [(NKT/ Tak Bai district, Narathiwat province (NRT)] to 0.9884 [(NKL/ Sukhirin district, Narathiwat province (NRS)], as presented in Table 6. An elevated *D*_*est*_ index indicates a highly significant population structure.

**Table 5:** The evolutionary divergence over sequence pairs between populations of *Heterotrigona itama* were estimated. The number of base substitutions per site from averaging over all sequence pairs between populations were estimated using Kimura 2-parameter model (Kimura, 1980) and shown. Population abbreviations as in S1 Table.

	KMB	KMK	KKK	NKT	NKN	NKL	NKC	NRY	NRT	NRC	NRW
**KMB**	−										
**KMK**	0.00876	−									
**KKK**	0.02169	0.00874	−								
**NKT**	0.01146	0.00100	0.00033	−							
**NKN**	0.00680	0.00137	0.01480	0.00280	−						
**NKL**	0.02628	0.01251	0.00663	0.00606	0.01935	−					
**NKC**	0.00661	0.00597	0.01901	0.00862	0.00230	0.02334	−				
**NRY**	0.03789	0.02874	0.03232	0.02737	0.03201	0.03249	0.03585	−			
**NRT**	0.01675	0.00401	0.00397	0.00039	0.00922	0.00375	0.01398	0.02019	−		
**NRC**	0.03503	0.02620	0.02954	0.02457	0.02959	0.02991	0.03307	0.00040	0.01753	−	
**NRW**	0.03347	0.02477	0.02855	0.02340	0.02837	0.03009	0.03171	0.00370	0.01685	0.00108	−
**NRS**	0.03511	0.02639	0.03057	0.02522	0.03007	0.03347	0.03357	0.00881	0.01957	0.00610	0.00139

**Table 6:** Pairwise of genetic differentiation (D_est_) from 1,705 bp concatenated alignment dataset of *COI* and *16S rRNA* mitochondrial genes, and *28S rRNA* nuclear gene of *Heterotrigona itama*. Population abbreviations as in S1 Table.

	KMB	KMK	KKK	NKT	NKN	NKL	NKC	NRY	NRT	NRC	NRW
**KMK**	0.5196										
**KKK**	0.8131	0.4984									
**NKT**	0.6009	0.0567	0.1119								
**NKN**	0.5246	0.1029	0.6751	0.1741							
**NKL**	0.8436	0.1531	0.4125	0.1268	0.5591						
**NKC**	0.6285	0.5770	0.8200	0.6526	0.4072	0.8509					
**NRY**	0.9308	0.8132	0.9063	0.8262	0.8812	0.9645	0.9197				
**NRT**	0.5386	0.1956	0.1887	0.0121	0.3430	0.3886	0.5812	0.5999			
**NRC**	0.8911	0.7016	0.8552	0.7154	0.7993	0.877	0.8974	0.0769	0.5122		
**NRW**	0.8762	0.7484	0.8463	0.7537	0.8133	0.8493	0.8857	0.5678	0.5556	0.2578	
**NRS**	0.9556	0.8868	0.9401	0.8957	0.9315	0.9884	0.9354	0.9334	0.6705	0.8640	0.3604

Colours highlight D_est_ values. Green: D_est_ < 0.1; yellow: 0.1 < D_est_ < 0.25; orang: 0.25 < D_est_ < 0.50; red: D_est_ > 0. 50.

### 3.4 Isolation by distance

We calculated the geographical distance between the populations based on the coordinates of each population. The geographical distances between the pairs of populations ranged from approximately 7–425 km. Additionally, we identified a significant positive correlation between the genetic and geographic distances (*r* = 0.2142, *p* = 0.024, n = 66).

### 3.5 Phylogenetic tree and haplotype network analysis

**Fig 2:**
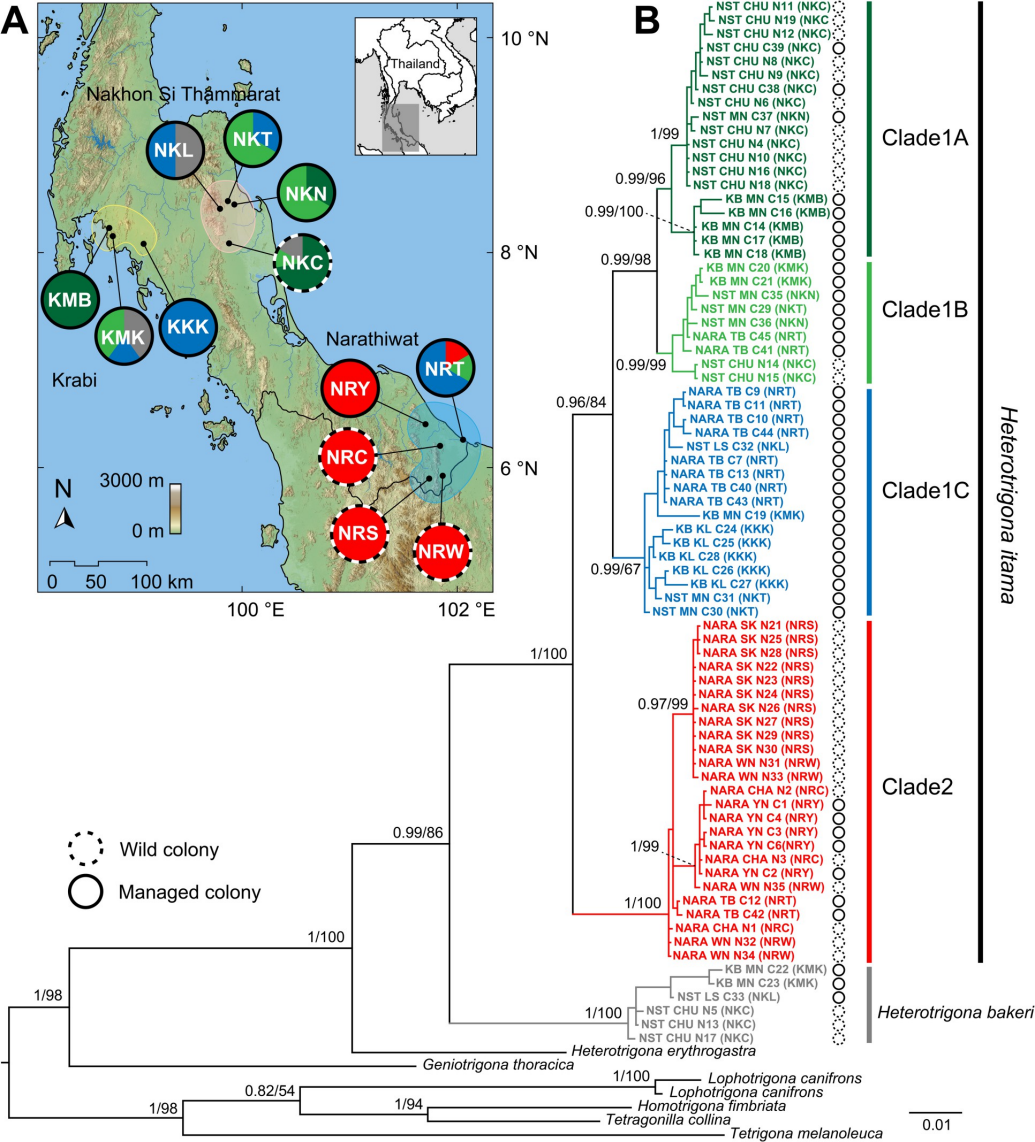
(A) Map of southern Thailand showing collecting sites of *Heterotrigona itama* with abbreviation of locality name as list in S1 Table. Pie charts represent proportion of the hypothetical clades of *H*. *itama* from phylogenetic analyses. (B) Bayesian inference (BI) tree of *H*. *itama* and related species based on 1,705 bp concatenated alignment dataset of COI and 16S rRNA mitochondrial genes, and 28S rRNA nuclear gene. The numbers on notes represent the bipartition posterior probability (bpp) from the BI analysis and the bootstrap supports (BS) from the ML analysis, and are shown as BI/MLL. Scale bar indicates the branch length. Map was generated using QGIS v3.24.3 with the river and lake topology from the HydroSHEDS database (https://www.hydrosheds.org), and the map raster data from the NASA EARTHDATA (https://www.earthdata.nasa.gov/).

Phylogenetic analyses were conducted using concatenated datasets of *COI*, *16S rRNA*, and *28S rRNA* sequences. Both ML and BI methods produced nearly identical tree topologies with only minor differences in the arrangement of the tip clades. Therefore, only the BI tree is shown in Fig 2B. The phylogenetic tree clearly illustrates the monophyly of *H*. *itama* (bpp = 1, BS = 100%) and places it as a sister clade to *H*. *bakeri*, albeit with support primarily from the BI analysis (bpp = 0.99, BS = 86%). The clade comprising *H*. *bakeri* included specimens from three localities in two provinces (Fig 2A), with colonies occurring sympatrically with *H*. *itama* and including specimens collected from both wild and managed colonies. The *H*. *itama* clade was initially subdivided into two major clades: Clade 1 and Clade 2 (Fig 2B). Clade 1 comprised specimens collected from all three provinces, with moderate support from the ML analysis (bpp = 0.96, BS = 84%) and was subsequently further divided into three sub-clades. Clades 1A and Clade 1B formed a sister clade (bpp = 0.99, BS = 98%) followed by Clade 1C at the basal position. Clades 1A and 1B received strong support from both ML and BI analyses (bpp = 0.99, BS = 98–99%), whereas Clade 1C was primarily supported by the BI analysis (bpp = 0.99, BS = 67%). Specimens in Clade 1C were collected exclusively from managed colonies, whereas Clades 1A and 1B comprised samples from both wild and managed colony types. Within Clade 1B, samples from the wild colony were positioned at the basal portion of the clade, distinct from other specimens from the managed colonies. Conversely, samples from both colony types were interspersed throughout Clade 1A, except for specimens from KMB, all of which originated from managed colonies. Clade 2 of *H*. *itama* consisted solely of colonies from Narathiwat Province, with strong support from both the ML and BI analyses (bpp = 1, BS = 100%). There was no strong evidence of differentiation between wild and managed colonies within this clade.

**Fig 3:**
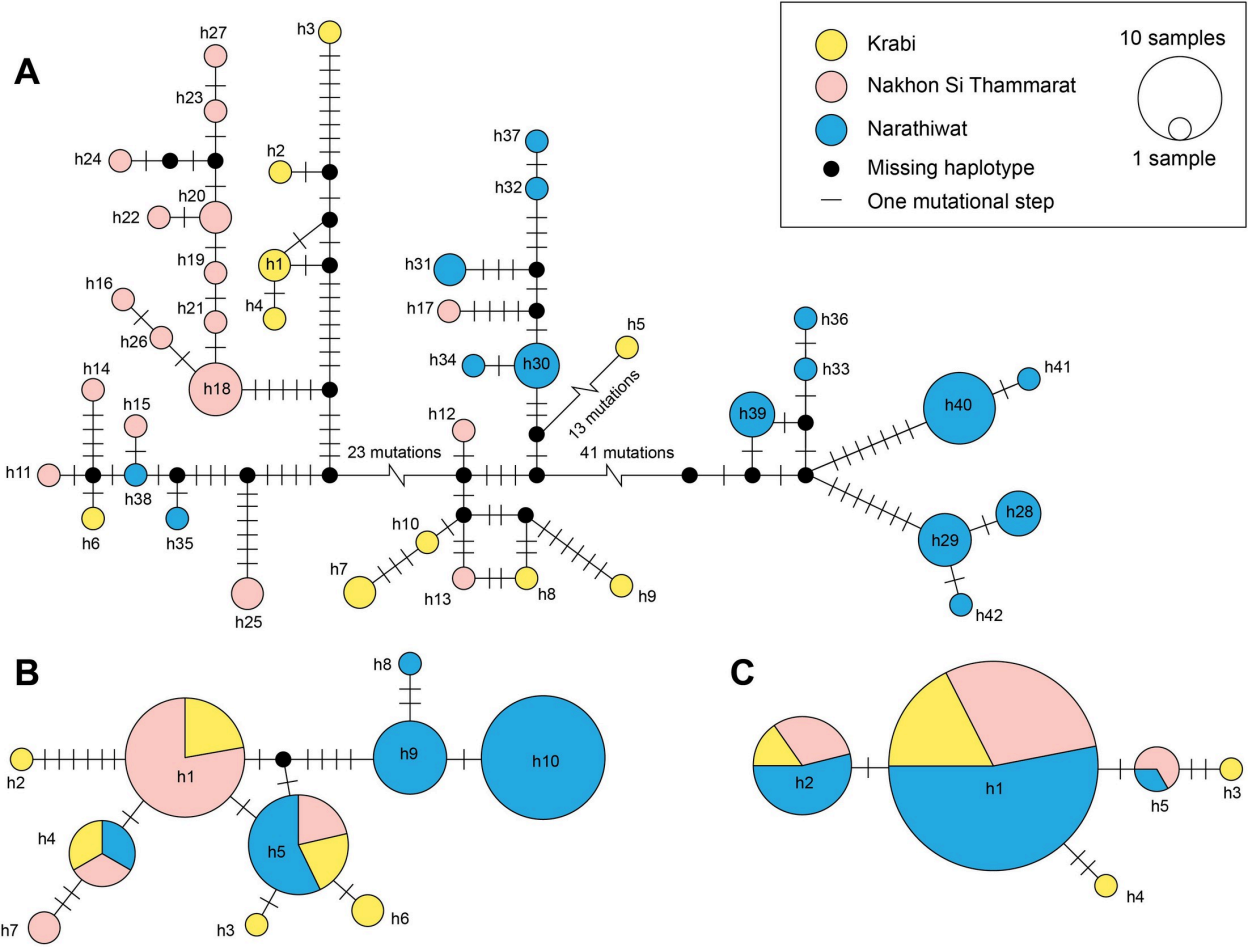
Haplotype network of *Heterotrigona itama* for *COI* (A), *16S rRNA* (B), and *28S rRNA* (C) genes. The three populations of *H*. *itama* are represented by colors according to the legend. Each circle represents one unique haplotype, with the size of circles proportional to sample sizes. Crossbars demonstrate on mutation step.

Median-joining networks were constructed separately for each gene to elucidate the genetic relationships among *H*. *itama* haplotypes from the three provinces, as depicted in Fig 3. The resulting haplotype networks for *COI* and *16S rRNA* closely mirrored the phylogenetic trees, although the *16S rRNA* network displayed a lower-resolution structure. The mitochondrial *COI* network exhibited a highly structured pattern, comprising 40 unique haplotypes corresponding to the groupings observed in the phylogenetic tree (Fig 3A). Each *COI* haplotype consisted of specimens collected exclusively from one area, with no haplotypes shared across provinces. The haplotype group in Clade 1 was distinguished from that of Clade 2 by 41 mutational steps. Within the haplotype group of Clade 1, Clade 1A was situated close to Clade 1B, differing by only eight mutational steps, whereas it was separated from Clade1C by more than 23 mutational steps. Notably, haplotype *COI* h5 from the Krabi province differed from the other haplotypes in Clade1C by at least 13 mutational steps.

The *16S rRNA* dataset comprised ten unique haplotypes (Fig 3B). The separation between Clade 1 and Clade 2 in the *16S rRNA* network was delineated by six mutational steps, with no apparent differentiation among the three subclades of Clade 1. The most prevalent haplotype was h1, collected from Krabi and Nakhon Si Thammarat provinces, encompassing 18 samples, followed by h10, with 17 samples from Narathiwat Province. Two haplotypes (h4 and h5) were shared among the samples from all provinces.

The *28S rRNA* network exhibited the fewest unique haplotypes (five; Fig 3C), reflecting the slow evolutionary rate of the *28S rRNA* gene. They were distinguished by to 1–3 mutational changes, with the most common *28S rRNA* haplotype, h1, comprising 51 sequences. The hi haplotype was connected to the second most common haplotype, h2, via a single mutational step. Both haplotypes were found in samples from all provinces. Additionally, two rare *28S rRNA* haplotypes, h3 and h4, were also identified. Each haplotype contained a single sample from the Krabi province.

## 4. Discussion

Several previous studies have reported that artificial breeding selection can lead to an increase in inbreeding and a reduction in genetic variability relative to wild progenitors [70–72]. Beekeepers in Thailand practice a minor level of artificial selection on *H*. *itama*, such as preferentially propagating high honey-producing colonies. However, free mating of queens and the import of outside colonies have likely introduced new genetic material. The present study demonstrated that wild and managed populations of *H*. *itama* in Thailand exhibit high genetic variability, suggesting that the colony management practices of Thai beekeepers do not significantly affect mitochondrial or nuclear variability in the *H*. *itama* populations.

Geographical and physical barriers (e.g., mountain range, the presence of agricultural or urban areas, and the presence or absence of forests) appeared to have no effect on the population structuring in *H*. *itama* populations in the present study. The population of stingless bees tended to deviate when there are no barriers to gene flow. However, the low dispersal of female queens combined with isolation by distance, likely act as the main factors shaping the population structure of *H*. *itama* as detected in this study, similar to what have been found in *Trigona nigerrima* and *Trigona corvina* from Mexico [73], *Tetragonula carbonaria* from Australia [74], and *Trigona spinipes* from Brazil [75]. Whilst, the gene flow over long geographical distance may drive by drone dispersal for seeking a mating with a distance virgin queen [76].

For artificial colony division, aimed at increasing the colony number in a commercial apiary, young and old brood combs, along with honey and pollen pots, are transferred from a strong colony to a new hive box, resulting in the growth of a new daughter colony [1, 31]. A consequence of this division method is an increase in the frequency of some mitochondrial haplotypes, while others may decrease in frequency or be lost [31]. We detected this pattern as a high population structure and a low number of haplotypes with a high haplotype frequency in the Narathiwat population (Table 2 and Fig 2A). Since mitochondrial haplotypes are maternally inherited, they can move among populations when a colony from one population successfully establishes as a daughter colony in other populations [12]. This results in the structuring of mitochondrial haplotypes [77]. However, the high mitochondrial structure observed in wild populations has been attributed to the short reproductive swarm distance of the daughter colony, a phenomenon known as female queen philopatric behavior. This behavior occurs because the daughter colony requires resources, such as propolis and food, from the mother colony to build a new nest [78]. Therefore, the female queen’s philopatric behavior limits the dispersal of the population [31]. In managed populations, colony division from a small number of colonies in the apiary could result in a genetic outcome similar to that of female queen philopatric behavior [31]. High structuring of the population, based on mitochondrial sequence analyses, was also detected in wild populations of several stingless bee species, including *Melipona beecheii* [1, 79], *Partamona helleri* [80], *Plebeia remota* [81, 82], *T*. *pagdeni* [83], *Scaptotrigona hellwegeri* [84], *Partamona mulata* [85], *Melipona subnitida* [86], and *Tetragonisca angustula* [87].

Unlike stingless bee queens, drones disperse over long distance [12, 88, 89]. Drones leave their colonies and form “drone congregations” near other colonies that have virgin queens [1]. Meliponine drone congregations exhibit the presence of drones from long-distance sites and may include several hundred drones that originate from several different colonies [88–90]. Although drones of stingless bees have limited dispersal efficiency in comparison to honey bees of the genus *Apis* [91–93], the low genetic structuring and genetic distance between Nakhon Si Thammarat and Krabi suggest that it can be attributed to drone dispersal (Table 3 and Fig 2).

Nonetheless, we found that some *H*. *itama* populations in the present study were not well differentiated from other populations. This is likely due to gene flow, possibly following the stepping-stone model [94], although colony displacement by beekeepers also likely plays an important role. For instance, the bee samples in Clades 1A, 1 B, and 1C, all found in Nakhon Si Thammarat, were similar to those found in the Narathiwat population (Fig 2). Additionally, the *COI* haplotypes h13, h15, and h17 found in Nakhon Si Thammarat were closely related to those found in other provinces (Fig 3A). Due to the high frequency of unique haplotypes detected in Nakhon Si Thammarat, we suggest that colonies of *H*. *itama* were transported from elsewhere to Nakhon Si Thammarat, resulting in an artificial increase in the genetic diversity of this population.

Genetic isolation causes low genetic diversity in organism [95]. The low diversity values detected in the Nakhon Si Thammarat population suggest that population bottlenecks exist. Indeed, the structured pattern of *the H*. *itama* haplotype network and the high value of haplotype diversity in contrast with the low nucleotide diversity that we observed indicates evidence of relatively recent recolonization and population expansion (Table 2 and Fig 3).

The results of the present study demonstrate that most *H*. *itama* populations are genetically differentiated. Although we found no evidence that the trade of stingless bees affected population structure of these stingless bee populations in this region, an increase in these activities among genetically differentiated populations could lead to negative genetic consequences. Hence, it is crucial to ensure that beekeepers understand and implement appropriate apiary practices to preserve local genetic characteristics, as native stocks have evolved alleles adapted to local conditions. Therefore, we suggest a special investigation before introducing new ecotypes into new areas, where colony displacement should occur only between populations that are genetically similar.

## Supporting information

S1 Table Locality with geographic coordinates, colony type, hypothetical clade, and GenBank accession numbers for specimens used in phylogenetic analysis. (DOCX)

S1 File Concatenated sequence alignment data (*COI* + *16S rRNA* +*28S rRNA*) used in phylogenetic analyses. (DOCX)

S2 File In put file of the *COI* gene dataset used in haplotype network analysis. (DOCX)

S3 File In put file of the *16S rRNA* gene dataset used in haplotype network analysis. (DOCX)

S4 File In put file of the *28S rRNA* gene dataset used in haplotype network analysis. (DOCX)
