# Supplementary material for: Genetic structure of the commercial stingless bee Heterotrigona itama (Apidae: Meliponini) in Thailand
Source: PLoS One. 2024 Dec 4;19(12):e0312386. doi: 10.1371/journal.pone.0312386 (PMC11616864; doi:10.1371/journal.pone.0312386)
Supplement: S1 Table — (DOCX) [file pone.0312386.s001.docx]

**Supplementary Table S1** Locality with geographic coordinates, colony type, hypothetical clade, and GenBank accession numbers for specimens used in phylogenetic analysis.

| **Taxa** | **Sample code** | **Localities** | **Locality code** | **Geographic coordinates** | **Colony type** | **Clade** | **Genbank acession** | | |
| --- | --- | --- | --- | --- | --- | --- | --- | --- | --- |
|  |  |  |  |  |  |  | **COI** | **16S** | **28S** |
| *Heterotrigona itama* (Cockerell 1918) | KB MN C14 | Ban nai nang, Mueang, Krabi, Thailand | KMB | 8°13′46″N, 98°45′55″E | Managed | 1A | OR951935 | OQ102788 | OQ102866 |
| *Heterotrigona itama* (Cockerell 1918) | KB MN C15 | Ban nai nang, Mueang, Krabi, Thailand | KMB | 8°13′46″N, 98°45′55″E | Managed | 1A | OR951936 | OQ102789 | OQ102867 |
| *Heterotrigona itama* (Cockerell 1918) | KB MN C16 | Ban nai nang, Mueang, Krabi, Thailand | KMB | 8°13′46″N, 98°45′55″E | Managed | 1A | OR951937 | OQ102790 | OQ102868 |
| *Heterotrigona itama* (Cockerell 1918) | KB MN C17 | Ban nai nang, Mueang, Krabi, Thailand | KMB | 8°13′46″N, 98°45′55″E | Managed | 1A | OR951938 | OQ102791 | OQ102869 |
| *Heterotrigona itama* (Cockerell 1918) | KB MN C18 | Ban nai nang, Mueang, Krabi, Thailand | KMB | 8°13′46″N, 98°45′55″E | Managed | 1A | OR951939 | OQ102792 | OQ102870 |
| *Heterotrigona itama* (Cockerell 1918) | KB MN C19 | Khao khom, Mueang, Krabi, Thailand | KMK | 8°09′12″N, 98°47′52″E | Managed | 1C | OR951940 | OQ102793 | OQ102871 |
| *Heterotrigona itama* (Cockerell 1918) | KB MN C20 | Khao khom, Mueang, Krabi, Thailand | KMK | 8°09′12″N, 98°47′52″E | Managed | 1B | OR951941 | OQ102794 | OQ102872 |
| *Heterotrigona itama* (Cockerell 1918) | KB MN C21 | Khao khom, Mueang, Krabi, Thailand | KMK | 8°09′12″N, 98°47′52″E | Managed | 1B | OR951942 | OQ102795 | OQ102873 |
| *Heterotrigona itama* (Cockerell 1918) | KB KL C24 | Khlong yang, Ko Lanta, Krabi, Thailand | KKK | 8°04′54″N, 99°05′05″E | Managed | 1C | OR951943 | OQ102798 | OQ102876 |
| *Heterotrigona itama* (Cockerell 1918) | KB KL C25 | Khlong yang, Ko Lanta, Krabi, Thailand | KKK | 8°04′54″N, 99°05′05″E | Managed | 1C | OR951944 | OQ102799 | OQ102877 |
| *Heterotrigona itama* (Cockerell 1918) | KB KL C26 | Khlong yang, Ko Lanta, Krabi, Thailand | KKK | 8°04′54″N, 99°05′05″E | Managed | 1C | OR951945 | OQ102800 | OQ102878 |
| *Heterotrigona itama* (Cockerell 1918) | KB KL C27 | Khlong yang, Ko Lanta, Krabi, Thailand | KKK | 8°04′54″N, 99°05′05″E | Managed | 1C | OR951946 | OQ102801 | OQ102879 |
| *Heterotrigona itama* (Cockerell 1918) | KB KL C28 | Khlong yang, Ko Lanta, Krabi, Thailand | KKK | 8°04′54″N, 99°05′05″E | Managed | 1C | OR951947 | OQ102802 | OQ102880 |
| *Heterotrigona itama* (Cockerell 1918) | NST MN C29 | Tha Ngio, Mueang, Nakhon Si Thammarat, Thailand | NKT | 8°28′46″N, 99°52′05″E | Managed | 1B | OR951948 | OQ102803 | OQ102881 |
| *Heterotrigona itama* (Cockerell 1918) | NST MN C30 | Tha Ngio, Mueang, Nakhon Si Thammarat, Thailand | NKT | 8°28′46″N, 99°52′05″E | Managed | 1C | OR951949 | OQ102804 | OQ102882 |
| *Heterotrigona itama* (Cockerell 1918) | NST MN C31 | Tha Ngio, Mueang, Nakhon Si Thammarat, Thailand | NKT | 8°28′46″N, 99°52′05″E | Managed | 1C | OR951950 | OQ102805 | OQ102883 |
| *Heterotrigona itama* (Cockerell 1918) | NST MN C35 | Na Khian, Mueang, Nakhon Si Thammarat, Thailand | NKN | 8°26′52″N, 99°55′43″E | Managed | 1B | OR951952 | OQ102808 | OQ102887 |
| *Heterotrigona itama* (Cockerell 1918) | NST MN C36 | Na Khian, Mueang, Nakhon Si Thammarat, Thailand | NKN | 8°26′52″N, 99°55′43″E | Managed | 1B | OR951953 | OQ102809 | OQ102888 |
| *Heterotrigona itama* (Cockerell 1918) | NST MN C37 | Na Khian, Mueang, Nakhon Si Thammarat, Thailand | NKN | 8°26′52″N, 99°55′43″E | Managed | 1A | OR951954 | OQ102810 | OQ102889 |
| *Heterotrigona itama* (Cockerell 1918) | NST LS C32 | Kamlon, Lan Saka, Nakhon Si Thammarat, Thailand | NKL | 8°24′29″N, 99°47′42″E | Managed | 1C | OR951951 | OQ102806 | OQ102884 |
| *Heterotrigona itama* (Cockerell 1918) | NST CHU N4 | Chulabhorn District, Nakhon Si Thammarat, Thailand | NKC | 8°05′09″N, 99°52′46″E | Wild | 1A | OR951955 | OQ102811 | OQ102890 |
| *Heterotrigona itama* (Cockerell 1918) | NST CHU C38 | Chulabhorn District, Nakhon Si Thammarat, Thailand | NKC | 8°05′09″N, 99°52′46″E | Managed | 1A | OR951956 | OQ102813 | OQ102892 |
| *Heterotrigona itama* (Cockerell 1918) | NST CHU C39 | Chulabhorn District, Nakhon Si Thammarat, Thailand | NKC | 8°05′09″N, 99°52′46″E | Managed | 1A | OR951957 | OQ102814 | OQ102893 |
| *Heterotrigona itama* (Cockerell 1918) | NST CHU N6 | Chulabhorn District, Nakhon Si Thammarat, Thailand | NKC | 8°05′09″N, 99°52′46″E | Wild | 1A | OR951958 | OQ102815 | OQ102894 |
| *Heterotrigona itama* (Cockerell 1918) | NST CHU N7 | Chulabhorn District, Nakhon Si Thammarat, Thailand | NKC | 8°05′09″N, 99°52′46″E | Wild | 1A | OR951959 | OQ102816 | OQ102895 |
| *Heterotrigona itama* (Cockerell 1918) | NST CHU N8 | Chulabhorn District, Nakhon Si Thammarat, Thailand | NKC | 8°05′09″N, 99°52′46″E | Wild | 1A | OR951960 | OQ102817 | OQ102896 |
| *Heterotrigona itama* (Cockerell 1918) | NST CHU N9 | Chulabhorn District, Nakhon Si Thammarat, Thailand | NKC | 8°05′09″N, 99°52′46″E | Wild | 1A | OR951961 | OQ102818 | OQ102897 |
| *Heterotrigona itama* (Cockerell 1918) | NST CHU N10 | Chulabhorn District, Nakhon Si Thammarat, Thailand | NKC | 8°05′09″N, 99°52′46″E | Wild | 1A | OR951962 | OQ102819 | OQ102898 |
| *Heterotrigona itama* (Cockerell 1918) | NST CHU N11 | Chulabhorn District, Nakhon Si Thammarat, Thailand | NKC | 8°05′09″N, 99°52′46″E | Wild | 1A | OR951963 | n/a | OQ102899 |
| *Heterotrigona itama* (Cockerell 1918) | NST CHU N12 | Chulabhorn District, Nakhon Si Thammarat, Thailand | NKC | 8°05′09″N, 99°52′46″E | Wild | 1A | OR951964 | OQ102820 | OQ102900 |
| *Heterotrigona itama* (Cockerell 1918) | NST CHU N14 | Chulabhorn District, Nakhon Si Thammarat, Thailand | NKC | 8°05′09″N, 99°52′46″E | Wild | 1B | OR951965 | OQ102822 | OQ102902 |
| *Heterotrigona itama* (Cockerell 1918) | NST CHU N15 | Chulabhorn District, Nakhon Si Thammarat, Thailand | NKC | 8°05′09″N, 99°52′46″E | Wild | 1B | OR951966 | OQ102823 | n/a |
| *Heterotrigona itama* (Cockerell 1918) | NST CHU N16 | Chulabhorn District, Nakhon Si Thammarat, Thailand | NKC | 8°05′09″N, 99°52′46″E | Wild | 1A | OR951967 | OQ102824 | OQ102903 |
| *Heterotrigona itama* (Cockerell 1918) | NST CHU N18 | Chulabhorn District, Nakhon Si Thammarat, Thailand | NKC | 8°05′09″N, 99°52′46″E | Wild | 1A | OR951968 | OQ102826 | OQ102905 |
| *Heterotrigona itama* (Cockerell 1918) | NST CHU N19 | Chulabhorn District, Nakhon Si Thammarat, Thailand | NKC | 8°05′09″N, 99°52′46″E | Wild | 1A | OR951969 | OQ102827 | OQ102906 |
| *Heterotrigona itama* (Cockerell 1918) | NARA YN C1 | Yi-ngo, Narathiwat, Thailand | NRY | 6°24′11″N, 101°42′22″E | Managed | 2 | OR951924 | OQ102775 | OQ102853 |
| *Heterotrigona itama* (Cockerell 1918) | NARA YN C2 | Yi-ngo, Narathiwat, Thailand | NRY | 6°24′11″N, 101°42′22″E | Managed | 2 | OR951925 | OQ102776 | OQ102854 |
| *Heterotrigona itama* (Cockerell 1918) | NARA YN C3 | Yi-ngo, Narathiwat, Thailand | NRY | 6°24′11″N, 101°42′22″E | Managed | 2 | OR951926 | OQ102777 | OQ102855 |
| *Heterotrigona itama* (Cockerell 1918) | NARA YN C4 | Yi-ngo, Narathiwat, Thailand | NRY | 6°24′11″N, 101°42′22″E | Managed | 2 | OR951927 | OQ102778 | OQ102856 |
| *Heterotrigona itama* (Cockerell 1918) | NARA YN C6 | Yi-ngo, Narathiwat, Thailand | NRY | 6°24′11″N, 101°42′22″E | Managed | 2 | OR951928 | OQ102780 | OQ102858 |
| *Heterotrigona itama* (Cockerell 1918) | NARA TB C7 | Tak Bai, Narathiwat, Thailand | NRT | 6°15′32″N, 102°3′18″E | Managed | 1C | OR951929 | OQ102781 | OQ102859 |
| *Heterotrigona itama* (Cockerell 1918) | NARA TB C9 | Tak Bai, Narathiwat, Thailand | NRT | 6°15′32″N, 102°3′18″E | Managed | 1C | OR951930 | OQ102783 | OQ102861 |
| *Heterotrigona itama* (Cockerell 1918) | NARA TB C10 | Tak Bai, Narathiwat, Thailand | NRT | 6°15′32″N, 102°3′18″E | Managed | 1C | OR951931 | OQ102784 | OQ102862 |
| *Heterotrigona itama* (Cockerell 1918) | NARA TB C11 | Tak Bai, Narathiwat, Thailand | NRT | 6°15′32″N, 102°3′18″E | Managed | 1C | OR951932 | OQ102785 | OQ102863 |
| *Heterotrigona itama* (Cockerell 1918) | NARA TB C12 | Tak Bai, Narathiwat, Thailand | NRT | 6°15′32″N, 102°3′18″E | Managed | 2 | OR951933 | OQ102786 | OQ102864 |
| *Heterotrigona itama* (Cockerell 1918) | NARA TB C13 | Tak Bai, Narathiwat, Thailand | NRT | 6°15′32″N, 102°3′18″E | Managed | 1C | OR951934 | OQ102787 | OQ102865 |
| *Heterotrigona itama* (Cockerell 1918) | NARA TB C40 | Tak Bai, Narathiwat, Thailand | NRT | 6°15′32″N, 102°3′18″E | Managed | 1C | OR951985 | OQ102843 | OQ102923 |
| *Heterotrigona itama* (Cockerell 1918) | NARA TB C41 | Tak Bai, Narathiwat, Thailand | NRT | 6°15′32″N, 102°3′18″E | Managed | 1B | OR951986 | OQ102844 | OQ102924 |
| *Heterotrigona itama* (Cockerell 1918) | NARA TB C42 | Tak Bai, Narathiwat, Thailand | NRT | 6°15′32″N, 102°3′18″E | Managed | 2 | OR951987 | OQ102845 | OQ102925 |
| *Heterotrigona itama* (Cockerell 1918) | NARA TB C43 | Tak Bai, Narathiwat, Thailand | NRT | 6°15′32″N, 102°3′18″E | Managed | 1C | OR951988 | OQ102846 | OQ102926 |
| *Heterotrigona itama* (Cockerell 1918) | NARA TB C44 | Tak Bai, Narathiwat, Thailand | NRT | 6°15′32″N, 102°3′18″E | Managed | 1C | OR951989 | OQ102847 | OQ102927 |
| *Heterotrigona itama* (Cockerell 1918) | NARA TB C45 | Tak Bai, Narathiwat, Thailand | NRT | 6°15′32″N, 102°3′18″E | Managed | 1B | OR951990 | OQ102848 | OQ102928 |
| *Heterotrigona itama* (Cockerell 1918) | NARA CHA N1 | Cho-airong, Narathiwat, Thailand | NRC | 6°12′6″N, 101°50′30″E | Wild | 2 | OR951921 | OQ102772 | OQ102850 |
| *Heterotrigona itama* (Cockerell 1918) | NARA CHA N2 | Cho-airong, Narathiwat, Thailand | NRC | 6°12′6″N, 101°50′30″E | Wild | 2 | OR951922 | OQ102773 | OQ102851 |
| *Heterotrigona itama* (Cockerell 1918) | NARA CHA N3 | Cho-airong, Narathiwat, Thailand | NRC | 6°12′6″N, 101°50′30″E | Wild | 2 | OR951923 | OQ102774 | OQ102852 |
| *Heterotrigona itama* (Cockerell 1918) | NARA WN N31 | Waeng, Narathiwat, Thailand | NRW | 5°55′25″N, 101°51′57″E | Wild | 2 | OR951980 | OQ102838 | OQ102918 |
| *Heterotrigona itama* (Cockerell 1918) | NARA WN N32 | Waeng, Narathiwat, Thailand | NRW | 5°55′25″N, 101°51′57″E | Wild | 2 | OR951981 | OQ102839 | OQ102919 |
| *Heterotrigona itama* (Cockerell 1918) | NARA WN N33 | Waeng, Narathiwat, Thailand | NRW | 5°55′25″N, 101°51′57″E | Wild | 2 | OR951982 | OQ102840 | OQ102920 |
| *Heterotrigona itama* (Cockerell 1918) | NARA WN N34 | Waeng, Narathiwat, Thailand | NRW | 5°55′25″N, 101°51′57″E | Wild | 2 | OR951983 | OQ102841 | OQ102921 |
| *Heterotrigona itama* (Cockerell 1918) | NARA WN N35 | Waeng, Narathiwat, Thailand | NRW | 5°55′25″N, 101°51′57″E | Wild | 2 | OR951984 | OQ102842 | OQ102922 |
| *Heterotrigona itama* (Cockerell 1918) | NARA SK N21 | Mamong, Sukhirin District, Narathiwat, Thailand | NRS | 5°53′49″N, 101°44′26″E | Wild | 2 | OR951970 | OQ102828 | OQ102908 |
| *Heterotrigona itama* (Cockerell 1918) | NARA SK N22 | Mamong, Sukhirin District, Narathiwat, Thailand | NRS | 5°53′49″N, 101°44′26″E | Wild | 2 | OR951971 | OQ102829 | OQ102909 |
| *Heterotrigona itama* (Cockerell 1918) | NARA SK N23 | Mamong, Sukhirin District, Narathiwat, Thailand | NRS | 5°53′49″N, 101°44′26″E | Wild | 2 | OR951972 | OQ102830 | OQ102910 |
| *Heterotrigona itama* (Cockerell 1918) | NARA SK N24 | Mamong, Sukhirin District, Narathiwat, Thailand | NRS | 5°53′49″N, 101°44′26″E | Wild | 2 | OR951973 | OQ102831 | OQ102911 |
| *Heterotrigona itama* (Cockerell 1918) | NARA SK N25 | Mamong, Sukhirin District, Narathiwat, Thailand | NRS | 5°53′49″N, 101°44′26″E | Wild | 2 | OR951974 | OQ102832 | OQ102912 |
| *Heterotrigona itama* (Cockerell 1918) | NARA SK N26 | Mamong, Sukhirin District, Narathiwat, Thailand | NRS | 5°53′49″N, 101°44′26″E | Wild | 2 | OR951975 | OQ102833 | OQ102913 |
| *Heterotrigona itama* (Cockerell 1918) | NARA SK N27 | Mamong, Sukhirin District, Narathiwat, Thailand | NRS | 5°53′49″N, 101°44′26″E | Wild | 2 | OR951976 | OQ102834 | OQ102914 |
| *Heterotrigona itama* (Cockerell 1918) | NARA SK N28 | Mamong, Sukhirin District, Narathiwat, Thailand | NRS | 5°53′49″N, 101°44′26″E | Wild | 2 | OR951977 | OQ102835 | OQ102915 |
| *Heterotrigona itama* (Cockerell 1918) | NARA SK N29 | Mamong, Sukhirin District, Narathiwat, Thailand | NRS | 5°53′49″N, 101°44′26″E | Wild | 2 | OR951978 | OQ102836 | OQ102916 |
| *Heterotrigona itama* (Cockerell 1918) | NARA SK N30 | Mamong, Sukhirin District, Narathiwat, Thailand | NRS | 5°53′49″N, 101°44′26″E | Wild | 2 | OR951979 | OQ102837 | OQ102917 |
| *Heterotrigona bakeri* (Cockerell, 1919) | KB MN C22 | Khao khom, Mueang, Krabi, Thailand | KMK | 8°09′12″N, 98°47′52″E | Managed | outgroup | OR951991 | OQ102796 | OQ102874 |
| *Heterotrigona bakeri* (Cockerell, 1919) | KB MN C23 | Khao khom, Mueang, Krabi, Thailand | KMK | 8°09′12″N, 98°47′52″E | Managed | outgroup | OR951992 | OQ102797 | OQ102875 |
| *Heterotrigona bakeri* (Cockerell, 1919) | NST LS C33 | Kamlon, Lan Saka, Nakhon Si Thammarat, Thailand | NKL | 8°24′29″N, 99°47′42″E | Managed | outgroup | OR951993 | n/a | OQ102885 |
| *Heterotrigona bakeri* (Cockerell, 1919) | NST CHU N5 | Chulabhorn District, Nakhon Si Thammarat, Thailand | NKC | 8°05′09″N, 99°52′46″E | Wild | outgroup | OR951994 | OQ102812 | OQ102891 |
| *Heterotrigona bakeri* (Cockerell, 1919) | NST CHU N13 | Chulabhorn District, Nakhon Si Thammarat, Thailand | NKC | 8°05′09″N, 99°52′46″E | Wild | outgroup | OR951995 | OQ102821 | OQ102901 |
| *Heterotrigona bakeri* (Cockerell, 1919) | NST CHU N17 | Chulabhorn District, Nakhon Si Thammarat, Thailand | NKC | 8°05′09″N, 99°52′46″E | Wild | outgroup | OR951996 | OQ102825 | OQ102904 |
| *Heterotrigona erythrogastra* (Cameron, 1902) | HE |  | - |  |  | outgroup | OR951997 | OR948590 | OR948598 |
| *Geniotrigona thoracica* (Smith, 1857) | GT |  | - |  |  | outgroup | OR951998 | OR948591 | OR948599 |
| *Homotrigona fimbriata* (Smith, 1857) | HF |  | - |  |  | outgroup | OR951999 | OR948592 | OR948600 |
| *Lophotrigona canifrons* (Smith, 1857) | LC |  | - |  |  | outgroup | OR952000 | OR948593 | OR948601 |
| *Lophotrigona canifrons* (Smith, 1857) | NST CHU N20 | Chulabhorn District, Nakhon Si Thammarat | - | 8°05′09″N, 99°52′46″E | Wild | outgroup | OR952001 | n/a | OQ102907 |
| *Tetragonilla collina* (Smith, 1857) | TC |  | - |  |  | outgroup | OR952002 | OR948594 | OR948602 |
| *Tetrigona melanoleuca* (Cockerell, 1929) | TM |  | - |  |  | outgroup | OR952003 | OR948595 | OR948603 |
