# Supplementary material for: Genetic structure of the commercial stingless bee Heterotrigona itama (Apidae: Meliponini) in Thailand
Source: PLoS One. 2024 Dec 4;19(12):e0312386. doi: 10.1371/journal.pone.0312386 (PMC11616864; doi:10.1371/journal.pone.0312386)
Supplement: S1 File — (DOCX) [file pone.0312386.s002.docx]

>HT01 NARACHAN1

GGGATTCTTTATATAATTTTTGCTATCTGGTCAGGCATTGCTGGTTCTTCATTTAGAATACTTATTCGAATAGAACTTAATAGACCTGGAACTTGAATTAGTAATGATCAGATCTACAACTCTATTGTAACTAGGCATGCATTTCTAATAATTTTCTTTATAGTAATGCCTTTTATAATTGGAGGGTTTGGAAACTTTCTAATTCCAATAATACTAGGATCTCCTGATATAGCTTTCCCCCGAATAAACAATATTAGGTTTTGACTATTACCCCCCTCACTTATACTGCTAATAACAAATAATTTATTTTTTCCCAATTCAGGAACTGGATGGACTGTGTACCCTCCTCTTTCCCTTTACATATACCATTCATCTCCTTCTGTAGACTTTACTATTTTTTCAATTCATATGACAGGAATTTCATCAATTCTTGGGTCACTTAACTTTATTGTAACAATTTTTATAATAAAGAATTTTTCTTTAAAGTACGATCAGATTAATCTTTTTTCATGATCTATCTCGATTACTGTGATTTTACTAATTATTTCTCTCCCAGTTCTAGCAGGAGCAATTACTATGCTGTTATTTGATCGAAATTTTAATACATCATTTTTTGATCCGATAGGAGGAGGAGATCCGATTCTTTATCAACATTTATTTATCTGCCCAATGAATTT-----TTTTAATGGCTGCAGTATAACTGACTGTACAAAGGTAGCAT---CAATTGGTTTT---ATGAAATCTGGAATGAAAGGATTAATGAAATATGTACTGTCTCAATTGTACAATTATGAAATTAAAATTTTAATAAAAATGTTAAAATTCACTTATGGGACGATAAGACCCTATAGAATTTTATATTGAAATTACTCAGTAGTTAATTCAGAAATAATTTCAATATTTGATTGGGAGGATTAT---ATT---TCAACTT--TAATT---GTTAACTTTAATTTAAGA-GTAAATAATGATCTTCAATTTGAAATTGCTAGAATAAATTACCTTAGGGATAACAGCGTAATACTTTTTTATAGGTCATATAGAAAAAAGTGGTTGCGACCTCGATGTTGAATTAGGATAAATTTTAAATGCAGGAGTTTAATAATTAAGTCTGTCGTCGACGGCGTAGGCTACCGTTGGTGCGCGATGCTCCGGATGGACCCCC-GCGGTTCCAT---CGAGGGCACGCCACCCTCGGATCGAACGCTCCTGCGTCGT---CGTGCACTTCTCCCCTAGTAGAACGTCGCGACCCGTTGTGTGTCGGTCTACGGCCCGAGCGGGAGACTGTCGCGTCGCTTCGGCGCACGCGGCAGACCCTCGGTCGCCCGGCCGGCTGCACGACGGTACACTCACGGTATCGGGCCGCAGCCAATCCATT--CTCGAATGTGTGTGCGTCCATCCCGCCGCAAGCTCGGTCAGTTCTTACCCGGAGGCCACGGACCCAGTGCCGTCCCCGGGCCTGGCCAGCTGTTAGCGGGCGGTGTCCTCGGACCGGCCAAACCTCGGATTACCGGTCGGCGACGCTATTGCTTTGGGTACTCTCAGGACCCGTCTTGAAACACGGACCAAGGAGTCTAACATGTGCGCGAGTCATTGGGATGCATAAACCTAAAGGCGAAATGAAAGTGAAAGTCGGCCTTCGCGCCGATCGAGGGAGGATGGGCCGCGTAACAATGCGGCCCCGCACTCCCGGGGCGTCTCGTTCTCACTGCGAGAAGAGGCG

>HT02 NARACHAN2

GGGATTCTTTATATAATTTTTGCTATCTGGTCAGGCATTGCTGGTTCTTCATTTAGAATACTTATCCGAATAGAGCTTAATAGACCTGGGACTTGAATTAGTAACGATCAGATCTACAACTCTATTGTAACTAGGCACGCATTTCTAATAATTTTCTTTATAGTAATGCCTTTTATAATTGGAGGGTTTGGAAACTTTCTAATTCCAATAATACTAGGATCTCCTGATATAGCTTTCCCCCGAATAAACAATATTAGGTTTTGACTATTACCTCCTTCACTTATACTTCTAATAACAAATAATTTATTTTTTCCCAATTCAGGAACTGGATGGACTGTGTACCCTCCTCTTTCCCTTTACATATACCATTCATCCCCTTCTGTAGACTTTACTATTTTTTCAATTCATATGACAGGAATTTCATCAATTCTTGGGTCACTTAACTTTATTGTAACAATTTTTATAATAAAGAATTTTTCTTTAAAGTACGATCAGATTAATCTTTTTTCATGATCTATCTCGATTACTGTGATTTTACTAATTATTTCTCTCCCAGTTCTAGCTGGAGCAATTACTATGCTGTTATTTGATCGAAATTTTAATACATCATTTTTTGATCCGATAGGAGGAGGAGATCCGATTCTTTATCAACATTTATTTATCTGCCCAATGAATTT-----TTTTAATGGCTGCAGTATAACTGACTGTACAAAGGTAGCAT---CAATTGGTTTT---ATGAAATCTGGAATGAAAGGATTAATGAAATATGTACTGTCTCAATTGTACAATTATGAAATTAAAATTTTAATAAAAATGTTAAAATTCACTTATGGGACGATAAGACCCTATAGAATTTTATATTGAAATTACTCAGTAGTTAATTCAGAAATAATTTCAATATTTGATTGGGAGGATTAT---ATT---TCAACTT--TAATT---GTTAACTTTAATTTAAGA-GTAAATAATGATCTTCAATTTGAAATTGCTAGAATAAATTACCTTAGGGATAACAGCGTAATACTTTTTTATAGGTCATATAGAAAAAAGTGGTTGCGACCTCGATGTTGAATTAGGATAAATTTTAAATGCAGGAGTTTAATAATTAAGTCTGTCGTCGACGGCGTAGGCTACCGTTGGTGCGCGATGCTCCGGATGGACCCCC-GCGGTTCCAT---CGAGGGCACGCCACCCTCGGATCGAACGCTCCTGCGTCGT---CGTGCACTTCTCCCCTAGTAGAACGTCGCGACCCGTTGTGTGTCGGTCTACGGCCCGAGCGGGAGACTGTCGCGTCGCTTCGGCGCACGCGGCAGACCCTCGGTCGCCCGGCCGGCTGCACGACGGTACACTCACGGTATCGGGCCGCAGCCAATCCATT--CTCGAATGTGTGTGCGTCCATCCCGCCGCAAGCTCGGTCAGTTCTTACCCGGAGGCCACGGACCCAGTGCCGTCCCCGGGCCTGGCCAGCTGTTAGCGGGCGGTGTCCTCGGACCGGCCAAACCTCGGATTACCGGTCGGCGACGCTATTGCTTTGGGTACTCTCAGGACCCGTCTTGAAACACGGACCAAGGAGTCTAACATGTGCGCGAGTCATTGGGATGCATAAACCTAAAGGCGAAATGAAAGTGAAAGTCGGCCTTCGCGCCGATCGAGGGAGGATGGGCCGCGTAACAATGCGGCCCCGCACTCCCGGGGCGTCTCGTTCTCACTGCGAGAAGAGGCG

>HT03 NARACHAN3

GGGATTCTTTATATAATTTTTGCTATCTGGTCAGGCATTGCTGGTTCTTCATTTAGAATACTTATCCGAATAGAGCTTAATAGACCTGGGACTTGAATTAGTAACGATCAGATCTACAACTCTATTGTAACTAGGCACGCATTTCTAATAATTTTCTTTATAGTAATGCCTTTTATAATTGGAGGGTTTGGAAACTTTCTAATTCCAATAATACTAGGATCTCCTGATATAGCTTTCCCCCGAATAAACAATATTAGGTTTTGACTATTACCTCCTTCACTTATACTTCTAATAACAAATAATTTATTTTTTCCCAATTCAGGAACTGGATGGACTGTGTACCCTCCTCTTTCCCTTTACATATACCATTCATCTCCTTCTGTAGACTTTACTATTTTTTCAATTCATATGACAGGAATTTCATCAATTCTTGGGTCACTTAACTTTATTGTAACAATTTTTATAATAAAGAATTTTTCTTTAAAGTACGATCAGATTAATCTTTTTTCATGATCTATCTCGATTACTGTGATTTTACTAATTATTTCTCTCCCAGTTCTAGCTGGAGCAATTACTATGCTGTTATTTGATCGAAATTTTAATACATCATTTTTTGATCCGATAGGAGGAGGAGATCCGATTCTTTATCAACATTTATTTATCTGCCCAATGAATTT-----TTTTAATGGCTGCAGTATAACTGACTGTACAAAGGTAGCAT---CAATTGGTTTT---ATGAAATCTGGAATGAAAGGATTAATGAAATATGTACTGTCTCAATTGTACAATTATGAAATTAAAATTTTAATAAAAATGTTAAAATTCACTTATGGGACGATAAGACCCTATAGAATTTTATATTGAAATTACTCAGTAGTTAATTCAGAAATAATTTCAATATTTGATTGGGAGGATTAT---ATT---TCAACTT--TAATT---GTTAACTTTAATTTAAGA-GTAAATAATGATCTTCAATTTGAAATTGCTAGAATAAATTACCTTAGGGATAACAGCGTAATACTTTTTTATAGGTCATATAGAAAAAAGTGGTTGCGACCTCGATGTTGAATTAGGATAAATTTTAAATGCAGGAGTTTAATAATTAAGTCTGTCGTCGACGGCGTAGGCTACCGTTGGTGCGCGATGCTCCGGATGGACCCCC-GCGGTTCCAT---CGAGGGCACGCCACCCTCGGATCGAACGCTCCTGCGTCGT---CGTGCACTTCTCCCCTAGTAGAACGTCGCGACCCGTTGTGTGTCGGTCTACGGCCCGAGCGGGAGACTGTCGCGTCGCTTCGGCGCACGCGGCAGACCCTCGGTCGCCCGGCCGGCTGCACGACGGTACACTCACGGTATCGGGCCGCAGCCAATCCATT--CTCGAATGTGTGTGCGTCCATCCCGCCGCAAGCTCGGTCAGTTCTTACCCGGAGGCCACGGACCCAGTGCCGTCCCCGGGCCTGGCCAGCTGTTAGCGGGCGGTGTCCTCGGACCGGCCAAACCTCGGATTACCGGTCGGCGACGCTATTGCTTTGGGTACTCTCAGGACCCGTCTTGAAACACGGACCAAGGAGTCTAACATGTGCGCGAGTCATTGGGATGCATAAACCTAAAGGCGAAATGAAAGTGAAAGTCGGCCTTCGCGCCGATCGAGGGAGGATGGGCCGCGTAACAATGCGGCCCCGCACTCCCGGGGCGTCTCGTTCTCACTGCGAGAAGAGGCG

>HT06 NARAYNC1

GGGATTCTTTATATAATTTTTGCTATCTGGTCAGGCATTGCTGGTTCTTCATTTAGAATACTTATCCGAATAGAGCTTAATAGACCTGGGACTTGAATTAGTAACGATCAGATCTACAACTCTATTGTAACTAGGCACGCATTTCTAATAATTTTCTTTATAGTAATGCCTTTTATAATTGGAGGGTTTGGAAACTTTCTAATTCCAATAATACTAGGATCTCCTGATATAGCTTTCCCCCGAATAAACAATATTAGGTTTTGACTATTACCTCCTTCACTTATACTTCTAATAACAAATAATTTATTTTTTCCCAATTCAGGAACTGGATGGACTGTGTACCCTCCTCTTTCCCTTTACATATACCATTCATCCCCTTCTGTAGACTTTACTATTTTTTCAATTCATATGACAGGAATTTCATCAATTCTTGGGTCACTTAACTTTATTGTAACAATTTTTATAATAAAGAATTTTTCTTTAAAGTACGATCAGATTAATCTTTTTTCATGATCTATCTCGATTACTGTGATTTTACTAATTATTTCTCTCCCAGTTCTAGCTGGAGCAATTACTATGCTGTTATTTGATCGAAATTTTAATACATCATTTTTTGATCCGATAGGAGGAGGAGATCCGATTCTTTATCAACATTTATTTATCTGCCCAATGAATTT-----TTTTATAGGCTGCAGTATAACTGACTGTACAAAGGTAGCAT---CAATTGGTTTT---ATGAAATCTGGAATGAAAGGATTAATGAAATATGTACTGTCTCAATTGTACAATTATGAAATTAAAATTTTAATAAAAATGTTAAAATTCACTTATGGGACGATAAGACCCTATAGAATTTTATATTGAAATTACTCAGTAGTTAATTCAGAAATAATTTCAATATTTGATTGGGAGGATTAT---ATT---TCAACTT--TAATT---GTTAACTTTAATTTAAGA-GTAAATAATGATCTTCAATTTGAAATTGCTAGAATAAATTACCTTAGGGATAACAGCGTAATACTTTTTTATAGGTCATATAGAAAAAAGTGGTTGCGACCTCGATGTTGAATTAGGATAAATTTTAAATGCAGGAGTTTAATAATTAAGTCTGTCGTCGACGGCGTAGGCTACCGTTGGTGCGCGATGCTCCGGATGGACCCCC-GCGGTTCCAT---CGAGGGCACGCCACCCTCGGATCGAACGCTCCTGCGTCGT---CGTGCACTTCTCCCCTAGTAGAACGTCGCGACCCGTTGTGTGTCGGTCTACGGCCCGAGCGGGAGACTGTCGCGTCGCTTCGGCGCACGCGGCAGACCCTCGGTCGCCCGGCCGGCTGCACGACGGTACACTCACGGTATCGGGCCGCAGCCAATCCATT--CTCGAATGTGTGTGCGTCCATCCCGCCGCAAGCTCGGTCAGTTCTTACCCGGAGGCCACGGACCCAGTGCCGTCCCCGGGCCTGGCCAGCTGTTAGCGGGCGGTGTCCTCGGACCGGCCAAACCTCGGATTACCGGTCGGCGACGCTATTGCTTTGGGTACTCTCAGGACCCGTCTTGAAACACGGACCAAGGAGTCTAACATGTGCGCGAGTCATTGGGATGCATAAACCTAAAGGCGAAATGAAAGTGAAAGTCGGCCTTCGCGCCGATCGAGGGAGGATGGGCCGCGTAACAATGCGGCCCCGCACTCCCGGGGCGTCTCGTTCTCACTGCGAGAAGAGGCG

>HT07 NARAYNC2

GGGATTCTTTATATAATTTTTGCTATCTGGTCAGGCATTGCTGGTTCTTCATTTAGAATACTTATCCGAATAGAGCTTAATAGACCTGGGACTTGAATTAGTAACGATCAGATCTACAACTCTATTGTAACTAGGCACGCATTTCTAATAATTTTCTTTATAGTAATGCCTTTTATAATTGGAGGGTTTGGAAACTTTCTAATTCCAATAATACTAGGATCTCCTGATATAGCTTTCCCCCGAATAAACAATATTAGGTTTTGACTATTACCTCCTTCACTTATACTTCTAATAACAAATAATTTATTTTTTCCCAATTCAGGAACTGGATGGACTGTGTACCCTCCTCTTTCCCTTTACATATACCATTCATCTCCTTCTGTAGACTTTACTATTTTTTCAATTCATATGACAGGAATTTCATCAATTCTTGGGTCACTTAACTTTATTGTAACAATTTTTATAATAAAGAATTTTTCTTTAAAGTACGATCAGATTAATCTTTTTTCATGATCTATCTCGATTACTGTGATTTTACTAATTATTTCTCTCCCAGTTCTAGCTGGAGCAATTACTATGCTGTTATTTGATCGAAATTTTAATACATCATTTTTTGATCCGATAGGAGGAGGAGATCCGATTCTTTATCAACATTTATTTATCTGCCCAATGAATTT-----TTTTAATGGCTGCAGTATAACTGACTGTACAAAGGTAGCAT---CAATTGGTTTT---ATGAAATCTGGAATGAAAGGATTAATGAAATATGTACTGTCTCAATTGTACAATTATGAAATTAAAATTTTAATAAAAATGTTAAAATTCACTTATGGGACGATAAGACCCTATAGAATTTTATATTGAAATTACTCAGTAGTTAATTCAGAAATAATTTCAATATTTGATTGGGAGGATTAT---ATT---TCAACTT--TAATT---GTTAACTTTAATTTAAGA-GTAAATAATGATCTTCAATTTGAAATTGCTAGAATAAATTACCTTAGGGATAACAGCGTAATACTTTTTTATAGGTCATATAGAAAAAAGTGGTTGCGACCTCGATGTTGAATTAGGATAAATTTTAAATGCAGGAGTTTAATAATTAAGTCTGTCGTCGACGGCGTAGGCTACCGTTGGTGCGCGATGCTCCGGATGGACCCCC-GCGGTTCCAT---CGAGGGCACGCCACCCTCGGATCGAACGCTCCTGCGTCGT---CGTGCACTTCTCCCCTAGTAGAACGTCGCGACCCGTTGTGTGTCGGTCTACGGCCCGAGCGGGAGACTGTCGCGTCGCTTCGGCGCACGCGGCAGACCCTCGGTCGCCCGGCCGGCTGCACGACGGTACACTCACGGTATCGGGCCGCAGCCAATCCATT--CTCGAATGTGTGTGCGTCCATCCCGCCGCAAGCTCGGTCAGTTCTTACCCGGAGGCCACGGACCCAGTGCCGTCCCCGGGCCTGGCCAGCTGTTAGCGGGCGGTGTCCTCGGACCGGCCAAACCTCGGATTACCGGTCGGCGACGCTATTGCTTTGGGTACTCTCAGGACCCGTCTTGAAACACGGACCAAGGAGTCTAACATGTGCGCGAGTCATTGGGATGCATAAACCTAAAGGCGAAATGAAAGTGAAAGTCGGCCTTCGCGCCGATCGAGGGAGGATGGGCCGCGTAACAATGCGGCCCCGCACTCCCGGGGCGTCTCGTTCTCACTGCGAGAAGAGGCG

>HT08 NARAYNC3

GGGATTCTTTATATAATTTTTGCTATCTGGTCAGGCATTGCTGGTTCTTCATTTAGAATACTTATCCGAATAGAGCTTAATAGACCTGGGACTTGAATTAGTAACGATCAGATCTACAACTCTATTGTAACTAGGCACGCATTTCTAATAATTTTCTTTATAGTAATGCCTTTTATAATTGGAGGGTTTGGAAACTTTCTAATTCCAATAATACTAGGATCTCCTGATATAGCTTTCCCCCGAATAAACAATATTAGGTTTTGACTATTACCTCCTTCACTTATACTTCTAATAACAAATAATTTATTTTTTCCCAATTCAGGAACTGGATGGACTGTGTACCCTCCTCTTTCCCTTTACATATACCATTCATCTCCTTCTGTAGACTTTACTATTTTTTCAATTCATATGACAGGAATTTCATCAATTCTTGGGTCACTTAACTTTATTGTAACAATTTTTATAATAAAGAATTTTTCTTTAAAGTACGATCAGATTAATCTTTTTTCATGATCTATCTCGATTACTGTGATTTTACTAATTATTTCTCTCCCAGTTCTAGCTGGAGCAATTACTATGCTGTTATTTGATCGAAATTTTAATACATCATTTTTTGATCCGATAGGAGGAGGAGATCCGATTCTTTATCAACATTTATTTATCTGCCCAATGAATTT-----TTTTAATGGCTGCAGTATAACTGACTGTACAAAGGTAGCAT---CAATTGGTTTT---ATGAAATCTGGAATGAAAGGATTAATGAAATATGTACTGTCTCAATTGTACAATTATGAAATTAAAATTTTAATAAAAATGTTAAAATTCACTTATGGGACGATAAGACCCTATAGAATTTTATATTGAAATTACTCAGTAGTTAATTCAGAAATAATTTCAATATTTGATTGGGAGGATTAT---ATT---TCAACTT--TAATT---GTTAACTTTAATTTAAGA-GTAAATAATGATCTTCAATTTGAAATTGCTAGAATAAATTACCTTAGGGATAACAGCGTAATACTTTTTTATAGGTCATATAGAAAAAAGTGGTTGCGACCTCGATGTTGAATTAGGATAAATTTTAAATGCAGGAGTTTAATAATTAAGTCTGTCGTCGACGGCGTAGGCTACCGTTGGTGCGCGATGCTCCGGATGGACCCCC-GCGGTTCCAT---CGAGGGCACGCCACCCTCGGATCGAACGCTCCTGCGTCGT---CGTGCACTTCTCCCCTAGTAGAACGTCGCGACCCGTTGTGTGTCGGTCTACGGCCCGAGCGGGAGGCTGTCGCGTCGCTTCGGCGCACGCGGCAGACCCTCGGTCGCCCGGCCGGCTGCACGACGGTACACTCACGGTATCGGGCCGCAGCCAATCCATT--CTCGAATGTGTGTGCGTCCATCCCGCCGCAAGCTCGGTCAGTTCTTACCCGGAGGCCACGGACCCAGTGCCGTCCCCGGGCCTGGCCAGCTGTTAGCGGGCGGTGTCCTCGGACCGGCCAAACCTCGGATTACCGGTCGGCGACGCTATTGCTTTGGGTACTCTCAGGACCCGTCTTGAAACACGGACCAAGGAGTCTAACATGTGCGCGAGTCATTGGGATGCATAAACCTAAAGGCGAAATGAAAGTGAAAGTCGGCCTTCGCGCCGATCGAGGGAGGATGGGCCGCGTAACAATGCGGCCCCGCACTCCCGGGGCGTCTCGTTCTCACTGCGAGAAGAGGCG

>HT09 NARAYNC4

GGGATTCTTTATATAATTTTTGCTATCTGGTCAGGCATTGCTGGTTCTTCATTTAGAATACTTATCCGAATAGAGCTTAATAGACCTGGGACTTGAATTAGTAACGATCAGATCTACAACTCTATTGTAACTAGGCACGCATTTCTAATAATTTTCTTTATAGTAATGCCTTTTATAATTGGAGGGTTTGGAAACTTTCTAATTCCAATAATACTAGGATCTCCTGATATAGCTTTCCCCCGAATAAACAATATTAGGTTTTGACTATTACCTCCTTCACTTATACTTCTAATAACAAATAATTTATTTTTTCCCAATTCAGGAACTGGATGGACTGTGTACCCTCCTCTTTCCCTTTACATATACCATTCATCCCCTTCTGTAGACTTTACTATTTTTTCAATTCATATGACAGGAATTTCATCAATTCTTGGGTCACTTAACTTTATTGTAACAATTTTTATAATAAAGAATTTTTCTTTAAAGTACGATCAGATTAATCTTTTTTCATGATCTATCTCGATTACTGTGATTTTACTAATTATTTCTCTCCCAGTTCTAGCTGGAGCAATTACTATGCTGTTATTTGATCGAAATTTTAATACATCATTTTTTGATCCGATAGGAGGAGGAGATCCGATTCTTTATCAACATTTATTTATCTGCCCAATGAATTT-----TTTTAATGGCTGCAGTATAACTGACTGTACAAAGGTAGCAT---CAATTGGTTTT---ATGAAATCTGGAATGAAAGGATTAATGAAATATGTACTGTCTCAATTGTACAATTATGAAATTAAAATTTTAATAAAAATGTTAAAATTCACTTATGGGACGATAAGACCCTATAGAATTTTATATTGAAATTACTCAGTAGTTAATTCAGAAATAATTTCAATATTTGATTGGGAGGATTAT---ATT---TCAACTT--TAATT---GTTAACTTTAATTTAAGA-GTAAATAATGATCTTCAATTTGAAATTGCTAGAATAAATTACCTTAGGGATAACAGCGTAATACTTTTTTATAGGTCATATAGAAAAAAGTGGTTGCGACCTCGATGTTGAATTAGGATAAATTTTAAATGCAGGAGTTTAATAATTAAGTCTGTCGTCGACGGCGTAGGCTACCGTTGGTGCGCGATGCTCCGGATGGACCCCC-GCGGTTCCAT---CGAGGGCACGCCACCCTCGGATCGAACGCTCCTGCGTCGT---CGTGCACTTCTCCCCTAGTAGAACGTCGCGACCCGTTGTGTGTCGGTCTACGGCCCGAGCGGGAGACTGTCGCGTCGCTTCGGCGCACGCGGCAGACCCTCGGTCGCCCGGCCGGCTGCACGACGGTACACTCACGGTATCGGGCCGCAGCCAATCCATT--CTCGAATGTGTGTGCGTCCATCCCGCCGCAAGCTCGGTCAGTTCTTACCCGGAGGCCACGGACCCAGTGCCGTCCCCGGGCCTGGCCAGCTGTTAGCGGGCGGTGTCCTCGGACCGGCCAAACCTCGGATTACCGGTCGGCGACGCTATTGCTTTGGGTACTCTCAGGACCCGTCTTGAAACACGGACCAAGGAGTCTAACATGTGCGCGAGTCATTGGGATGCATAAACCTAAAGGCGAAATGAAAGTGAAAGTCGGCCTTCGCGCCGATCGAGGGAGGATGGGCCGCGTAACAATGCGGCCCCGCACTCCCGGGGCGTCTCGTTCTCACTGCGAGAAGAGGCG

>HT11 NARAYNC6

GGGATTCTTTATATAATTTTTGCTATCTGGTCAGGCATTGCTGGTTCTTCATTTAGAATACTTATCCGAATAGAGCTTAATAGACCTGGGACTTGAATTAGTAACGATCAGATCTACAACTCTATTGTAACTAGGCACGCATTTCTAATAATTTTCTTTATAGTAATGCCTTTTATAATTGGAGGGTTTGGAAACTTTCTAATTCCAATAATACTAGGATCTCCTGATATAGCTTTCCCCCGAATAAACAATATTAGGTTTTGACTATTACCTCCTTCACTTATACTTCTAATAACAAATAATTTATTTTTTCCCAATTCAGGAACTGGATGGACTGTGTACCCTCCTCTTTCCCTTTACATATACCATTCATCTCCTTCTGTAGACTTTACTATTTTTTCAATTCATATGACAGGAATTTCATCAATTCTTGGGTCACTTAACTTTATTGTAACAATTTTTATAATAAAGAATTTTTCTTTAAAGTACGATCAGATTAATCTTTTTTCATGATCTATCTCGATTACTGTGATTTTACTAATTATTTCTCTCCCAGTTCTAGCTGGAGCAATTACTATGCTGTTATTTGATCGAAATTTTAATACATCATTTTTTGATCCGATAGGAGGAGGAGATCCGATTCTTTATCAACATTTATTTATCTGCCCAATGAATTT-----TTTTAATGGCTGCAGTATAACTGACTGTACAAAGGTAGCAT---CAATTGGTTTT---ATGAAATCTGGAATGAAAGGATTAATGAAATATGTACTGTCTCAATTGTACAATTATGAAATTAAAATTTTAATAAAAATGTTAAAATTCACTTATGGGACGATAAGACCCTATAGAATTTTATATTGAAATTACTCAGTAGTTAATTCAGAAATAATTTCAATATTTGATTGGGAGGATTAT---ATT---TCAACTT--TAATT---GTTAACTTTAATTTAAGA-GTAAATAATGATCTTCAATTTGAAATTGCTAGAATAAATTACCTTAGGGATAACAGCGTAATACTTTTTTATAGGTCATATAGAAAAAAGTGGTTGCGACCTCGATGTTGAATTAGGATAAATTTTAAATGCAGGAGTTTAATAATTAAGTCTGTCGTCGACGGCGTAGGCTACCGTTGGTGCGCGATGCTCCGGATGGACCCCC-GCGGTTCCAT---CGAGGGCACGCCACCCTCGGATCGAACGCTCCTGCGTCGT---CGTGCACTTCTCCCCTAGTAGAACGTCGCGACCCGTTGTGTGTCGGTCTACGGCCCGAGCGGGAGGCTGTCGCGTCGCTTCGGCGCACGCGGCAGACCCTCGGTCGCCCGGCCGGCTGCACGACGGTACACTCACGGTATCGGGCCGCAGCCAATCCATT--CTCGAATGTGTGTGCGTCCATCCCGCCGCAAGCTCGGTCAGTTCTTACCCGGAGGCCACGGACCCAGTGCCGTCCCCGGGCCTGGCCAGCTGTTAGCGGGCGGTGTCCTCGGACCGGCCAAACCTCGGATTACCGGTCGGCGACGCTATTGCTTTGGGTACTCTCAGGACCCGTCTTGAAACACGGACCAAGGAGTCTAACATGTGCGCGAGTCATTGGGATGCATAAACCTAAAGGCGAAATGAAAGTGAAAGTCGGCCTTCGCGCCGATCGAGGGAGGATGGGCCGCGTAACAATGCGGCCCCGCACTCCCGGGGCGTCTCGTTCTCACTGCGAGAAGAGGCG

>HT12 NARATBC7

GGGATTCTGTATATAATTTTTGCTATCTGATCAGGTATTGCTGGTTCTTCCTTCAGAATACTTATTCGAATGGAACTTAATAGCCCTGGAACTTGAATTAGTAATGATCAGATCTACAACTCTATTGTAACTAGGCATGCATTTCTGATAATTTTTTTTATAGTAATACCTTTTATAATTGGAGGGTTTGGAAATTTTTTGATTCCACTAATACTAGGATCTCCTGATATAGCTTTTCCTCGAATAAACAATATTAGCTTTTGACTTCTACCTCCTTCACTTATACTGCTAATAACAAATAATTTATTTTTTCCAAATTCAGGAACTGGATGAACTGTGTACCCTCCTCTTTCTCTCTACATATACCATTCATCCCCTTCAGTAGACTTTACTATTTTTTCAATCCATATAACAGGAATTTCATCAATTCTTGGATCACTTAACTTTATTGTAACAATTTTTATAATAAAGAATTTTTCTTTAAAGTACGATCAAATTAATCTTTTTTCATGATCTATCTCAATTACTGTAATTTTACTAATCCTTTCTCTCCCAGTTCTAGCGGGAGCAATTACTATGCTGTTATTTGACCGAAATTTTAACACTTCCTTCTTTGATCCAATAGGAGGGGGAGATCCAATTCTTTATCAACATCTATTTATCTGCCCAATGAATTT-----TTTTAATGGCTGCAGTATAACTGACTGTACAAAGGTAGCAT---CAATTGGTTTT---ATGAAATCTGGAATGAAAGGATTAATGAAATATGTACTGTCTCAATTGTACAATTATGAAATTAAAATTTTAATAAAAATGTTAAAATTCACTTATGGGACGATAAGACCCTATAGAATTTTATATTGAAATTACTCAGTAGTTAATTCAGAAATAGTTTCAATATTTGATTGGGAGGATTAT---AAT---CCAACTT--TAATT---GTTAACTTTAATTTAAGA-GTAGATAATGATCTTCAATTTGAAATTGCTAGAATAAATTACCTTAGGGATAACAGCGTAATACTTTTTTATAGGCCATATAGAAAAAAGTGGTTGCGACCTCGATGTTGAATTAGGATAAATTTTAAATGCAGGAGTTTAATAATTAAGTCTGTCGTCGACGGCGTAGGCTACCGTTGGTGCGCGATGCTCCGGATGGACCCCC-GCGGTTCCAT---CGAGGGCACGCCACCCTCGGATCGAACGCTCCTGCGTCGT---CGTGCACTTCTCCCCTAGTAGAACGTCGCGACCCGTTGTGTGTCGGTCTACGGCCCGAGCGGGAGACTGTCGCGTCGCTTCGGCGCACGCGGCAGACCCTCGGTCGCCCGGCCGGCTGCACGACGGTACACTCACGGTATCGGGCCGCAGCCAATCCATT--CTCGAATGTGTGTGCGTCCATCCCGCCGCAAGCTCGGTCAGTTCTTACCCGGAGGCCACGGACCCAGTGCCGTCCCCGGGCCTGGCCAGCTGTTAGCGGGCGGTGTCCTCGGACCGGCCAAACCTCGGATTACCGGTCGGCGACGCTATTGCTTTGGGTACTCTCAGGACCCGTCTTGAAACACGGACCAAGGAGTCTAACATGTGCGCGAGTCATTGGGATGCATAAACCTAAAGGCGAAATGAAAGTGAAAGTCGGCCTTCGCGCCGATCGAGGGAGGATGGGCCGCGTAACAATGCGGCCCCGCACTCCCGGGGCGTCTCGTTCTCACTGCGAGAAGAGGCG

>HT14 NARATBC9

GGGATTCTGTATATAATTTTTGCTATCTGATCAGGTATTGCTGGTTCTTCCTTCAGAATACTTATTCGAATGGAACTTAATAGCCCTGGAACTTGAATTAGTAATGATCAGATCTACAACTCTATTGTAACTAGGCATGCATTTCTGATAATTTTCTTTATAGTAATACCTTTTATAATTGGAGGGTTTGGAAATTTTTTGATTCCACTAATACTAGGATCTCCTGATATAGCTTTTCCTCGAATAAACAATATTAGCTTTTGACTTCTACCTCCTTCACTTATACTGCTAATAACAAATAATTTATTTTTTCCAAATTCAGGAACTGGATGAACTGTGTACCCTCCTCTTTCTCTCTACATATACCATTCATCCCCTTCAGTAGACTTTACCATTTTTTCAATCCATATAACAGGGATTTCATCAATTCTTGGATCACTTAACTTTATTGTAACAATTTTTATAATAAAGAATTTTTCTCTAAAGTACGATCAAATTAATCTTTTTTCATGATCTATCTCAATTACTGTAATTTTACTAATCCTTTCTCTCCCAGTTCTAGCGGGAGCAATTACTATGCTGTTATTTGACCGAAATTTTAACACCTCCTTCTTTGATCCAATAGGAGGGGGAGATCCAATTCTTTATCAACATCTATTTATCTGCCCAATGAATTT-----TTTTAATGGCTGCAGTATAACTGACTGTACAAAGGTAGCAT---CAATTGGTTTT---ATGAAATCTGGAATGAAAGGATTAATGAAATATGTACTGTCTCAATTGTACAATTATGAAATTAAAATTTTAATAAAAATGTTAAAATTCACTTATGGGACGATAAGACCCTATAGAATTTTATATTGAAATTACTCAGTAGTTAATTCAGAAATAGTTTCAATATTTGATTGGGAGGATTAT---AAT---CCAACTT--TAATT---GTTAACTTTAATTTAAGA-GTAGATAATGATCTTCAATTTGAAATTGCTAGAATAAATTACCTTAGGGATAACAGCGTAATACTTTTTTATAGGCCATATAGAAAAAAGTGGTTGCGACCTCGATGTTGAATTAGGATAAATTTTAAATGCAGGAGTTTAATAATTAAGTCTGTCGTCGACGGCGTAGGCTACCGTTGGTGCGCGATGCTCCGGATGGACCCCC-GCGGTTCCAT---CGAGGGCACGCCACCCTCGGATCGAACGCTCCTGCGTCGT---CGTGCACTTCTCCCCTAGTAGAACGTCGCGACCCGTTGTGTGTCGGTCTACGGCCCGAGCGGGAGACTGTCGCGTCGCTTCGGCGCACGCGGCAGACCCTCGGTCGCCCGGCCGGCTGCACGACGGTACACTCACGGTATCGGGCCGCAGCCAATCCATT--CTCGAATGTGTGTGCGTCCATCCCGCCGCAAGCTCGGTCAGTTCTTACCCGGAGGCCACGGACCCAGTGCCGTCCCCGGGCCTGGCCAGCTGTTAGCGGGCGGTGTCCTCGGACCGGCCAAACCTCGGATTACCGGTCGGCGACGCTATTGCTTTGGGTACTCTCAGGACCCGTCTTGAAACACGGACCAAGGAGTCTAACATGTGCGCGAGTCATTGGGATGCATAAACCTAAAGGCGAAATGAAAGTGAAAGTCGGCCTTCGCGCCGATCGAGGGAGGATGGGCCGCGTAACAATGCGGCCCCGCACTCCCGGGGCGTCTCGTTCTCACTGCGAGAAGAGGCG

>HT15 NARATBC10

GGGATTCTGTATATAATTTTTGCTATCTGATCAGGTATTGCTGGTTCTTCCTTCAGAATACTTATTCGAATGGAACTTAATAGCCCTGGAACTTGAATTAGTAATGATCAGATCTACAACTCTATTGTAACTAGGCATGCATTTCTGATAATTTTTTTTATAGTAATACCTTTTATGATTGGAGGGTTTGGAAATTTTTTGATTCCACTAATACTAGGATCTCCTGATATAGCTTTTCCTCGAATAAACAATATTAGCTTTTGACTTCTACCTCCTTCACTTATACTGCTAATAACAAATAATTTATTTTTTCCAAATTCAGGAACTGGATGAACTGTGTACCCTCCTCTTTCTCTCTACATATACCATTCATCCCCTTCAGTAGACTTTACTATTTTTTCAATCCATATAACAGGGATTTCATCAATTCTTGGATCACTTAACTTTATTGTAACAATTTTTATAATAAAGAATTTTTCTTTAAAGTACGATCAAATTAATCTTTTTTCATGATCTATCTCAATTACTGTAATTTTACTAATTCTTTCTCTTCCAGTTCTAGCGGGAGCAATTACTATGCTGTTATTTGACCGAAATTTTAACACCTCCTTCTTTGATCCAATAGGAGGGGGAGATCCAATTCTTTATCAACATCTATTTATCTGCCCAATGAATTT-----TTTTAATGGCTGCAGTATAACTGACTGTACAAAGGTAGCAT---CAATTGGTTTT---ATGAAATCTGGAATGAAAGGATTAATGAAATATGTACTGTCTCAATTGTACAATTATGAAATTAAAATTTTAATAAAAATGTTAAAATTCACTTATGGGACGATAAGACCCTATAGAATTTTATATTGAAATTACTCAGTAGTTAATTCAGAAATAGTTTCAATATTTGATTGGGAGGATTAT---AAT---CCAACTT--TAATT---GTTAACTTTAATTTAAGA-GTAGATAATGATCTTCAATTTGAAATTGCTAGAATAAATTACCTTAGGGATAACAGCGTAATACTTTTTTATAGGCCATATAGAAAAAAGTGGTTGCGACCTCGATGTTGAATTAGGATAAATTTTAAATGCAGGAGTTTAATAATTAAGTCTGTCGTCGACGGCGTAGGCTACCGTTGGTGCGCGATGCTCCGGATGGACCCCC-GCGGTTCCAT---CGAGGGCACGCCACCCTCGGATCGAACGCTCCTGCGTCGT---CGTGCACTTCTCCCCTAGTAGAACGTCGCGACCCGTTGTGTGTCGGTCTACGGCCCGAGCGGGAGACTGTCGCGTCGCTTCGGCGCACGCGGCAGACCCTCGGTCGCCCGGCCGGCTGCACGACGGTACACTCACGGTATCGGGCCGCAGCCAATCCATT--CTCGAATGTGTGTGCGTCCATCCCGCCGCAAGCTCGGTCAGTTCTTACCCGGAGGCCACGGACCCAGTGCCGTCCCCGGGCCTGGCCAGCTGTTAGCGGGCGGTGTCCTCGGACCGGCCAAACCTCGGATTACCGGTCGGCGACGCTATTGCTTTGGGTACTCTCAGGACCCGTCTTGAAACACGGACCAAGGAGTCTAACATGTGCGCGAGTCATTGGGATGCATAAACCTAAAGGCGAAATGAAAGTGAAAGTCGGCCTTCGCGCCGATCGAGGGAGGATGGGCCGCGTAACAATGCGGCCCCGCACTCCCGGGGCGTCTCGTTCTCACTGCGAGAAGAGGCG

>HT16 NARATBC11

GGGATTCTGTATATAATTTTTGCTATCTGATCAGGTATTGCTGGTTCTTCCTTCAGAATACTTATTCGAATGGAACTTAATAGCCCTGGAACTTGAATTAGTAATGATCAGATCTACAACTCTATTGTAACTAGGCATGCATTTCTGATAATTTTCTTTATAGTAATACCTTTTATAATTGGAGGGTTTGGAAATTTTTTGATTCCACTAATACTAGGATCTCCTGATATAGCTTTTCCTCGAATAAACAATATTAGCTTTTGACTTCTACCTCCTTCACTTATACTGCTAATAACAAATAATTTATTTTTTCCAAATTCAGGAACTGGATGAACTGTGTACCCTCCTCTTTCTCTCTACATATACCATTCATCCCCTTCAGTAGACTTTACCATTTTTTCAATCCATATAACAGGGATTTCATCAATTCTTGGATCACTTAACTTTATTGTAACAATTTTTATAATAAAGAATTTTTCTCTAAAGTACGATCAAATTAATCTTTTTTCATGATCTATCTCAATTACTGTAATTTTACTAATCCTTTCTCTCCCAGTTCTAGCGGGAGCAATTACTATGCTGTTATTTGACCGAAATTTTAACACCTCCTTCTTTGATCCAATAGGAGGGGGAGATCCAATTCTTTATCAACATCTATTTATCTGCCCAATGAATTT-----TTTTAATGGCTGCAGTATAACTGACTGTACAAAGGTAGCAT---CAATTGGTTTT---ATGAAATCTGGAATGAAAGGATTAATGAAATATGTACTGTCTCAATTGTACAATTATGAAATTAAAATTTTAATAAAAATGTTAAAATTCACTTATGGGACGATAAGACCCTATAGAATTTTATATTGAAATTACTCAGTAGTTAATTCAGAAATAGTTTCAATATTTGATTGGGAGGATTAT---AAT---CCAACTT--TAATT---GTTAACTTTAATTTAAGA-GTAGATAATGATCTTCAATTTGAAATTGCTAGAATAAATTACCTTAGGGATAACAGCGTAATACTTTTTTATAGGCCATATAGAAAAAAGTGGTTGCGACCTCGATGTTGAATTAGGATAAATTTTAAATGCAGGAGTTTAATAATTAAGTCTGTCGTCGACGGCGTAGGCTACCGTTGGTGCGCGATGCTCCGGATGGACCCCC-GCGGTTCCAT---CGAGGGCACGCCACCCTCGGATCGAACGCTCCTGCGTCGT---CGTGCACTTCTCCCCTAGTAGAACGTCGCGACCCGTTGTGTGTCGGTCTACGGCCCGAGCGGGAGACTGTCGCGTCGCTTCGGCGCACGCGGCAGACCCTCGGTCGCCCGGCCGGCTGCACGACGGTACACTCACGGTATCGGGCCGCAGCCAATCCATT--CTCGAATGTGTGTGCGTCCATCCCGCCGCAAGCTCGGTCAGTTCTTACCCGGAGGCCACGGACCCAGTGCCGTCCCCGGGCCTGGCCAGCTGTTAGCGGGCGGTGTCCTCGGACCGGCCAAACCTCGGATTACCGGTCGGCGACGCTATTGCTTTGGGTACTCTCAGGACCCGTCTTGAAACACGGACCAAGGAGTCTAACATGTGCGCGAGTCATTGGGATGCATAAACCTAAAGGCGAAATGAAAGTGAAAGTCGGCCTTCGCGCCGATCGAGGGAGGATGGGCCGCGTAACAATGCGGCCCCGCACTCCCGGGGCGTCTCGTTCTCACTGCGAGAAGAGGCG

>HT17 NARATBC12

GGGATTCTTTATATAATTTTTGCTATCTGGTCAGGCATTGCTGGTTCTTCATTTAGAATACTTATTCGAATAGAACTTAATAGACCTGGGACTTGAATTAGTAATGATCAGATCTACAACTCCATTGTAACTAGGCATGCATTTCTAATAATTTTCTTTATAGTAATGCCTTTTATAATTGGAGGGTTTGGAAACTTTCTAATTCCAATAATACTAGGATCTCCTGATATAGCTTTCCCCCGAATAAACAATATTAGGTTTTGACTATTACCCCCCTCACTTATACTGCTAATAACAAATAATTTATTTTTTCCCAATTCAGGAACTGGATGGACTGTGTACCCTCCTCTTTCCCTTTACATATACCATTCATCTCCTTCTGTAGACTTTACTATTTTTTCAATTCATATGACAGGAATTTCATCAATTCTTGGGTCACTTAACTTTATTGTAACAATTTTTATAATAAAGAATTTTTCTTTAAAGTACGATCAGATTAATCTTTTTTCATGATCTATCTCGATTACTGTGATTTTACTAATTATTTCTCTCCCAGTTCTAGCAGGAGCAATTACTATGCTGTTATTTGATCGAAATTTTAATACATCATTTTTTGATCCGATAGGAGGAGGAGATCCGATTCTTTATCAACATTTATTTATCTGCCCAATGAATTT-----TTTTAATGGCTGCAGTATAACTGACTGTACAAAGGTAGCAT---CAATTGGTTTT---ATGAAATCTGGAATGAAAGGATTAATGAAATATGTACTGTCTCAATTGTACAACTATGAAATTAAAATTTTAATAAAAATGTTAAAATTCACTTATGGGACGATAAGACCCTATAGAATTTTATATTGAAATTACTCAGTAGTTAATTCAGAAATAATTTCAATATTTGATTGGGAGGATTAT---ATT---TCAACTT--TAATT---GTTAACTTTAATTTAAGA-GTAAATAATGATCTTCAATTTGAAATTGCTAGAATAAATTACCTTAGGGATAACAGCGTAATACTTTTTTATAGGTCATATAGAAAAAAGTGGTTGCGACCTCGATGTTGAATTAGGATAAATTTTAAATGCAGGAGTTTAATAATTAAGTCTGTCGTCGACGGCGTAGGCTACCGTTGGTGCGCGATGCTCCGGATGGACCCCC-GCGGTTCCAT---CGAGGGCACGCCACCCTCGGATCGAACGCTCCTGCGTCGT---CGTGCACTTCTCCCCTAGTAGAACGTCGCGACCCGTTGTGTGTCGGTCTACGGCCCGAGCGGGAGACTGTCGCGTCGCTTCGGCGCACGCGGCAGACCCTCGGTCGCCCGGCCGGCTGCACGACGGTACACTCACGGTATCGGGCCGCAGCCAATCCATT--CTCGAATGTGTGTGCGTCCATCCCGCCGCAAGCTCGGTCAGTTCTTACCCGGAGGCCACGGACCCAGTGCCGTCCCCGGGCCTGGCCAGCTGTTAGCGGGCGGTGTCCTCGGACCGGCCAAACCTCGGATTACCGGTCGGCGACGCTATTGCTTTGGGTACTCTCAGGACCCGTCTTGAAACACGGACCAAGGAGTCTAACATGTGCGCGAGTCATTGGGATGCATAAACCTAAAGGCGAAATGAAAGTGAAAGTCGGCCTTCGCGCCGATCGAGGGAGGATGGGCCGCGTAACAATGCGGCCCCGCACTCCCGGGGCGTCTCGTTCTCACTGCGAGAAGAGGCG

>HT18 NARATBC13

GGGATTCTGTATATAATTTTTGCTATCTGATCAGGTATTGCTGGTTCTTCCTTCAGAATACTTATTCGAATGGAACTTAATAGCCCTGGAACTTGAATTAGTAATGATCAGATCTACAACTCTATTGTAACTAGGCATGCATTTCTGATAATTTTTTTTATAGTAATACCTTTTATAATTGGAGGGTTTGGAAATTTTTTGATTCCACTAATACTAGGATCTCCTGATATAGCTTTTCCTCGAATAAACAATATTAGCTTTTGACTTCTACCTCCTTCACTTATACTGCTAATAACAAATAATTTATTTTTTCCAAATTCAGGAACTGGATGAACTGTGTACCCTCCTCTTTCTCTCTACATATACCATTCATCCCCTTCAGTAGACTTTACTATTTTTTCAATCCATATAACAGGAATTTCATCAATTCTTGGATCACTTAACTTTATTGTAACAATTTTTATAATAAAGAATTTTTCTTTAAAGTACGATCAAATTAATCTTTTTTCATGATCTATCTCAATTACTGTAATTTTACTAATCCTTTCTCTCCCAGTTCTAGCGGGAGCAATTACTATGCTGTTATTTGACCGAAATTTTAACACTTCCTTCTTTGATCCAATAGGAGGGGGAGATCCAATTCTTTATCAACATCTATTTATCTGCCCAATGAATTT-----TTTTAATGGCTGCAGTATAACTGACTGTACAAAGGTAGCAT---CAATTGGTTTT---ATGAAATCTGGAATGAAAGGATTAATGAAATATGTACTGTCTCAATTGTACAATTATGAAATTAAAATTTTAATAAAAATGTTAAAATTCACTTATGGGACGATAAGACCCTATAGAATTTTATATTGAAATTACTCAGTAGTTAATTCAGAAATAGTTTCAATATTTGATTGGGAGGATTAT---AAT---CCAACTT--TAATT---GTTAACTTTAATTTAAGA-GTAGATAATGATCTTCAATTTGAAATTGCTAGAATAAATTACCTTAGGGATAACAGCGTAATACTTTTTTATAGGCCATATAGAAAAAAGTGGTTGCGACCTCGATGTTGAATTAGGATAAATTTTAAATGCAGGAGTTTAATAATTAAGTCTGTCGTCGACGGCGTAGGCTACCGTTGGTGCGCGATGCTCCGGATGGACCCCC-GCGGTTCCAT---CGAGGGCACGCCACCCTCGGATCGAACGCTCCTGCGTCGT---CGTGCACTTCTCCCCTAGTAGAACGTCGCGACCCGTTGTGTGTCGGTCTACGGCCCGAGCGGGAGACTGTCGCGTCGCTTCGGCGCACGCGGCAGACCCTCGGTCGCCCGGCCGGCTGCACGACGGTACACTCACGGTATCGGGCCGCAGCCAATCCATT--CTCGAATGTGTGTGCGTCCATCCCGCCGCAAGCTCGGTCAGTTCTTACCCGGAGGCCACGGACCCAGTGCCGTCCCCGGGCCTGGCCAGCTGTTAGCGGGCGGTGTCCTCGGACCGGCCAAACCTCGGATTACCGGTCGGCGACGCTATTGCTTTGGGTACTCTCAGGACCCGTCTTGAAACACGGACCAAGGAGTCTAACATGTGCGCGAGTCATTGGGATGCATAAACCTAAAGGCGAAATGAAAGTGAAAGTCGGCCTTCGCGCCGATCGAGGGAGGATGGGCCGCGTAACAATGCGGCCCCGCACTCCCGGGGCGTCTCGTTCTCACTGCGAGAAGAGGCG

>HT19 KBMNC14

GGAATTCTATATATAATTTTTGCTATCTGATCAGGTATTGTTGGTTCTTCCTTCAGAATACTTATTCGAATGGAACTTAATAGCCCTGGAACTTGAATTAGTAATGATCAGATCTACAACTCTATTGTGACTAGGCATGCATTTCTAATAATTTTTTTTATAGTTATACCTTTTATAATCGGGGGTTTTGGAAATTTTTTAATCCCGTTAATACTAGGTTCTCCTGATATAGCTTTCCCTCGAATAAACAATATTAGCTTCTGACTACTACCTCCTTCACTTATACTGCTAATAACAAATAATCTATTTTTTCCGAATTCAGGAACTGGATGAACTGTATACCCTCCTCTTTCTCTCTACATGTATCATCCATCTCCGTCAGTGGACTTCACTATTTTTTCAATTCATATGACAGGAATTTCATCAATTCTGGGGTCACTTAACTTTATTGTGACAATTTTTATAATAAAGAACTTTTCTTTAAAGTACGATCAAATTAATCTTTTTTCATGATCTATCTCAATTACTGTAATTTTACTAATTCTTTCTCTTCCAGTTCTAGCAGGAGCAATTACTATGCTGTTATTTGATCGAAATTTTAATACTTCCTTCTTTGATCCAATAGGAGGGGGAGACCCGATTCTTTATCAACATTTATTTATCTGCCCAATGAATTT-----TTTTAATGGCTGCAGTATAACTGACTGTACAAAGGTAGCAT---CAATTGGTTTT---ATGAAATCTGGAATGAAAGGATTAATGAAATATGTACTGTCTCAATTGTACAATTATGAAATTAAAATTTTAATAAAAATGTTAAAATTCACTTATGGGACGATAAGACCCTATAGAATTTTATATTGAAATTACTCAGTAGTTAATTCAGAAATAGTTTCAATATTTGATTGGGAGGATTAT---ACT---CCAACTT--TAATT---GTTAACTTTAATTTAAGA-GTAGATAATGATCTTCAATTTGAAATTGCTAGAATAAATTACCTTAGGGATAACAGCGTAATACTTTTTTATAGGCCATATAGAAAAAAGTGGTTGCGACCTCGATGTTGAATTAGGATAAATTTTAAATGCAGGAGTTTAATAATTAAGTCTGTCGTCGACGGCGTAGGCTACCGTTGGTGCGCGATGCTCCGGATGGACCCCC-GCGGTTCCAT---CGAGGGCACGCCACCCTCGGATCGAACGCTCCTGCGTCGT---CGTGCACTTCTCCCCTAGTAGAACGTCGCGACCCGTTGTGTGTCGGTCTACGGCCCGAGCGGGAGACTGTCGCGTCGCTTCGGCGCACGCGGCAGACCCTCGGTCGCCCGGCCGGCTGCACGACGGTACACTCACGGTATCGGGCCGCAGCCAATCCATT--CTCGAATGTGTGTGCGTCCATCCCGCCGCAAGCTCGGTCAGTTCTTACCCGGAGGCCACGGACCCAGTGCCGTCCCCGGGCCTGGCCAGCTGTTAGCGGGCGGTGTCCTCGGACCGGCCAAACCTCGGATTACCGGTCGGCGACGCTATTGCTTTGGGTACTCTCAGGACCCGTCTTGAAACACGGACCAAGGAGTCTAACATGTGCGCGAGTCATTGGGATGCATAAACCTAAAGGCGAAATGAAAGTGAAAGTCGGCCTTCGCGCCGATCGAGGGAGGATGGGCCGCGTAACAATGCGGCCCCGCACTCCCGGGGCGTCTCGTTCTCACTGCGAGAAGAGGCG

>HT20 KBMNC15

GGAATTCTTTATATAATTTTTGCTATCTGATCAGGTATTGTTGGTTCTTCCTTCAGAATACTTATTCGAATGGAACTTAATAGCCCTGGAACTTGAATTAGTAATGATCAGATCTACAACTCTATTGTGACTAGGCATGCATTTCTAATAATTTTTTTTATAGTTATACCTTTTATAATCGGGGGTTTTGGAAATTTTTTAATCCCGTTAATACTAGGTTCTCCTGATATAACTTTCCCTCGAATAAACAATATTAGCTTCTGACTACTACCTCCTTCACTTATACTGCTAATAACAAATAATCTATTTTTTCCGAATTCAGGAACTGGATGAACTGTATACCCTCCTCTTTCTCTCTACATGTATCATCCATCTCCGTCAGTGGACTTCACTATTTTTTCAATTCATATGACAGGAATTTCATCAATTCTGGGGTCACTTAACTTTATTGTGACAATTTTTATAATAAAGAACTTTTCTTTAAAGTACGATCAAATTAATCTTTTTTCATGATCTATCTCAGTTACTGTAATTTTACTAATTCTTTCTCTTCCAGTTCTAGCAGGAGCAATTACTATGCTGTTATTTGATCGAAATTTTAATACTTCCTTCTTTGATCCAATAGGAGGGGGAGACCCGATTCTTTATCAACATTTATTTATCTGCCCAATGAATTT-----TTTTAATGGCTGCAGTATAACTGACTGTACAAAGGTAGCAT---CAATTGGTTTT---ATGAAATCTGGAATGAAAGGATTAATGAAATATGTACTGTCTCAATTGTACAATTATGAAATTAAAATTTTAATAAAAATGTTAAAATTCACTTATGGGACGATAAGACCCTATAGAATTTTATATTGAAATTACTCAGTAGTTAATTCAGAAATAGTTTCAATATTTGATTGGGAGGATTAT---ACT---CCAACTT--TAATT---GTTAACTTTAATTTAAGA-GTAGATAATGATCTTCAATTTGAAATTGCTAGAATAAATTACCTTATGGATAACAGCGTAATACTTTTTTATAGGCCATATAAAAAAAAGTGGTTGCGACCTCTATGTTGAATTATGATAAATTTTAAATGCAGGAGTTTATTAATTAAATCTGTCGTCGACGGCGTAGGCTACCGTTGGTGCGCGATGCTCCGGATGGACCCCC-GCGGTTCCAT---CGAGGGCACGCCACCCTCGGATCGAACGCTCCTGCGTCGT---CGTGCACTTCTCCCCTAGTAGAACGTCGCGACCCGTTGTGTGTCGGTCTACGGCCCGAGCGGGAGACTGTCGCGTCGCTTCGGCGCACGCGGCAGACCCTCGGTCGCCCGGCCGGCTGCACGACGGTACACTCACGGTATCGGGCCGCAGCCAATCCATT--CTCGAATGTGTGTGCGTCCATCCCGCCGCAAGCTCGGTCAGTTCTTACCCGGAGGCCACGGACCCAGTGCCGTCCCCGGGCCTGGCCAGCTGTTAGCGGGCGGTGTCCTCGGACCGGCCAAACCTCGGATTACCGGTCGGCGACGCTATTGCTTTGGGTACTCTCAGGACCCGTCTTGAAACACGGACCAAGGAGTCTAACATGTGCGCGAGTCATTGGGATGCATAAACCTAAAGGCGAAATGAAAGTGAAAGTCGGCCTTCGCGCCGATCGAGGGAGGATGGGCCGCGTAACAATGCGGCCCCGCACTCCCGGGGCGTCTCGTTCTCACTGCGAGAAGAGGCG

>HT21 KBMNC16

GGAATTCTTTATATAATTTTTGCTATCTGATCAGGTATTGTTGGTTCTTCCTTCAGAATACTTATTCGAATGGAACTTAATACCCCTGGAACTTGAATTAGTAATGATCAGATCTACAACTCTATTGTGACTAGGCATGCATTTCTAATAATTTTTTTTAAAGTTATACCTTTTATAATCGGGGGTTTTGGAAATTTTTTAATCCCGTTAATACTAGGTTCTCCTGATATAACTTTCCCTCAAATAAACAATATTAACTTCTGACTACTACCTCCTTCACTTATACTGCTAATAACAAATAATCTATTTTTTCCGAATTCATGAACTGGATGAACTGTATACCCTCCTCTTTCTCTCTACATGTATCATCCATCTCCGTCAGTGGACTTCACTATTTTTTCAATTCATATGACAGGAATTTCATCAATTCTGGGGTCACTTAACTTTATTGTGACAATTTTTATAATAAAAAACTTTTCTTTAAAGTACGATCAAATTAATCTTTTTTCATGATCTATCTCAATTACTGTAATTTTACTATTTCTTTCTCTTCCATTTCTAGCAGGAGCAATTACTATGCTGTTATTTGATCGAAATTTTAATACTTCCTTCTTTGATCCAATAGGAGGGGGAGACCCGATTCTTTATCAACATTTATTTATCTGCCCAATGAATTT-----TTTTAATGGCTGCAGTATAACTGACTGTACAAAGGTAGCAT---CAATTGGTTTT---ATGAAATCTGGAATGAAAGGATTAATGAAATATGTACTGTCTCAATTGTACAATTATGAAATTAAAATTTTAATAAAAATGTTAAAATTCACTTATGGGACGATAAGACCCTATAGAATTTTATATTGAAATTACTCAGTAGTTAATTCAGAAATAGTTTCAATATTTGATTGGGAGGATTAT---ACT---CCAACTT--TAATT---GTTAACTTTAATTTAAGA-GTAGATAATGATCTTCAATTTGAAATTGCTAGAATAAATTACCTTAGGGATAACAGCGTAATACTTTTTTATAGGCCATATAGAAAAAAGTGGTTGCGACCTCGATGTTGAATTAGGATAAATTTTAAATGCAGGAGTTTAATAATTAAGTCTGTCGTCGACGGCGTAGGCTACCGTTGGTGCGCGATGCTCCGGATGGACCCCC-GCGGTTCCAT---CGAGGGCACGCCACCCTCGGATCGAACGCTCCTGCGTCGT---CGTGCACTTCTCCCCTAGTAGAACGTCGCGACCCGTTGTGTGTCGGTCTACGGCCCGAGCGGGAGACTGTCGCGTCGCTTCGGCGCACGCGGCAGACCCTCGGTCGCCCGGCCGGCTGCACGACGGTACACTCACGGTATCGGGCCGCAGCCAATCCATT--CTCGAATGTGTGTGCGTCCATCCCGCCGCAAGCTCGGTCAGTTCTTACCCGGAGGCCACGGACCCAGTGCCGTCCCCGGGCCTGGCCAGCTGTTAGCGGGCGGTGTCCTCGGACCGGCCAAACCTCGGATTACCGGTCGGCGACGCTATTGCTTTGGGTACTCTCAGGACCCGTCTTGAAACACGGACCAAGGAGTCTAACATGTGCGCGAGTCATTGGGATGCATAAACCTAAAGGCGAAATGAAAGTGAAAGTCGGCCTTCGCGCCGATCGAGGGAGGATGGGCCGCGTAACAATGCGGCCCCGCACTCCCGGGGCGTCTCGTTCTCACTGCGAGAAGAGGCG

>HT22 KBMNC17

GGAATTCTATATATAATTTTTGCTATCTGATCAGGTATTGTTGGTTCTTCCTTCAGAATACTTATTCGAATGGAACTTAATAGCCCTGGAACTTGAATTAGTAATGATCAGATCTACAACTCTATTGTGACTAGGCATGCATTTCTAATAATTTTTTTTATAGTTATACCTTTTATAATCGGGGGTTTTGGAAATTTTTTAATCCCGTTAATACTAGGTTCTCCTGATATAGCTTTCCCTCGAATAAACAATATTAGCTTCTGACTACTACCTCCTTCACTTATACTGCTAATAACAAATAATCTATTTTTTCCGAATTCAGGAACTGGATGAACTGTATACCCTCCTCTTTCTCTCTACATGTATCATCCATCTCCGTCAGTGGACTTCACTATTTTTTCAATTCATATGACAGGAATTTCATCAATTCTGGGGTCACTTAACTTTATTGTGACAATTTTTATAATAAAGAACTTTTCTTTAAAGTACGATCAAATTAATCTTTTTTCATGATCTATCTCAATTACTGTAATTTTACTAATTCTTTCTCTTCCAGTTCTAGCAGGAGCAATTACTATGCTGTTATTTGATCGAAATTTTAATACTTCCTTCTTTGATCCAATAGGAGGGGGAGACCCGATTCTTTATCAACATTTATTTATCTGCCCAATGAATTT-----TTTTAATGGCTGCAGTATAACTGACTGTACAAAGGTAGCAT---CAATTGGTTTT---ATGAAATCTGGAATGAAAGGATTAATGAAATATGTACTGTCTCAATTGTACAATTATGAAATTAAAATTTTAATAAAAATGTTAAAATTCACTTATGGGACGATAAGACCCTATAGAATTTTATATTGAAATTACTCAGTAGTTAATTCAGAAATAGTTTCAATATTTGATTGGGAGGATTAT---ACT---CCAACTT--TAATT---GTTAACTTTAATTTAAGA-GTAGATAATGATCTTCAATTTGAAATTGCTAGAATAAATTACCTTAGGGATAACAGCGTAATACTTTTTTATAGGCCATATAGAAAAAAGTGGTTGCGACCTCGATGTTGAATTAGGATAAATTTTAAATGCAGGAGTTTAATAATTAAGTCTGTCGTCGACGGCGTAGGCTACCGTTGGTGCGCGATGCTCCGGATGGACCCCC-GCGGTTCCAT---CGAGGGCACGCCACCCTCGGATCGAACGCTCCTGCGTCGT---CGTGCACTTCTCCCCTAGTAGAACGTCGCGACCCGTTGTGTGTCGGTCTACGGCCCGAGCGGGAGACTGTCGCGTCGCTTCGGCGCACGCGGCAGACCCTCGGTCGCCCGGCCGGCTGCACGACGGTACACTCACGGTATCGGGCCGCAGCCAATCCATT--CTCGAATGTGTGTGCGTCCATCCCGCCGCAAGCTCGGTCAGTTCTTACCCGGAGGCCACGGACCCAGTGCCGTCCCCGGGCCTGGCCAGCTGTTAGCGGGCGGTGTCCTCGGACCGGCCAAACCTCGGATTACCGGTCGGCGACGCTATTGCTTTGGGTACTCTCAGGACCCGTCTTGAAACACGGACCAAGGAGTCTAACATGTGCGCGAGTCATTGGGATGCATAAACCTAAAGGCGAAATGAAAGTGAAAGTCGGCCTTCGCGCCGATCGAGGGAGGATGGGCCGCGTAACAATGCGGCCCCGCACTCCCGGGGCGTCTCGTTCTCACTGCGAGAAGAGGCG

>HT23 KBMNC18

GGAATTCTATATATAATTTTTGCTATCTGATCAGGTATTGTTGGTTCTTCCTTCAGAATACTTATTCGAATGGAACTTAATAGCCCTGGAACTTGAATTAGTAATGATCAAATCTACAACTCTATTGTGACTAGGCATGCATTTCTAATAATTTTTTTTATAGTTATACCTTTTATAATCGGGGGTTTTGGAAATTTTTTAATCCCGTTAATACTAGGTTCTCCTGATATAGCTTTCCCTCGAATAAACAATATTAGCTTCTGACTACTACCTCCTTCACTTATACTGCTAATAACAAATAATCTATTTTTTCCGAATTCAGGAACTGGATGAACTGTATACCCTCCTCTTTCTCTCTACATGTATCATCCATCTCCGTCAGTGGACTTCACTATTTTTTCAATTCATATGACAGGAATTTCATCAATTCTGGGGTCACTTAACTTTATTGTGACAATTTTTATAATAAAGAACTTTTCTTTAAAGTACGATCAAATTAATCTTTTTTCATGATCTATCTCAATTACTGTAATTTTACTAATTCTTTCTCTTCCAGTTCTAGCAGGAGCAATTACTATGCTGTTATTTGATCGAAATTTTAATACTTCCTTCTTTGATCCAATAGGAGGGGGAGACCCGATTCTTTATCAACATTTATTTATCTGCCCAATGAATTT-----TTTTAATGGCTGCAGTATAACTGACTGTACAAAGGTAGCAT---CAATTGGTTTT---ATGAAATCTGGAATGAAAGGATTAATGAAATATGTACTGTCTCAATTGTACAATTATGAAATTAAAATTTTAATAAAAATGTTAAAATTCACTTATGGGACGATAAGACCCTATAGAATTTTATATTGAAATTACTCAGTAGTTAATTCAGAAATAGTTTCAATATTTGATTGGGAGGATTAT---ACT---CCAACTT--TAATT---GTTAACTTTAATTTAAGA-GTAGATAATGATCTTCAATTTGAAATTGCTAGAATAAATTACCTTAGGGATAACAGCGTAATACTTTTTTATAGGCCATATAGAAAAAAGTGGTTGCGACCTCGATGTTGAATTAGGATAAATTTTAAATGCAGGAGTTTAATAATTAAGTCTGTCGTCGACGGCGTAGGCTACCGTTGGTGCGCGATGCTCCGGATGGACCCCC-GCGGTTCCAT---CGAGGGCACGCCACCCTCGGATCGAACGCTCCTGCGTCGT---CGTGCACTTCTCCCCTAGTAGAACGTCGCGACCCGTTGTGTGTCGGTCTACGGCCCGAGCGGGAGACTGTCGCGTCGCTTCGGCGCACGCGGCAGACCCTCGGTCGCCCGGCCGGCTGCACGACGGTACACTCACGGTATCGGGCCGCAGCCAATCCATT--CTCGAATGTGTGTGCGTCCATCCCGCCGCAAGCTCGGTCAGTTCTTACCCGGAGGCCACGGACCCAGTGCCGTCCCCGGGCCTGGCCAGCTGTTAGCGGGCGGTGTCCTCGGACCGGCCAAACCTCGGATTACCGGTCGGCGACGCTATTGCTTTGGGTACTCTCAGGACCCGTCTTGAAACACGGACCAAGGAGTCTAACATGTGCGCGAGTCATTGGGATGCATAAACCTAAAGGCGAAATGAAAGTGAAAGTCGGCCTTCGCGCCGATCGAGGGAGGATGGGCCGCGTAACAATGCGGCCCCGCACTCCCGGGGCGTCTCGTTCTCACTGCGAGAAGAGGCG

>HT24 KBMNC19

GGGATTCTGTATATAATTTTTGCTATCTGATCAGGTATTGCTGGTTCTTCCTTCAGAATACTTATTCGAATGGAACTTAATAGCCCTGGAACTTGAATTAGTAATGATCAGATCTACAACTCTATTGTAACTAGGCATGCATTTCTGATAATTTTTTTTATAGTAATACCTTTTATAATTGGAGGGTTTGGAAATTTTTTGATTCCACTAATACTAGGATCTCCTGATATAGCTTTTCCTCAAATAAACAATATTAGCTTTTGACTACTACCTCCTTCACTTATACTGCTAATAACAAATAATTTATTATTTCCAAATTCAGGAACTGGATGAACTGTGTATCCTCCTCTTTCTCTCTACATATACCATCCATCTCCTTCAGTAGACTTTACTATTTTTTCAATCCATATAACAGGAATTTCATCAATTCTTGGATCACTTAACTTTATTGTAGCAATCTTTATAATAAAGAATTTTTCTTTAAAGTACGATCAAATTAATCTTTTTTCATGATCTATCTCAGTTACTGTAATTTTACTAATCCTTTCTCTCCCATTTCTAGCAGGGGCAATTACTATGCTATTATTTGACCGAAATTTTAATACTTCCTTCTTTGATCCAATAGGAGGAGGAGATCCAATTCTTTATCAACATCTATTTATCTGCCCAATGAGTTT-----TTTTAATGGCTGCAGTATAACTGACTGTACAAAGGTAGCAT---CAATTGGTTTT---ATGAAATCTGGAATGAAAGGATTAATGAAATATGTACTGTCTCAATTGTACAATTATGAAATTAAAATTTTAATAAAAATGTTAAAATTCACTTATGGGACGATAAGACCCTATAGAATTTTATATTGAAATTACTCAGTAGTTAATTCAGAAATAGTTTCAATATTTGATTGGGAGGATTAT---AAT---CCAACTT--TAATT---GTTAACTTTAATTTAAGA-GTAGATAATGATCTTCAATTTGAAATTGCTAGAATAAATTACCTTAGGGATAACAGCGTAATACTTTTTTATAGGCCATATAGAAAAAAGTGGTTGCGACCTCGATGTTGAATTAGGATAAATTTTAAATGCAGGAGTTTAATAATTAAGTCTGTCGTCGACGGCGTAGGCTACCGTTGGTGCGCGATGCTCCGGATGGACCCCC-GCGGTTCCAT---CGAGGGCACGCCACCCTCGGATCGAACGCTCCTGCGTCGT---CGTGCACTTCTCCCCTAGTAGAACGTCGCGACCCGTTGTGTGTCGGTCTACGGCCCGAGCGGGAGACTGTCGCGTCGCTTCGGCGCACGCGGCAGACCCTCGGTCGCCCGGCCGGCTGCACGACGGTACACTCACGGTATCGGGCCGCAGCCAATCCATT--CTCGAATGTGTGTGCGTCCATCCCGCCGCAAGCTCGGTCAGTTCTTACCCGGAGGCCACGGACCCAGTGCCGTCCCCGGGCCTGGCCAGCTGTTAGCGGGCGGTGTCCTCGGACCGGCCAAACCTCGGATTACCGGTCGGCGACGCTATTGCTTTGGGTACTCTCAGGACCCGTCTTGAAACACGGACCAAGGAGTCTAACATGTGCGCGAGTCATTGGGATGCATAAACCTAAAGGCGAAATGAAAGTGAAAGTCGGCCTTCGCGCCGATCGAGGGAGGATGGGCCGCGTAACAATGCGGCCCCGCACTCCCGGGGCGTCTCGTTCTCACTGCGAGAAGAGGCG

>HT25 KBMNC20

GGAATTCTGTATATAATTTTTGCTATCTGATCAGGTATTGTTGGTTCTTCCTTCAGAATACTTATTCGAATGGAACTTAATAGCCCTGGAACTTGAATTAGTAATGATCAGATCTACAACTCTATTGTGACTAGGCATGCATTTCTAATAATTTTTTTTATAGTAATACCTTTTATAATCGGAGGTTTTGGAAATTTTTTAATTCCGCTAATACTAGGTTCTCCTGATATAACTTTCCCCCGAATGAACAATATCAGCTTCTGACTACTACCTCCTTCACTTATACTGCTAATAACAAATAATTTATTCTTTCCGAATTCAGGAACTGGATGAACTGTATACCCCCCTCTTTCTCTCTACATATATCATTCATCTCCGTCAGTGGACTTTACTATTTTTTCAATTCATATAACGGGAATTTCATCAATTCTTGGGTCACTTAACTTTATTGTAACAATTTTTATAATAAAGAACTTTTCTTTGAAGTACGACCAAATTAATCTTTTTTCATGATCTATCTCAGTTACTGTAATTTTACTAATTCTTTCTCTTCCAGTTCTAGCAGGAGCAATTACTATGCTGTTATTTGATCGAAATTTTAATACTTCCTTCTTTGATCCAATAGGAGGGGGAGACCCGATTCTTTATCAACATTTATTTATCTGCCCAATGAATTC-----TTTTAATGGCTGCAGTATAACTGACTGTACAAAGGTAGCAT---CAATTGGTTTT---ATGAAATCTGGAATGAAAGGATTAATGAAATATGTACTGTCTCAATTGTACAATTATGAAATTAAAATTTTAATAAAAATGTTAAAATTCACTTATGGGACGATAAGACCCTATAGAATTTTATATTGAAATTACTCAGTAGTTAATTCAGAAATAGTTTCAATATTTGATTGGGAGGATTAT---ACT---CCAACTT--TAATT---GTTAACTTTAATTTAAGA-GTAGATAATGATCTTCAATTTGAAATTGCTAGAATAAATTACCTTAGGGATAACAGCGTAATACTTTTTTATAGGCCATATAGAAAAAAGTGGTTGCGACCTCGATGTTGAATTAGGATAAATTTTAAATGCAGGAGTTTAATAATTAAGTCTGTCGTCGACGGCGTAGGCTACCGTTGGTGCGCGATGCTCCGGATGGACCCCC-GCGGTTCCAT---CGAGGGCACGCCACCCTCGGATCGAACGCTCCTGCGTCGT---CGTGCACTTCTCCCCTAGTAGAACGTCGCGACCCGTTGTGTGTCGGTCTACGGCCCGAGCGGGAGGCTGTCGCGTCGCTTCGGCGCACGCGGCAGACCCTCGGTCGCCCGGCCGGCTGCACGACGGTACACTCACGGTATCGGGCCGCAGCCAATCCATT--CTCGAATGTGTGTGCGTCCATCCCGCCGCAAGCTCGGTCAGTTCTTACCCGGAGGCCACGGACCCAGTGCCGTCCCCGGGCCTGGCCAGCTGTTAGCGGGCGGTGTCCTCGGACCGGCCAAACCTCGGATTACCGGTCGGCGACGCTATTGCTTTGGGTACTCTCAGGACCCGTCTTGAAACACGGACCAAGGAGTCTAACATGTGCGCGAGTCATTGGGATGCATAAACCTAAAGGCGAAATGAAAGTGAAAGTCGGCCTTCGCGCCGATCGAGGGAGGATGGGCCGCGTAACAATGCGGCCCCGCACTCCCGGGGCGTCTCGTTCTCACTGCGAGAAGAGGCG

>HT26 KBMNC21

GGAATTCTGTATATAATTTTTGCTATCTGATCAGGTATTGTTGGTTCTTCCTTCAGAATACTTATTCGAATGGAACTTAATAGCCCTGGAACTTGAATTAGTAATGATCAGATCTACAACTCTATTGTGACTAGGCATGCATTTCTAATAATTTTTTTTATAGTAATACCTTTTATAATCGGAGGTTTTGGAAATTTTTTAATTCCGCTAATACTAGGTTCTCCTGATATAACTTTCCCCCGAATGAACAATATCAGCTTCTGACTACTACCTCCTTCACTTATACTGCTAATAACAAATAATTTATTCTTTCCGAATTCAGGAACTGGATGAACTGTATACCCCCCTCTTTCTCTCTACATATATCATTCATCTCCGTCAGTGGACTTTACTATTTTTTCAATTCATATAACGGGAATTTCATCAATTCTTGGGTCACTTAACTTTATTGTAACAATTTTTATAATAAAGAACTTTTCTTTGAAGTACGACCAAATTAATCTTTTTTCATGATCTATCTCAGTTACTGTAATTTTACTAATTCTTTCTCTTCCAGTTCTAGCAGGAGCAATTACTATGCTGTTATTTGATCGAAATTTTAATACTTCCTTCTTTGATCCAATAGGAGGGGGAGACCCGATTCTTTATCAACATTTATTTATCTGCCCAATGAATTC-----TTTTAATGGCTGCAGTATAACTGACTGTACAAAGGTAGCAT---CAATTGGTTTT---ATGAAATCTGGAATGAAAGGATTAATGAAATATGTACTGTCTCAATTGTACAATTATGAAATTAAAATTTTAATAAAAATGTTAAAATTCACTTATGGGACGATAAGACCCTATAGAATTTTATATTGAAATTACTCAGTAGTTAATTCAGAAATAGTTTCAATATTTGATTGGGAGGATTAT---ACT---CCAACTT--TAATT---GTTAACTTTAATTTAAGA-GTAGATAATGATCTTCAATTTGAAATTGCTAGAATAAATTACCTTAGGGATAACAGCGTAATACTTTTTTATAGGCCATATAGAAAAAAGTGGTTGCGACCTCGATGTTGAATTAGGATAAATTTTAAATGCAGGAGTTTAATAATTAAGTCTGTCGTCGACGGCGTAGGCTACCGTTGGTGCGCGATGCTCCGGATGGACCCCC-GCGGTTCCAT---CGAGGGCACGCCACCCTCGGATCGAACGCTCCTGCGTCGT---CGTGCACTTCTCCCCTAGTAGAACGTCGCGACCCGTTGTGTGTCGGTCTACGGCCCGAGCGGGAGGCTGTCGCGTCGCTTCGGCGCACGCGGCAGACCCTCGGTCGCCCGGCCGGCTGCACGACGGTACACTCACGGTATCGGGCCGCAGCCAATCCATT--CTCGAATGTGTGTGCGTCCATCCCGCCGCAAGCTCGGTCAGTTCTTACCCGGAGGCCACGGACCCAGTGCCGTCCCCGGGCCTGGCCAGCTGTTAGCGGGCGGTGTCCTCGGACCGGCCAAACCTCGGATTACCGGTCGGCGACGCTATTGCTTTGGGTACTCTCAGGACCCGTCTTGAAACACGGACCAAGGAGTCTAACATGTGCGCGAGTCATTGGGATGCATAAACCTAAAGGCGAAATGAAAGTGAAAGTCGGCCTTCGCGCCGATCGAGGGAGGATGGGCCGCGTAACAATGCGGCCCCGCACTCCCGGGGCGTCTCGTTCTCACTGCGAGAAGAGGCG

>HT27 KBMNC22

GGAATTCTTTATATAATTTTTGCCATTTGATCTGGCATTGTAGGTTCATCTTTTAGAATACTTATTCGGATGGAGCTTAATAGTCCTGGAACTTGAATTAATAATGACCAAATTTACAATTCTATTGTAACTAGTCATGCATTTCTAATAATTTTCTTTATAGTTATACCCTTTATAATCGGAGGGTTCGGGAATTTTCTAATTCCACTAATACTGGGTTCCCCTGATATAGCTTTTCCTCGAATAAATAACGTTAGATTTTGATTACTCCCACCCTCAATTATACTACTTATAACAAATAATCTATTTTATCCTAATTCAGGAACTGGATGAACTGTATATCCTCCACTTTCTCTTTATATATATCATTCATCTCCATCTGTAGATTTCACTATTTTTTCAATTCATATAACAGGAATTTCATCAATTCTAGGATCTCTTAATTTTATTGTTACAATTTTTATAATAAAAAATTTCTCTTTGAAGTATGATCAAATCAATCTATTTTCATGATCTATCTCAATTACTGTGATCTTACTAATTCTTTCCCTTCCGGTTCTAGCAGGAGCAATTACTATACTTTTGTTTGACCGAAACTTCAACACCTCATTTTTTGATCCAATAGGAGGAGGAGATCCTATCCTGTACCAACATCTGTTCATCTGCCCAATGAAGTT-----TTTTAATGGCTGCAGTATAACTGACTGTACAAAGGTAGCAT---CAATTGGTTTT---ATGAAATCTGGAATGAAAGGATTAATGAAATATGTACTGTCTCAATTGTATGATTACGAAATTAAAATTTTAATAAAAATGTTAAAATTTACTTATGGGACGATAAGACCCTATAGAATTTTATATTGAAATTACTCAGTAGTTAATACAGAAATAATTTTAATATTTGGTTGGGAGGACTAT---ATT---CTAACTT--TAATT---ATTAACTTTAATTTAAGA-GTAAATAAGGATCTTCAATTTGAAATTGTCAGAGTAAATTACCTTAGGGATAACAGCGTAATACTTTTTTATAGGCCACATAGAAAAAAGTGATTGCGACCTCGATGTTGAATTAGGATAAATTTTAAATGCAGGAGTTTAATAATTAAGTCTGTCGTCGACGGCGTAGGCTACCGTTGGTGCGCGATGCTCCGGATGGACCCCC-GCGGTTCCAT---CGAGGGCACGCCACCCTCGGATCGAACGCTCCTGCGTCGT---CGTGCACTTCTCCCCTAGTAGAACGTCGCGACCCGTTGTGTGTCGGTCTACGGCCCGAGCGGGAGACTGTCGCGTCGCTTCGGCGCACGCGGCAGACCCTCGGTCGCCCGGCCGGCTGCACGACGGTACACTCACGGTATCGGGCCGCAGCCAATCCATT--CTCGAATGTGTGTGCGTCCATCCCGCCGCAAGCTCGGTCAGTTCTTACCCGGAGGCCACGGACCCAGTGCCGTCCCCGGGCCTGGCCAGCTGTTAGCGGGCGGTGTCCTCGGACCGGCCAAACCTCGGATTACCGGTCGGCGACGCTATTGCTTTGGGTACTCTCAGGACCCGTCTTGAAACACGGACCAAGGAGTCTAACATGTGCGCGAGTCATTGGGATGCATAAACCTAAAGGCGAAATGAAAGTGAAAGTCGGCCTTCGCGCCGATCGGGGGAGGATGGGCCGCGTAACAATGCGGCCCCGCACTCCCGGGGCGTCTCGTTCTCACTGCGAGAATAGGCG

>HT28 KBMNC23

GGAATTCTTTATATAATTTTTGCCATTTGATCTGGCATTGTAGGTTCATCTTTTAGAATACTTATTCGGATGGAGCTTAATAGTCCTGGAACTTGAATTAATAATGACCAAATTTACAATTCTATTGTAACTAGTCATGCATTTCTAATAATTTTCTTTATAGTTATACCCTTTATAATCGGAGGGTTCGGAAATTTTCTAATTCCACTAATACTGGGTTCCCCTGATATAGCTTTTCCTCGAATAAATAACGTTAGATTTTGATTACTCCCACCCTCAATTATACTACTTATAACAAATAATCTATTTTATCCTAATTCAGGAACTGGATGAACTGTATATCCTCCACTTTCTCTTTATATATATCATTCATCTCCATCTGTAGATTTCACTATTTTTTCAATTCATATAACAGGAATTTCATCAATTCTAGGATCTCTTAATTTTATTGTTACAATTTTTATAATAAAAAATTTCTCTTTGAAGTATGATCAAATCAATCTATTTTCATGATCTATCTCAATTACTGTAATCTTACTAATTCTTTCCCTTCCGGTTCTAGCAGGAGCAATTACTATACTTTTGTTTGACCGAAACTTCAACACCTCATTTTTTGATCCAATAGGAGGAGGAGATCCTATCCTGTACCAACATCTGTTCATCTGCCCAATGAAGTT-----TTTTAATGGCTGCAGTATAACTGACTGTACAAAGGTAGCAT---CAATTGGTTTT---ATGAAATCTGGAATGAAAGGATTAATGAAATATGTACTGTCTCAATTGTATAATTACGAAATTAAAATTTTAATAAAAATGTTAAAATTTACTTATGGGACGATAAGACCCTATAGAATTTTATATTGAAATTACTCAGTAGTTAATACAGAAATAATTTTAATATTTGGTTGGGAGGACTAT---ATT---CTAACTT--TAATT---ATTAACTTTAATTTAAGA-GTAAATAAGGATCTTCAATTTGAAATTGTCAGAGTAAATTACCTTAGGGATAACAGCGTAATACTTTTTTATAGGCCACATAGAAAAAAGTGATTGCGACCTCGATGTTGAATTAGGATAAATTTTAAATGCAGGAGTTTAATAATTAAGTCTGTCGTCGACGGCGTAGGCTACCGTTGGTGCGCGATGCTCCGGATGGACCCCC-GCGGTTCCAT---CGAGGGCACGCCACCCTCGGATCGAACGCTCCTGCGTCGT---CGTGCACTTCTCCCCTAGTAGAACGTCGCGACCCGTTGTGTGTCGGTCTACGGCCCGAGCGGGAGACTGTCGCGTCGCTTCGGCGCACGCGGCAGACCCTCGGTCGCCCGGCCGGCTGCACGACGGTACACTCACGGTATCGGGCCGCAGCCAATCCATT--CTCGAATGTGTGTGCGTCCATCCCGCCGCAAGCTCGGTCAGTTCTTACCCGGAGGCCACGGACCCAGTGCCGTCCCCGGGCCTGGCCAGCTGTTAGCGGGCGGTGTCCTCGGACCGGCCAAACCTCGGATTACCGGTCGGCGACGCTATTGCTTTGGGTACTCTCAGGACCCGTCTTGAAACACGGACCAAGGAGTCTAACATGTGCGCGAGTCATTGGGATGCATAAACCTAAAGGCGAAATGAAAGTGAAAGTCGGCCTTCGCGCCGATCGAGGGAGGATGGGCCGCGTAACAATGCGGCCCCGCACTCCCGGGGCGTCTCGTTCTCACTGCGAGAAGAGGCG

>HT29 KBKLC24

GGGATTCTGTATATAATTTTTGCTATCTGATCAGGTATTGCTGGTTCTTCCTTCAGAATACTTATTCGAATGGAACTTAATAGCCCTGGAACTTGAATTAGTAATGATCAGATCTACAACTCTATTGTAACTAGGCATGCATTTCTGATAATTTTTTTTATAGTAATACCTTTTATAATTGGAGGGTTTGGAAATTTTTTGATTCCGCTAATACTAGGATCTCCTGATATAGCTTTCCCTCGAATAAACAATATTAGCTTTTGACTACTACCTCCTTCACTTATACTGCTAATAACAAATAATTTATTATTTCCAAATTCAGGAACTGGATGAACTGTATACCCTCCTCTTTCTCTCTACATATACCATTCATCCCCGTCAGTAGACTTGACTATTTTTTCAATCCATATAACAGGAATTTCGTCAATTCTTGGATCACTTAACTTTATTGTAACAATTTTTATAATAAAGAATTTTTCTTTAAAGTACGATCAAATTAATCTTTTTTCATGATCTATCTCAATTACTGTAATCTTACTAATCCTTTCTCTCCCAGTTCTAGCAGGAGCAATTACTATGCTGTTATTTGACCGAAATTTTAACACCTCCTTCTTTGATCCAATAGGAGGGGGAGATCCAATTCTTTATCAACATCTATTTATCTGCCCAATGAATTT-----TTTTAATGGCTGCAGTATAACTGACTGTACAAAGGTAGCAT---CAATTGGTTTT---ATGAAATCTGGAATGAAAGGATTAATGAAATATGTACTGTCTCAATTGTACAATTATGAAATTAAAATTTTAATAAAAATGTTAAAATTCACTTATGGGACGATAAGACCCTATAGAATTTTATATTGAAATTACTCAGTAGTTAATTCAGAAATAGTTTCAATATTTGATTGGGAGGATTAT---AAT---CCAACTT--TAATT---GTTAACTTTAATTTAAGA-GTAGATAATGATCTTCAATTTGAAATTGCTAGAATAAATTACCTTAGGGATAACAGCGTAATACTTTTTTATAGGCCATATAGAAAAAAGTGGTTGCGACCTCGATGTTGAATTAGGATAAATTTTAAATGCAGGAGTTTAATAATTAAGTCTGTCGTCGACGGCGTAGGCTACCGTTGGTGCGCGATGCTCCGGATGGACCCCC-GCGGTTCCAT---CGAGGGCACGCCACCCTCGGATCGAACGCTCCTGCGTCGT---CGTGCACTTCTCCCCTAGTAGAACGTCGCGACCCGTTGTGTGTCGGTCTACGGCCCGAGCGGGAGACTGTCGCGTCGCTTCGGCGCACGCGGCAGACCCTCGGTCGCCCGGCCGGCTGCACGACGGTACACTCACGGTATCGGGCCGCAGCCAATCCATT--CTCGAATGTGTGTGCGTCCATCCCGCCGCAAGCTCGGTCAGTTCTTACCCGGAGGCCACGGACCCAGTGCCGTCCCCGGGCCTGGCCAGCTGTTAGCGGGCGGTGTCCTCGGACCGGCCAAACCTCGGATTACCGGTCGGCGACGCTATTGCTTTGGGTACTCTCAGGACCCGTCTTGAAACACGGACCAAGGAGTCTAACATGTGCGCGAGTCATTGGGATGCATAAACCTAAAGGCGAAATGAAAGTGAAAGTCGGCCTTCGCGCCGATCAGGGGAGGATGGGCCGCGTAACAATGCGGCCCCGCACTCCCGGGGCGTCTCGTTCTCACTGCGAGAAGAAGCG

>HT30 KBKLC25

GGGATTCTGTATATAATTTTTGCTATCTGATCAGGTATTGCTGGTTCTTCCTTCAGAATACTTATTCGAATGGAACTTAATAGCCCTGGAACTTGAATTAGTAATGATCAGATCTACAACTCTATTGTAACTAGGCATGCATTTCTGATAATTTTTTTTATAGTAATACCTTTTATAATTGGAGGGTTTGGAAATTTTTTGATTCCGCTAATACTAGGATCTCCTGATATAGCTTTCCCTCGAATAAACAATATTAGCTTTTGACTACTACCTCCTTCACTTATACTGCTAATAACAAATAATTTATTATTTCCAAATTCAGGAACTGGATGAACTGTATACCCTCCTCTTTCTCTCTACATATACCATTCATCCCCGTCAGTAGACTTGACTATTTTTTCAATCCATATAACAGGAATTTCGTCAATTCTTGGATCACTTAACTTTATTGTAACAATTTTTATAATAAAGAATTTTTCTTTAAAGTACGATCAAATTAATCTTTTTTCATGATCTATCTCAATTACTGTAATCTTACTAATCCTTTCTCTCCCAGTTCTAGCAGGAGCAATTACTATGCTGTTATTTGACCGAAATTTTAACACCTCCTTCTTTGATCCAATAGGAGGGGGAGATCCAATTCTTTATCAACATCTATTTATCTGCCCAATGAATTT-----TTTTAATGGCTGCAGTATAACTGACTGTACAAAGGTAGCAT---CAATTGGTTTT---ATGAAATCTGGAATGAAAGGATTAATGAAATATGTACTGTCTCAATTGTACAATTATGAAATTAAAATTTTAATAAAAATGTTAAAATTCACTTATGGGACGATAAGACCCTATAGAATTTTATATTGAAATTACTCAGTAGTTAATTCAGAAATAGTTTCAATATTTGATTGGGAGGATTAT---AAT---CCAACTT--TAATT---GTTAACTTTAATTTAAGA-GTAGATAATGATCTTCAATTTGAAATTGCTAGAATAAATTACCTTAGGGATAACAGCGTAATACTTTTTTATAGGCCATATAGAAAAAAGTGGTTGCGACCTCGATGTTGAATTAGGATAAATTTTAAATGCAGGAGTTTAATAATTAAGTCTGTCGTCGACGGCGTAGGCTACCGTTGGTGCGCGATGCTCCGGATGGACCCCC-GCGGTTCCAT---CGAGGGCACGCCACCCTCGGATCGAACGCTCCTGCGTCGT---CGTGCACTTCTCCCCTAGTAGAACGTCGCGACCCGTTGTGTGTCGGTCTACGGCCCGAGCGGGAGACTGTCGCGTCGCTTCGGCGCACGCGGCAGACCCTCGGTCGCCCGGCCGGCTGCACGACGGTACACTCACGGTATCGGGCCGCAGCCAATCCATT--CTCGAATGTGTGTGCGTCCATCCCGCCGCAAGCTCGGTCAGTTCTTACCCGGAGGCCACGGACCCAGTGCCGTCCCCGGGCCTGGCCAGCTGTTAGCGGGCGGTGTCCTCGGACCGGCCAAACCTCGGATTACCGGTCGGCGACGCTATTGCTTTGGGTACTCTCAGGACCCGTCTTGAAACACGGACCAGGGAGTCTAACATGTGCGCGAGTCATTGGGATGCATAAACCTAAAGGCGAAATGAAAGTGAAAGTCGGCCTTCGCGCCGATCGAGGGAGGATGGGCCGCGTAACAATGCGGCCCCGCACTCCCAGGGCGTCTCGTTCTCACTGCGAGAAGAGGCG

>HT31 KBKLC26

GGGATTCTGTATATAATTTTTGCTATCTGATCAGGTATTGCTGGTTCTTCCTTCAGAATACTTATTCGAATGGAACTTAATAGCCCTGGAACTTGAATTAGTAATGATCAGATCTACAACTCTATTGTAACTAGGCATGCATTTCTGATAATTTTTTTTATAGTAATACCTTTTATAATTGGAGGGTTTGGAAATTTTTTGATTCCGCTAATACTAGGATCTCCTGATATAGCTTTCCCTCGAATAAACAATATTAGCTTTTGACTACTACCTCCTTCACTTATACTGCTAATAACAAATAATTTATTTTTTCCAAATTCAGGAACTGGATGAACTGTTTACCCTCCTCTTTCTCTCTACATATACCATTCATCCCCGTCAGTAGACTTTACTATTTTTTCAATCCATATAACAGGAATTTCATCAATTCTTGGATCACTTAACTTTATTGTAACAATTTTTATAATAAAGAATTTTTCTTTAAAGTACGATCAAATTAATCTTTTTTCATGATCTATCTCAATTACTGTAATCTTACTAATCCTTTCTCTCCCAGTTCTAGCAGGGACAATTACTATGCTGTTATTTGACCGAAACTTTAACACTTCCTTCTTTGATCCAATAGGAGGGGGAGATCCAATTCTTTATCAACATCTATTTATCTGCCCAATGAATTT-----TTTTAATGGCTGCAGTATAACTGACTGTACAAAGGTAGCAT---CAATTGGTTTT---ATGAAATCTGGAATGAAAGGATTAATGAAATATGTACTGTCTCAATTATACAATTATGAAATTAAAATTTTAATAAAAATGTTAAAATTCACTTATGGGACGATAAGACCCTATAGAATTTTATATTGAAATTACTCAGTAGTTAATTCAGAAATAGTTTCAATATTTGATTGGGAGGATTAT---AAT---CCAACTT--TAATT---GTTAACTTTAATTTAAGA-GTAGATAATGATCTTCAATTTGAAATTGCTAGAATAAATTACCTTAGGGATAACAGCGTAATACTTTTTTATAGGCCATATAGAAAAAAGTGGTTGCGACCTCGATGTTGAATTAGGATAAATTTTAAATGCAGGAGTTTAATAATTAAGTCTGTCGTCGACGGCGTAGGCTACCGTTGGTGCGCGATGCTCCGGATGGACCCCC-GCGGTTCCAT---CGAGGGCACGCCACCCTCGGATCGAACGCTCCTGCGTCGT---CGTGCACTTCTCCCCTAGTAGAACGTCGCGACCCGTTGTGTGTCGGTCTACGGCCCGAGCGGGAGACTGTCGCGTCGCTTCGGCGCACGCGGCAGACCCTCGGTCGCCCGGCCGGCTGCACGACGGTACACTCACGGTATCGGGCCGCAGCCAATCCATT--CTCGAATGTGTGTGCGTCCATCCCGCCGCAAGCTCGGTCAGTTCTTACCCGGAGGCCACGGACCCAGTGCCGTCCCCGGGCCTGGCCAGCTGTTAGCGGGCGGTGTCCTCGGACCGGCCAAACCTCGGATTACCGGTCGGCGACGCTATTGCTTTGGGTACTCTCAGGACCCGTCTTGAAACACGGACCAAGGAGTCTAACATGTGCGCGAGTCATTGGGATGCATAAACCTAAAGGCGAAATGAAAGTGAAAGTCGGCCTTCGCGCCGATCGAGGGAGGATGGGCCGCGTAACAATGCGGCCCCGCACTCCCGGGGCGTCTCGTTCTCACTGCGAGAAGAGGCG

>HT32 KBKLC27

GGGATTCTGTATATAATTTTTGCTATCTGATCAGGTATTGCTGGTTCTTCCTTCAGAATACTTATTCGAATGGAACTTAATAGCCCTGGAACTTGAATTAGTAATGATCAGATCTACAACTCTATTGTAACTAGGCATGCATTTCTGATAATTTTTTTTATAGTAATACCTTTTATAATTGGAGGGTTTGGAAATTTTTTGATTCCGCTAATACTAGGATCTCCTGATATAACTTTCCCTCAAATAAACAATATTAGCTTTTGACTACTACCTCCTTCACTTATACTGCTAATAACATATAATTTATTTTTTCCAAATTCAGGAACTGGATGAACTGTTTACCCTCCTCTTTCTCTCTACATATACCATTCATCCCCGTCAGTAAACTTTACTATTTTTTCAATCCATATAACAGGAATTTCATCAATTCTTGGATCACTTAACTTTATTGTAACAATTTTTATAATAAAGAATTTTTCTTTAAAGTACGATCAAATTAATCTTTTTTCATGATCTATCTCAATTACTGTAATCTTACTAATCCTTTCTCTCCCATTTCTAGCAGGAGCAATTACTATGCTGTTATTTGACCGAAACTTTAACACTTCCTTCTTTGATCCTATAGGAGGGGGAGATCCAATTCTTTATCAACATCTATTTATCTGCCCAATGAATTT-----TTTTAATGGCTGCAGTATAACTGACTGTACAAAGGTAGCAT---CAATTGGTTTT---ATGAAATCTGGAATGAAAGGATTAATGAAATATGTACTGTCTCAATTATACAATTATGAAATTAAAATTTTAATAAAAATGTTAAAATTCACTTATGGGACGATAAGACCCTATAGAATTTTATATTGAAATTACTCAGTAGTTAATTCAGAAATAGTTTCAATATTTGATTGGGAGGATTAT---AAT---CCAACTT--TAATT---GTTAACTTTAATTTAAGA-GTAGATAATGATCTTCAATTTGAAATTGCTAGAATAAATTACCTTAGGGATAACAGCGTAATACTTTTTTATAGGCCATATAGAAAAAAGTGGTTGCGACCTCGATGTTGAATTAGGATAAATTTTAAATGCAGGAGTTTAATAATTAAGTCTGTCGTCGACGGCGTAGGCTACCGTTGGTGCGCGATGCTCCGGATGGACCCCC-GCGGTTCCAT---CGAGGGCACGCCACCCTCGGATCGAACGCTCCTGCGTCGT---CGTGCACTTCTCCCCTAGTAGAACGTCGCGACCCGTTGTGTGTCGGTCTACGGCCCGAGCGGGAGACTGTCGCGTCGCTTCGGCGCACGCGGCAGACCCTCGGTCGCCCGGCCGGCTGCACGACGGTACACTCACGGTATCGGGCCGCAGCCAATCCATT--CTCGAATGTGTGTGCGTCCATCCCGCCGCAAGCTCGGTCAGTTCTTACCCGGAGGCCACGGACCCAGTGCCGTCCCCGGGCCTGGCCAGCTGTTAGCGGGCGGTGTCCTCGGACCGGCCAAACCTCGGATTACCGGTCGGCGACGCTATTGCTTTGGGTACTCTCAGGACCCGTCTTGAAACACGGACCAAGGAGTCTAACATGTGCGCGAGTCATTGGGATGCATAAACCTAAAGGCGAAATGAAAGTGAAAGTCGGCCTTCGCGCCGATCGAGGGAGGATGGGCCGCGTAACAATGCGGCCCCGCACTCCCGGGGCGTCTCGTTCTCACTGCGAGAAGAGGCG

>HT33 KBKLC28

GGGATTCTGTATATAATTTTTGCTATCTGATCAGGTATTGCTGGTTCTTCCTTCAGAATACTTATTCGAATGGAACTTAATAGCCCTGGAACTTGAATTAGTAATGATCAGATCTACAACTCTATTGTAACTAGGCATGCATTTCTGATAATTTTTTTTATAGTAATACCTTTTATAATTGGAGGGTTTGGAAATTTTTTGATTCCGCTAATACTAGGATCTCCTGATATAGCTTTCCCTCGAATAAACAATATTAGCTTTTGACTACTACCTCCTTCACTTATACTGCTAATAACAAATAATTTATTTTTTCCAAATTCAGGAACTGGATGAACTGTATACCCTCCTCTTTCTCTCTACATATACCATTCATCCCCGTCAGTAGACTTTACTATTTTTTCAATCCATATAACAGGAATTTCATCAATTCTTGGATCACTTAACTTTATTGTAACAATTTTTATAATAAAGAATTTTTCTTTAAAGTACGATCAAATTAATCTTTTTTCATGATCTATCTCAATTACTGTAATCTTACTAATCCTTTCTCTCCCAGTTCTAGCAGGAGCAATTACTATGCTGTTATTTGACCGAAATTTTAACACCTCCTTCTTTGATCCAATAGGAGGGGGAGATCCAATTCTTTATCAACATCTATTTATCTGCCCAATGAATTT-----TTTTAATGGCTGCAGTATAACTGACTGTACAAAGGTAGCAT---CAATTGGTTTT---ATGAAATCTGGAATGAAAGGATTAATGAAATATGTACTGTCTCAATTGTACAATTATGAAATTAAAATTTTAATAAAAATGTTAAAATTCACTTATGGGACGATAAGACCCTATAGAATTTTATATTGAAATTACTCAGTAGTTAATTCAGAAATAGTTTCAATATTTGATTGGGAGGATTAT---AAT---CCAACTT--TAATT---GTTAACTTTAATTTAAGA-GTAGATAATGATCTTCAATTTGAAATTGCTAGAATAAATTACCTTAGGGATAACAGCGTAATACTTTTTTATAGGCCATATAGAAAAAAGTGGTTGCGACCTCGATGTTGAATTAGGATAAATTTTAAATGCAGGAGTTTAATAATTAAGTCTGTCGTCGACGGCGTAGGCTACCGTTGGTGCGCGATGCTCCGGATGGACCCCC-GCGGTTCCAT---CGAGGGCACGCCACCCTCGGATCGAACGCTCCTGCGTCGT---CGTGCACTTCTCCCCTAGTAGAACGTCGCGACCCGTTGTGTGTCGGTCTACGGCCCGAGCGGGAGACTGTCGCGTCGCTTCGGCGCACGCGGCAGACCCTCGGTCGCCCGGCCGGCTGCACGACGGTACACTCACGGTATCGGGCCGCAGCCAATCCATT--CTCGAATGTGTGTGCGTCCATCCCGCCGCAAGCTCGGTCAGTTCTTACCCGGAGGCCACGGACCCAGTGCCGTCCCCGGGCCTGGCCAGCTGTTAGCGGGCGGTGTCCTCGGACCGGCCAAACCTCGGATTACCGGTCGGCGACGCTATTGCTTTGGGTACTCTCAGGACCCGTCTTGAAACACGGACCAAGGAGTCTAACATGTGCGCGAGTCATTGGGATGCATAAACCTAAAGGCGAAATGAAAGTGAAAGTCGGCCTTCGCGCCGATCGAGGGAGGATGGGCCGCGTAACAATGCGGCCCCGCACTCCCGGGGCGTCTCGTTCTCACTGCGAGAAGAGGCG

>HT34 NSTMNC29

GGAATTCTGTATATAATTTTTGCTATCTGATCAGGTATTGTTGGTTCTTCCTTCAGAATACTTATTCGAATGGAACTTAATAGCCCTGGAACTTGAATTAGTAATGATCAGATCTACAACTCTATTGTGACTAGGCATGCATTTCTAATAATTTTTTTTATAGTAATACCTTTTATAATCGGAGGTTTTGCAAATTTTTTAATTCCGCTAATACTAGGTTCTCCTGATATAACTTTCCCCCGAATGAACAATATCAGCTTCTGACTACTACCTCCTTCACTTATACTGCTAATAACAAATAATTTATTCTTTCCGAATTCAGGAACTGGATGAACTGTATACCCCCCTCTTTCTCTCTACATATATCATTCATCTCCGTCAGTGGACTTTACTATTTTTTCAATTCATATAACGGGAATTTCATCAATTCTTGGGTCACTTAACTTTATTGTAACAATTTTTATAATAAAGAACTTTTCTTTGAAGTACGACCAAATTAATCTTTTTTCATGATCTATCTCAATTACTGTAATTTTACTAATTCTTTCTCTTCCAGTTCTAGCAGGAGCAATTACTATGCTGTTATTTGATCGAAATTTTAATACTTCCTTCTTTGATCCAATAGGAGGGGGAGACCCGATTCTTTATCAACATTTATTTATCTGCCCAATGAATTC-----TTTTAATGGCTGCAGTATAACTGACTGTACAAAGGTAGCAT---CAATTGGTTTT---ATGAAATCTGGAATGAAAGGATTAATGAAATATGTACTGTCTCAATTGTACAATTATGAAATTAAAATTTTAATAAAAATGTTAAAATTCACTTATGGGACGATAAGACCCTATAGAATTTTATATTGAAATTACTCAGTAGTTAATTCAGAAATAGTTTCAATATTTGATTGGGAGGATTAT---ACT---CCAACTT--TAATT---GTTAACTTTAATTTAAGA-GTAGATAATGATCTTCAATTTGAAATTGCTAGAATAAATTACCTTAGGGATAACAGCGTAATACTTTTTTATAGGCCATATAGAAAAAAGTGGTTGCGACCTCGATGTTGAATTAGGATAAATTTTAAATGCAGGAGTTTAATAATTAAGTCTGTCGTCGACGGCGTAGGCTACCGTTGGTGCGCGATGCTCCGGATGGACCCCC-GCGGTTCCAT---CGAGGGCACGCCACCCTCGGATCGAACGCTCCTGCGTCGT---CGTGCACTTCTCCCCTAGTAGAACGTCGCGACCCGTTGTGTGTCGGTCTACGGCCCGAGCGGGAGACTGTCGCGTCGCTTCGGCGCACGCGGCAGACCCTCGGTCGCCCGGCCGGCTGCACGACGGTACACTCACGGTATCGGGCCGCAGCCAATCCATT--CTCGAATGTGTGTGCGTCCATCCCGCCGCAAGCTCGGTCAGTTCTTACCCGGAGGCCACGGACCCAGTGCCGTCCCCGGGCCTGGCCAGCTGTTAGCGGGCGGTGTCCTCGGACCGGCCAAACCTCGGATTACCGGTCGGCGACGCTATTGCTTTGGGTACTCTCAGGACCCGTCTTGAAACACGGACCAAGGAGTCTAACATGTGCGCGAGTCATTGGGATGCATAAACCTAAAGGCGAAATGAAAGTGAAAGTCGGCCTTCGCGCCGATCGAGGGAGGATGGGCCGCGTAACAATGCGGCCCCGCACTCCCGGGGCGTCTCGTTCTCACTGCGAGAAGAGGCG

>HT35 NSTMNC30

GGGATTCTGTATATAATTTTTGCTATCTGATCAGGTATTGCTGGTTCTTCCTTCAGAATACTTATTCGAATGGAACTTAATAGCCCTGGAACTTGAATTAGTAATGATCAGATCTACAACTCTATTGTAACTAGGCATGCATTTCTGATAATTTTTTTTATAGTAATACCTTTTATAATTGGAGGGTTTGGAAATTTTTTGATTCCGCTAATACTAGGATCTCCTGATATAGCTTTCCCTCGAATAAACAATATTAGCTTTTGACTACTACCTCCTTCACTTATACTGCTAATAACAAATAATTTATTTTTTCCAAATTCAGGAACTGGATGAACTGTATACCCTCCTCTTTCTCTCTACATATACCATTCATCCCCGTCAGTAGACTTTACTATTTTTTCAATCCATATAACAGGAATCTCATCAATTCTTGGATCACTTAACTTTATTGTAACAATTTTTATAATAAAGAATTTTTCTTTAAAGTACGATCAAATTAATCTTTTTTCATGATCTATCTCAATTACTGTAATTTTACTAATCCTTTCTCTCCCAGTTCTAGCAGGAGCAATTACTATGCTGTTATTTGACCGAAATTTTAACACTTCCTTCTTTGATCCAATAGGAGGGGGAGATCCAATTCTTTATCAACATCTATTTATCTGCCCAATGAATTT-----TTTTAATGGCTGCAGTATAACTGACTGTACAAAGGTAGCAT---CAATTGGTTTT---ATGAAATCTGGAATGAAAGGATTAATGAAATATGTACTGTCTCAATTGTACAATTATGAAATTAAAATTTTAATAAAAATGTTAAAATTCACTTATGGGACGATAAGACCCTATAGAATTTTATATTGAAATTACTCAGTAGTTAATTCAGAAATAGTTTCAATATTTGATTGGGAGGATTAT---AAT---CCAACTT--TAATT---GTTAACTTTAATTTAAGA-GTAGATAATGATCTTCAATTTGAAATTGCTAGAATAAATTACCTTAGGGATAACAGCGTAATACTTTTTTATAGGCCATATAGAAAAAAGTGGTTGCGACCTCGATGTTGAATTAGGATAAATTTTAAATGCAGGAGTTTAATAATTAAGTCTGTCGTCGACGGCGTAGGCTACCGTTGGTGCGCGATGCTCCGGATGGACCCCC-GCGGTTCCAT---CGAGGGCACGCCACCCTCGGATCGAACGCTCCTGCGTCGT---CGTGCACTTCTCCCCTAGTAGAACGTCGCGACCCGTTGTGTGTCGGTCTACGGCCCGAGCGGGAGACTGTCGCGTCGCTTCGGCGCACGCGGCAGACCCTCGGTCGCCCGGCCGGCTGCACGACGGTACACTCACGGTATCGGGCCGCAGCCAATCCATT--CTCGAATGTGTGTGCGTCCATCCCGCCGCAAGCTCGGTCAGTTCTTACCCGGAGGCCACGGACCCAGTGCCGTCCCCGGGCCTGGCCAGCTGTTAGCGGGCGGTGTCCTCGGACCGGCCAAACCTCGGATTACCGGTCGGCGACGCTATTGCTTTGGGTACTCTCAGGACCCGTCTTGAAACACGGACCAAGGAGTCTAACATGTGCGCGAGTCATTGGGATGCATAAACCTAAAGGCGAAATGAAAGTGAAAGTCGGCCTTCGCGCCGATCGAGGGAGGATGGGCCGCGTAACAATGCGGCCCCGCACTCCCGGGGCGTCTCGTTCTCACTGCGAGAAGAGGCG

>HT36 NSTMNC31

GGGATTCTGTATATAATTTTTGCTATCTGATCAGGTATTGCTGGTTCTTCCTTCAGAATACTTATTCGAATGGAACTTAATAGCCCTGGAACTTGAATTAGTAATGATCAGATCTACAACTCTATTGTAACTAGGCATGCATTTCTGATAATTTTTTTTATAGTAATACCTTTTATAATTGGAGGGTTTGGAAATTTTTTGATTCCGCTAATACTAGGATCTCCTGATATAGCTTTCCCTCGAATAAACAATATTAGCTTTTGACTACTACCTCCTTCACTTATACTGCTAATAACAAATAATTTATTTTTTCCAAATTCAGGAACTGGATGAACTGTATACCCTCCTCTTTCTCTCTACATATACCATTCATCCCCGTCAGTAGACTTTACTATTTTTTCAATCCATATAACAGGAATTTCATCAATTCTTGGATCACTTAACTTTATTGTAACAATTTTTATAATAAAGAATTTTTCTTTAAAGTACGATCAAATTAATCTTTTTTCATGATCTATCTCAATTACTGTAATCTTACTAATCCTTTCTCTCCCAGTTCTAGCAGGGACAATTACTATGCTGTTATTTGACCGAAATTTTAACACTTCCTTCTTTGATCCAATAGGAGGGGGAGATCCAATTCTTTATCAACATCTATTTATCTGCCCAATGAATTT-----TTTTAATGGCTGCAGTATAACTGACTGTACAAAGGTAGCAT---CAATTGGTTTT---ATGAAATCTGGAATGAAAGGATTAATGAAATATGTACTGTCTCAATTGTACAATTATGAAATTAAAATTTTAATAAAAATGTTAAAATTCACTTATGGGACGATAAGACCCTATAGAATTTTATATTGAAATTACTCAGTAGTTAATTCAGAAATAGTTTCAATATTTGATTGGGAGGATTAT---AAT---CCAACTT--TAATT---GTTAACTTTAATTTAAGA-GTAGATAATGATCTTCAATTTGAAATTGCTAGAATAAATTACCTTAGGGATAACAGCGTAATACTTTTTTATAGGCCATATAGAAAAAAGTGGTTGCGACCTCGATGTTGAATTAGGATAAATTTTAAATGCAGGAGTTTAATAATTAAGTCTGTCGTCGACGGCGTAGGCTACCGTTGGTGCGCGATGCTCCGGATGGACCCCC-GCGGTTCCAT---CGAGGGCACGCCACCCTCGGATCGAACGCTCCTGCGTCGT---CGTGCACTTCTCCCCTAGTAGAACGTCGCGACCCGTTGTGTGTCGGTCTACGGCCCGAGCGGGAGACTGTCGCGTCGCTTCGGCGCACGCGGCAGACCCTCGGTCGCCCGGCCGGCTGCACGACGGTACACTCACGGTATCGGGCCGCAGCCAATCCATT--CTCGAATGTGTGTGCGTCCATCCCGCCGCAAGCTCGGTCAGTTCTTACCCGGAGGCCACGGACCCAGTGCCGTCCCCGGGCCTGGCCAGCTGTTAGCGGGCGGTGTCCTCGGACCGGCCAAACCTCGGATTACCGGTCGGCGACGCTATTGCTTTGGGTACTCTCAGGACCCGTCTTGAAACACGGACCAAGGAGTCTAACATGTGCGCGAGTCATTGGGATGCATAAACCTAAAGGCGAAATGAAAGTGAAAGTCGGCCTTCGCGCCGATCGGGGGAGGATGGGCCGCGTAACAATGCGGCCCCGCACTCCCGGGGCGTCTCGTTCTCACTGCGAGAAGAGGCG

>HT39 NSTLSC32

GGGATTCTGTATATAATTTTTGCTATCTGATCAGGTATTGCTGGTTCTTCCTTCAGAATACTTATTCGAATGGAACTTAATAGCCCTGGAACTTGAATTAGTAATGATCAGATCTACAACTCTATTGTAACTAGGCATGCATTTCTGATAATTTTTTTTATAGTAATACCTTTTATAATTGGAGGGTTTGGAAATTTTTTGATTCCACTAATACTAGGATCTCCTGATATAGCTTTTCCTCGAATAAACAATATTAGCTTTTGACTTCTACCTCCCTCACTTATACTTCTAATAACAAATAATTTATTTTTTCCAAATTCAGGAACTGGATGAACTGTGTACCCTCCTCTTTCTCTCTACATATACCATTCATCCCCTTCAGTAGACTTTACTATTTTTTCAATCCATATAACAGGAATTTCATCAATTCTTGGATCACTTAACTTTATTGTAACAATTTTTATAATAAAGAATTTTTCTTTAAAGTACGATCAAATTAATCTTTTTTCATGATCTATCTCAATTACTGTAATTTTACTAATCCTTTCTCTCCCAGTTCTAGCGGGAGCAATTACTATGCTGTTATTTGACCGAAATTTTAACACCTCCTTCTTTGATCCAATAGGAGGGGGAGACCCAATTCTTTATCAGCATCTATTTATCTGCCCAATGAATTT-----TTTTAATGGCTGCAGTATAACTGACTGTACAAAGGTAGCAT---CAATTGGTTTT---ATGAAATCTGGAATGAAAGGATTAATGAAATATGTACTGTCTCAATTGTACAATTATGAAATTAAAATTTTAATAAAAATGTTAAAATTCACTTATGGGACGATAAGACCCTATAGAATTTTATATTGAAATTACTCAGTAGTTAATTCAGAAATAGTTTCAATATTTGATTGGGAGGATTAT---AAT---CCAACTT--TAATT---GTTAACTTTAATTTAAGA-GTAGATAATGATCTTCAATTTGAAATTGCTAGAATAAATTACCTTAGGGATAACAGCGTAATACTTTTTTATAGGCCATATAGAAAAAAGTGGTTGCGACCTCGATGTTGAATTAGGATAAATTTTAAATGCAGGAGTTTAATAATTAAGTCTGTCGTCGACGGCGTAGGCTACCGTTGGTGCGCGATGCTCCGGATGGACCCCC-GCGGTTCCAT---CGAGGGCACGCCACCCTCGGATCGAACGCTCCTGCGTCGT---CGTGCACTTCTCCCCTAGTAGAACGTCGCGACCCGTTGTGTGTCGGTCTACGGCCCGAGCGGGAGACTGTCGCGTCGCTTCGGCGCACGCGGCAGACCCTCGGTCGCCCGGCCGGCTGCACGACGGTACACTCACGGTATCGGGCCGCAGCCAATCCATT--CTCGAATGTGTGTGCGTCCATCCCGCCGCAAGCTCGGTCAGTTCTTACCCGGAGGCCACGGACCCAGTGCCGTCCCCGGGCCTGGCCAGCTGTTAGCGGGCGGTGTCCTCGGACCGGCCAAACCTCGGATTACCGGTCGGCGACGCTATTGCTTTGGGTACTCTCAGGACCCGTCTTGAAACACGGACCAAGGAGTCTAACATGTGCGCGAGTCATTGGGATGCATAAACCTAAAGGCGAAATGAAAGTGAAAGTCGGCCTTCGCGCCGATCGAGGGAGGATGGGCCGCGTAACAATGCGGCCCCGCACTCCCGGGGCGTCTCGTTCTCACTGCGAGAAGAGGCG

>HT40 NSTLSC33

GGAATTCTTTATATAATTTTTGCCATTTGATCTGGCATTGTAGGTTCATCTTTTAGAATACTTATTCGGATGGAGCTTAATAGTCCTGGAACTTGAATTAATAATGATCAAATTTACAATTCTATTGTAACTAGTCATGCATTTCTAATAATTTTCTTTATAGTTATACCCTTTATAATTGGAGGGTTCGGGAACTTTCTAATTCCACTAATAATGGGTTCCCCTGATATAGCCTTTCCTCGAATAAATAACGTTAGATTTTGATTACTTCCACCCTCAATTATACTACTTATAACAAATAATCTATTTTATCCTAATTCAGGAACTGGATGAACTGTATATCCTCCACTTTCTCTTTATATATATCATTCATCTCCATCTGTAGATTTCACTATTTTTTCAATTCATATAACAGGAATTTCATCAATTCTAGGATCTCTTAATTTTATTGTTACAATTTTTATAATAAAAAATTTCTCTTTGAAGTATGATCAAATTAATCTATTTTCATGATCTATCTCAATTACTGTAATCTTACTAATTCTTTCCCTTCCGGTTCTAGCGGGAGCAATTACTATACTTTTATTTGACCGAAACTTCAACACCTCATTTTTTGATCCAATAGGAGGAGGAGATCCTATTCTATACCAACATCTGTTC-------------------------------------------------------------------------------------------------------------------------------------------------------------------------------------------------------------------------------------------------------------------------------------------------------------------------------------------------------------------------------------------------------------------------------------------------------------------------------CGTCGACGGCGTAGGCTACCGTTGGTGCGCGATGCTCCGGATGGACCCCC-GCGGTTCCAT---CGAGGGCACGCCACCCTCGGATCGAACGCTCCTGCGTCGT---CGTGCACTTCTCCCCTAGTAGAACGTCGCGACCCGTTGTGTGTCGGTCTACGGCCCGAGCGGGAGACTGTCGCGTCGCTTCGGCGCACGCGGCAGACCCTCGGTCGCCCGGCCGGCTGCACGACGGTACACTCACGGTATCGGGCCGCAGCCAATCCATT--CTCGAATGTGTGTGCGTCCATCCCGCCGCAAGCTCGGTCAGTTCTTACCCGGAGGCCACGGACCCAGTGCCGTCCCCGGGCCTGGCCAGCTGTTAGCGGGCGGTGTCCTCGGACCGGCCAAACCTCGGATTACCGGTCGGCGACGCTATTGCTTTGGGTACTCTCAGGACCCGTCTTGAAACACGGACCAAGGAGTCTAACATGTGCGCGAGTCATTGGGATGCATAAACCTAAAGGCGAAATGAAAGTGAAAGTCGGCCTTCGCGCCGATCGAGGGAGGATGGGCCGCGTAACAATGCGGCCCCGCACTCCCGGGGCGTCTCGTTCTCACTGCGAGAAGAGGCG

>HT44 NSTMNC35

GGGATTCTGTATATAATTTTTGCTATCTGATCAGGTATTGCTGGTTCTTCCTTCAGAATACTTATTCGAATGGAACTTAATAGCCCTGGAACTTGAATTAGTAATGATCAGATCTACAACTCTATTGTGACTAGGCATGCATTTCTAATAATTTTTTTTATAGTAATACCTTTTATAATCGGAGGTTTTGGAAATTTTTTAATTCCGCTAATACTAGGTTCTCCTGATATAACTTTCCCCCAAATGAACAATATCAACTTCTGACTACTACCTCCTTCACTTATACTGCTAATAACAAATAATTTATTCTTTCCGAATTCAGGAACTGGATGAACTGTATACCCCCCTCTTTCTCTCTACATATATCATTCATCTCCGTCAGTGGACTTTACTATTTTTTCAATTCATATAACGGGAATTTCATCAATTCTTGGGTCACTTAACTTTATTGTAACAATTTTTATAATAAAGAACTTTTCTTTGAAGTACGACCAAATTAATCTTTTTTCATGATCTATCTCAATTACTGTAATTTTACTAATTCTTTCTCTTCCAGTTCTAGCAGGAGCAATTACTATGCTGTTATTTGATCGAAATTTTAATACTTCCTTCTTTGATCCAATAGGAGGGGGAGACCCGATTCTTTATCAACATTTATTTATCTGCCCAATGAATTC-----TTTTAATGGCTGCAGTATAACTGACTGTACAAAGGTAGCAT---CAATTGGTTTT---ATGAAATCTGGAATGAAAGGATTAATGAAATATGTACTGTCTCAATTGTACAATTATGAAATTAAAATTTTAATAAAAATGTTAAAATTCACTTATGGGACGATAAGACCCTATAGAATTTTATATTGAAATTACTCAGTAGTTAATTCAGAAATAGTTTCAATATTTGATTGGGAGGATTAT---ACT---CCAACTT--TAATT---GTTAACTTTAATTTAAGA-GTAGATAATGATCTTCAATTTGAAATTGCTAGAATAAATTACCTTAGGGATAACAGCGTAATACTTTTTTATAGGCCATATAGAAAAAAGTGGTTGCGACCTCGATGTTGAATTAGGATAAATTTTAAATGCAGGAGTTTAATAATTAAGTCTGTCGTCGACGGCGTAGGCTACCGTTGGTGCGCGATGCTCCGGATGGACCCCC-GCGGTTCCAT---CGAGGGCACGCCACCCTCGGATCGAACGCTCCTGCGTCGT---CGTGCACTTCTCCCCTAGTAGAACGTCGCGACCCGTTGTGTGTCGGTCTACGGCCCGAGCGGGAGGCTGTCGCGTCGCTTCGGCGCACGCGGCAGACCCTCGGTCGCCCGGCCGGCTGCACGACGGTACACTCACGGTATCGGGCCGCAGCCAATCCATT--CTCGAATGTGTGTGCGTCCATCCCGCCGCAAGCTCGGTCAGTTCTTACCCGGAGGCCACGGACCCAGTGCCGTCCCCGGGCCTGGCCAGCTGTTAGCGGGCGGTGTCCTCGGACCGGCCAAACCTCGGATTACCGGTCGGCGACGCTATTGCTTTGGGTACTCTCAGGACCCGTCTTGAAACACGGACCAAGGAGTCTAACATGTGCGCGAGTCATTGGGATGCATAAACCTAAAGGCGAAATGAAAGTGAAAGTCGGCCTTCGCGCCGATCGAGGGAGGATGGGCCGCGTAACAATGCGGCCCCGCACTCCCGGGGCGTCTCGTTCTCACTGCGAGAAGAGGCG

>HT45 NSTMNC36

GGAATTCTGTATATAATTTTTGCTATCTGATCAGGTATTGTTGGTTCTTCCTTCAGAATACTTATTCGAATGGAACTTAATAGCCCTGGAACTTGAATTAGTAATGATCAGATCTACAACTCTATTGTGACTAGGCATGCATTTCTAATAATTTTTTTTATAGTAATACCTTTTATAATCGGAGGTTTTGGAAATTTTTTAATTCCGCTAATACTAGGTTCTCCTGATATAGCTTTCCCCCGAATGAACAATATCAGCTTCTGACTACTACCTCCTTCACTTATACTGCTAATAACAAATAATTTATTCTTTCCGAATTCAGGAACTGGATGAACTGTATACCCCCCTCTTTCTCTCTACATATATCATTCATCTCCGTCAGTGGACTTTACTATTTTTTCAATTCATATAACGGGAATTTCATCAATTCTTGGGTCACTTAACTTTATTGTAACAATTTTTATAATAAAGAACTTTTCTTTGAAGTACGACCAAATTAATCTTTTTTCATGATCTATCTCGATTACTGTAATTTTACTAATTCTTTCTCTTCCAGTTCTAGCAGGAGCAATTACTATGCTGTTATTTGATCGAAATTTTAATACTTCCTTCTTTGATCCAATAGGAGGGGGAGACCCGATTCTTTATCAACATTTATTTATCTGCCCAATGAATTT-----TTTTAATGGCTGCAGTATAACTGACTGTACAAAGGTAGCAT---CAATTGGTTTT---ATGAAATCTGGAATGAAAGGATTAATGAAATATGTACTGTCTCAATTGTACAATTATGAAATTAAAATTTTAATAAAAATGTTAAAATTCACTTATGGGACGATAAGACCCTATAGAATTTTATATTGAAATTACTCAGTAGTTAATTCAGAAATAGTTTCAATATTTGATTGGGAGGATTAT---ACT---CCAACTT--TAATT---GTTAACTTTAATTTAAGA-GTAGATAATGATCTTCAATTTGAAATTGCTAGAATAAATTACCTTAGGGATAACAGCGTAATACTTTTTTATAGGCCATATAGAAAAAAGTGGTTGCGACCTCGATGTTGAATTAGGATAAATTTTAAATGCAGGAGTTTAATAATTAAGTCTGTCGTCGACGGCGTAGGCTACCGTTGGTGCGCGATGCTCCGGATGGACCCCC-GCGGTTCCAT---CGAGGGCACGCCACCCTCGGATCGAACGCTCCTGCGTCGT---CGTGCACTTCTCCCCTAGTAGAACGTCGCGACCCGTTGTGTGTCGGTCTACGGCCCGAGCGGGAGACTGTCGCGTCGCTTCGGCGCACGCGGCAGACCCTCGGTCGCCCGGCCGGCTGCACGACGGTACACTCACGGTATCGGGCCGCAGCCAATCCATT--CTCGAATGTGTGTGCGTCCATCCCGCCGCAAGCTCGGTCAGTTCTTACCCGGAGGCCACGGACCCAGTGCCGTCCCCGGGCCTGGCCAGCTGTTAGCGGGCGGTGTCCTCGGACCGGCCAAACCTCGGATTACCGGTCGGCGACGCTATTGCTTTGGGTACTCTCAGGACCCGTCTTGAAACACGGACCAAGGAGTCTAACATGTGCGCGAGTCATTGGGATGCATAAACCTAAAGGCGAAATGAAAGTGAAAGTCGGCCTTCGCGCCGATCGGGGGAGGATGGGCCGCGTAACAATGCGGCCCCGCACTCCCGGGGCGTCTCGTTCTCACTGCGAGAAGAGGCG

>HT46 NSTMNC37

GGAATTCTGTATATAATTTTTGCTATCTGATCAGGTATTGTTGGTTCTTCCTTCAGAATACTTATTCGAATGGAACTTAATAGCCCTGGAACTTGAATTAGTAATGATCAGATCTACAACTCTATTGTGACTAGTCATGCATTTCTAATAATTTTTTTTATAGTAATACCTTTTATAATCGGAGGCTTTGGAAATTTTTTAATCCCGTTAATACTAGGTTCCCCTGATATAGCTTTCCCTCGAATAAACAATATTAGCTTCTGACTACTACCTCCTTCACTTATACTGCTAATAACAAATAATCTATTTTTTCCGAACTCAGGAACTGGATGAACTGTATACCCTCCTCTTTCTCTTTACATATATCATCCATCCCCGTCAGTGGACTTTACTATTTTTTCAATCCATATAACAGGAATTTCATCAATTCTTGGGTCACTTAACTTTATTGTAACAATTTTTATAATAAAGAACTTTTCTTTAAAGTACGATCAAATTAATCTTTTTTCATGATCTATCTCAATTACTGTAATTTTACTAATTCTTTCTCTTCCAGTTCTAGCAGGAGCAATTACTATGCTGTTATTTGATCGAAATTTTAATACTTCCTTCTTTGATCCAATAGGAGGGGGAGACCCGATTCTTTATCAACATTTATTTATCTGCCCAATGAATTT-----TTTTAATGGCTGCAGTATAACTGACTGTACAAAGGTAGCAT---CAATTGGTTTT---ATGAAATCTGGAATGAAAGGATTAATGAAATATGTACTGTCTCAATTGTACAATTATGAAATTAAAATTTTAATAAAAATGTTAAAATTCACTTATGGGACGATAAGACCCTATAGAATTTTATATTGAAATTACTCAGTAGTTAATTCAGAAATAGTTTCAATATTTGATTGGGAGGATTAT---ACT---CCAACTT--TAATT---GTTAACTTTAATTTAAGA-GTAGATAATGATCTTCAATTTGAAATTGCTAGAATAAATTACCTTAGGGATAACAGCGTAATACTTTTTTATAGGCCATATAGAAAAAAGTGGTTGCGACCTCGATGTTGAATTAGGATAAATTTTAAATGCAGGAGTTTAATAATTAAGTCTGTCGTCGACGGCGTAGGCTACCGTTGGTGCGCGATGCTCCGGATGGACCCCC-GCGGTTCCAT---CGAGGGCACGCCACCCTCGGATCGAACGCTCCTGCGTCGT---CGTGCACTTCTCCCCTAGTAGAACGTCGCGACCCGTTGTGTGTCGGTCTACGGCCCGAGCGGGAGGCTGTCGCGTCGCTTCGGCGCACGCGGCAGACCCTCGGTCGCCCGGCCGGCTGCACGACGGTACACTCACGGTATCGGGCCGCAGCCAATCCATT--CTCGAATGTGTGTGCGTCCATCCCGCCGCAAGCTCGGTCAGTTCTTACCCGGAGGCCACGGACCCAGTGCCGTCCCCGGGCCTGGCCAGCTGTTAGCGGGCGGTGTCCTCGGACCGGCCAAACCTCGGATTACCGGTCGGCGACGCTATTGCTTTGGGTACTCTCAGGACCCGTCTTGAAACACGGACCAAGGAGTCTAACATGTGCGCGAGTCATTGGGATGCATAAACCTAAAGGCGAAATGAAAGTGAAAGTCGGCCTTCGCGCCGATCGAGGGAGGATGGGCCGCGTAACAATGCGGCCCCGCACTCCCGGGGCGTCTCGTTCTCACTGCGAGAAGAGGCG

>HT49 NSTCHUN4

GGAATTCTGTATATAATTTTTGCTATCTGATCAGGTATTGTTGGTTCTTCCTTCAGAATACTTATTCGAATGGAACTTAATAGCCCTGGAACTTGAATTAGTAATGATCAGATCTACAACTCTATTGTGACTAGTCATGCATTTCTAATAATTTTTTTTATAGTAATACCTTTTATAATCGGAGGCTTTGGAAATTTTTTAATCCCGTTAATACTAGGTTCCCCTGATATAGCTTTCCCTCGAATAAACAATATTAGCTTCTGACTACTACCTCCTTCACTTATACTGCTAATAACAAATAATCTATTTTTTCCGAACTCAGGAACTGGATGAACTGTATACCCTCCTCTTTCTCTTTACATATATCATCCATCTCCGTCAGTGGACTTTACTATTTTTTCAATTCATATAACAGGAATTTCATCAATTCTTGGGTCACTTAACTTTATTGTAACAATTTTTATAATAAAGAACTTTTCTTTAAAGTACGATCAAATTAATCTTTTTTCATGATCTATCTCAATTACTGTAATTTTACTAATTCTTTCTCTTCCAGTTCTAGCAGGAGCAATTACTATGCTGTTATTTGATCGAAATTTTAATACTTCCTTCTTTGATCCAATAGGAGGGGGAGACCCGATTCTTTATCAACATTTATTTATCTGCCCAATGAATTT-----TTTTAATGGCTGCAGTATAACTGACTGTACAAAGGTAGCAT---CAATTGGTTTT---ATGAAATCTGGAATGAAAGGATTAATGAAATATGTACTGTCTCAATTGTACAATTATGAAATTAAAATTTTAATAAAAATGTTAAAATTCACTTATGGGACGATAAGACCCTATAGAATTTTATATTGAAATTACTCAGTAGTTAATTCAGAAATAGTTTCAATATTTGATTGGGAGGATTAT---ACT---CCAACTT--TAATT---GTTAACTTTAATTTAAGA-GTAGATAATGATCTTCAATTTGAAATTGCTAGAATAAATTACCTTAGGGATAACAGCGTAATACTTTTTTATAGGCCATATAGAAAAAAGTGGTTGCGACCTCGATGTTGAATTAGGATAAATTTTAAATGCAGGAGTTTAATAATTAAGTCTGTCGTCGACGGCGTAGGCTACCGTTGGTGCGCGATGCTCCGGATGGACCCCC-GCGGTTCCAT---CGAGGGCACGCCACCCTCGGATCGAACGCTCCTGCGTCGT---CGTGCACTTCTCCCCTAGTAGAACGTCGCGACCCGTTGTGTGTCGGTCTACGGCCCGAGCGGGAGACTGTCGCGTCGCTTCGGCGCACGCGGCAGACCCTCGGTCGCCCGGCCGGCTGCACGACGGTACACTCACGGTATCGGGCCGCAGCCAATCCATT--CTCGAATGTGTGTGCGTCCATCCCGCCGCAAGCTCGGTCAGTTCTTACCCGGAGGCCACGGACCCAGTGCCGTCCCCGGGCCTGGCCAGCTGTTAGCGGGCGGTGTCCTCGGACCGGCCAAACCTCGGATTACCGGTCGGCGACGCTATTGCTTTGGGTACTCTCAGGACCCGTCTTGAAACACGGACCAAGGAGTCTAACATGTGCGCGAGTCATTGGGATGCATAAACCTAAAGGCGAAATGAAAGTGAAAGTCGGCCTTCGCGCCGATCGAGGGAGGATGGGCCGCGTAACAATGCGGCCCCGCACTCCCGGGGCGTCTCGTTCTCACTGCGAGAAGAGGCG

>HT50 NSTCHUN5

GGAATTCTGTATATAATTTTTGCTATCTGATCAGGTATTGTTGGTTCTTCCTTCAGAATACTTATTCGAATGGAGCTTAATAGTCCTGGAACTTGAATTAATAATGATCAAATTTACAATTCTATTGTAACTAGTCATGCATTTCTAATAATTTTCTTTATAGTTATACCCTTTATAATTGGAGGGTTCGGGAACTTTCTAATTCCACTAATAATGGGTTCCCCTGATATAGCCTTTCCTCGAATAAATAACGTTAGATTTTGATTACTTCCACCCTCAATTATACTACTTATAACAAATAATCTATTTTATCCTAATTCAGGAACTGGATGAACTGTATATCCTCCACTTTCTCTTTATATATATCATTCATCTCCATCTGGAGATTTCACTATTTTTTCAATTCATATAACAGGAATTTCATCAATTCTAGGATCGCTTAATTTTATTGTTACAATTTTTATAATAAAAAATTTCTCTTTGAAGTATGATCAAATTAATCTATTTTCATGATCTATCTCAATTACTGTAATCTTACTAATTCTTTCCCTTCCGGTTCTAGCGGGAGCAATTACTATACTTTTATTTGACCGAAACTTCAACACCTCATTTTTTGATCCAATAGGAGGAGGAGATCCTATTCTATACCAACATCTGTTCATCTGCCCAATGAAGTT-----CTTTAATGGCTGCAGTATAACTGACTGTACAAAGGTAGCAT---CAATTGGTTTT---ATGAAATCTGGAATGAAAGGATTAATGAAATATGTACTGTCTCAATTGTATAATTATGAAATTAAAATTTTAATAAAAATGTTAAAATTTACTTATGGGACGATAAGACCCTATAGAATTTTATATTGAAATTACTCAGTAGTTAGTACAGAAATAATTTTAATATTTGGTTGGGAGGACTAT---ATT---CTAACTT--TAATT---ATTAACTTTAATTTAAGA-GTAAATAAGGATCTTCAATTTGAAATTATCAGAGTAAATTACCTTAGGGATAACAGCGTAATACTTTTTTATAGGCCATATAGAAAAAAGTGATTGCGACCTCGATGTTGAATTAGGATAAATTTTAAATGCAGGAGTTTAATAATTAAGTCTGTCGTCGACGGCGTAGGCTACCGTTGGTGCGCGATGCTCCGGATGGACCCCC-GCGGTTCCAT---CGAGGGCACGCCACCCTCGGATCGAACGCTCCTGCGTCGT---CGTGCACTTCTCCCCTAGTAGAACGTCGCGACCCGTTGTGTGTCGGTCTACGGCCCGAGCGGGAGACTGTCGCGTCGCTTCGGCGCACGCGGCAGACCCTCGGTCGCCCGGCCGGCTGCACGACGGTACACTCACGGTATCGGGCCGCAGCCAATCCATT--CTCGAATGTGTGTGCGTCCATCCCGCCGCAAGCTCGGTCAGTTCTTACCCGGAGGCCACGGACCCAGTGCCGTCCCCGGGCCTGGCCAGCTGTTAGCGGGCGGTGTCCTCGGACCGGCCAAACCTCGGATTACCGGTCGGCGACGCTATTGCTTTGGGTACTCTCAGGACCCGTCTTGAAACACGGACCAAGGAGTCTAACATGTGCGCGAGTCATTGGGATGCATAAACCTAAAGGCGAAATGAAAGTGAAAGTCGGCCTTCGCGCCGATCGAGGGAGGATGGGCCGCGTAACAATGCGGCCCCGCACTCCCGGGGCGTCTCGTTCTCACTGCGAGAAGAGGCG

>HT51 NSTCHUC38

GGAATTCTGTATATAATTTTTGCTATCTGATCAGGTATTGTTGGTTCTTCCTTCAGAATACTTATTCGAATGGAACTTAATAGCCCTGGAACTTGAATTAGTAATGATCAGATCTACAACTCTATTGTGACTAGTCATGCATTTCTAATAATTTTTTTTATAGTAATACCTTTTATAATCGGAGGCTTTGGAAATTTTTTAATCCCGTTAATACTAGGTTCCCCTGATATAACTTTCCCTCGAATAAACAATATTAACTTCTGACTACTACCTCCTTCACTTATACTGCTAATAACAAATAATCTATTTTTTCCGAACTCAGGAACTGGATGAACTGTATACCCTCCTCTTTCTCTTTACATATATCATCCATCTCCGTCAGTGGACTTTACTATTTTTTCAATTCATATAACAGGAATTTCATCAATTCTTGGGTCACTTAACTTTATTGTAACAATTTTTATAATAAAGAACTTTTCTTTAAAGTACGATCAAATTAATCTTTTTTCATGATCTATCTCAATTACTGTAATTTTACTAATTCTTTCTCTTCCAGTTCTAGCAGGAGCAATTACTATGCTGTTATTTGATCGAAATTTTAATACTTCCTTCTTTGATCCAATAGGAGGGGGAGACCCGATTCTTTATCAACATTTATTTATCTGCCCAATGAATTT-----TTTTAATGGCTGCAGTATAACTGACTGTACAAAGGTAGCAT---CAATTGGTTTT---ATGAAATCTGGAATGAAAGGATTAATGAAATATGTACTGTCTCAATTGTACAATTATGAAATTAAAATTTTAATAAAAATGTTAAAATTCACTTATGGGACGATAAGACCCTATAGAATTTTATATTGAAATTACTCAGTAGTTAATTCAGAAATAGTTTCAATATTTGATTGGGAGGATTAT---ACT---CCAACTT--TAATT---GTTAACTTTAATTTAAGA-GTAGATAATGATCTTCAATTTGAAATTGCTAGAATAAATTACCTTAGGGATAACAGCGTAATACTTTTTTATAGGCCATATAGAAAAAAGTGGTTGCGACCTCGATGTTGAATTAGGATAAATTTTAAATGCAGGAGTTTAATAATTAAGTCTGTCGTCGACGGCGTAGGCTACCGTTGGTGCGCGATGCTCCGGATGGACCCCC-GCGGTTCCAT---CGAGGGCACGCCACCCTCGGATCGAACGCTCCTGCGTCGT---CGTGCACTTCTCCCCTAGTAGAACGTCGCGACCCGTTGTGTGTCGGTCTACGGCCCGAGCGGGAGACTGTCGCGTCGCTTCGGCGCACGCGGCAGACCCTCGGTCGCCCGGCCGGCTGCACGACGGTACACTCACGGTATCGGGCCGCAGCCAATCCATT--CTCGAATGTGTGTGCGTCCATCCCGCCGCAAGCTCGGTCAGTTCTTACCCGGAGGCCACGGACCCAGTGCCGTCCCCGGGCCTGGCCAGCTGTTAGCGGGCGGTGTCCTCGGACCGGCCAAACCTCGGATTACCGGTCGGCGACGCTATTGCTTTGGGTACTCTCAGGACCCGTCTTGAAACACGGACCAAGGAGTCTAACATGTGCGCGAGTCATTGGGATGCATAAACCTAAAGGCGAAATGAAAGTGAAAGTCGGCCTTCGCGCCGATCGAGGGAGGATGGGCCGCGTAACAATGCGGCCCCGCACTCCCGGGGCGTCTCGTTCTCACTGCGAGAAGAGGCG

>HT52 NSTCHUC39

GGAATTCTGTATATAATTTTTGCTATCTGATCAGGTATTGTTGGTTCTTCCTTCAGAATACTTATTCGAATGGAACTTAATAGCCCTGGAACTTGAATTAGTAATGATCAGATCTACAACTCTATTGTGACTAGTCATGCATTTCTAATAATTTTTTTTATAGTAATACCTTTTATAATCGGAGGCTTTGGAAATTTTTTAATCCCGTTAATACTAGGTTCCCCTGATATAACTTTCCCTCAAATAAACAATATTAACTTCTGACTACTACCTCCTTCACTTATACTGCTAATAACAAATAATCTATTTTTTCCGAACTCAGGAACTGGATGAACTGTATACCCTCCTCTTTCTCTTTACATATATCATCCATCTCCGTCAGTGGACTTTACTATTTTTTCAATTCATATAACAGGAATTTCATCAATTCTTGGGTCACTTAACTTTATTGTAACAATTTTTATAATAAAGAACTTTTCTTTAAAGTACGATCAAATTAATCTTTTTTCATGATCTATCTCAATTACTGTAATTTTACTAATTCTTTCTCTTCCAGTTCTAGCAGGAGCAATTACTATGCTGTTATTTGATCGAAATTTTAATACTTCCTTCTTTGATCCAATAGGAGGGGGAGACCCGATTCTTTATCAACATTTATTTATCTGCCCAATGAATTT-----TTTTAATGGCTGCAGTATAACTGACTGTACAAAGGTAGCAT---CAATTGGTTTT---ATGAAATCTGGAATGAAAGGATTAATGAAATATGTACTGTCTCAATTGTACAATTATGAAATTAAAATTTTAATAAAAATGTTAAAATTCACTTATGGGACGATAAGACCCTATAGAATTTTATATTGAAATTACTCAGTAGTTAATTCAGAAATAGTTTCAATATTTGATTGGGAGGATTAT---ACT---CCAACTT--TAATT---GTTAACTTTAATTTAAGA-GTAGATAATGATCTTCAATTTGAAATTGCTAGAATAAATTACCTTAGGGATAACAGCGTAATACTTTTTTATAGGCCATATAGAAAAAAGTGGTTGCGACCTCGATGTTGAATTAGGATAAATTTTAAATGCAGGAGTTTAATAATTAAGTCTGTCGTCGACGGCGTAGGCTACCGTTGGTGCGCGATGCTCCGGATGGACCCCC-GCGGTTCCAT---CGAGGGCACGCCACCCTCGGATCGAACGCTCCTGCGTCGT---CGTGCACTTCTCCCCTAGTAGAACGTCGCGACCCGTTGTGTGTCGGTCTACGGCCCGAGCGGGAGACTGTCGCGTCGCTTCGGCGCACGCGGCAGACCCTCGGTCGCCCGGCCGGCTGCACGACGGTACACTCACGGTATCGGGCCGCAGCCAATCCATT--CTCGAATGTGTGTGCGTCCATCCCGCCGCAAGCTCGGTCAGTTCTTACCCGGAGGCCACGGACCCAGTGCCGTCCCCGGGCCTGGCCAGCTGTTAGCGGGCGGTGTCCTCGGACCGGCCAAACCTCGGATTACCGGTCGGCGACGCTATTGCTTTGGGTACTCTCAGGACCCGTCTTGAAACACGGACCAAGGAGTCTAACATGTGCGCGAGTCATTGGGATGCATAAACCTAAAGGCGAAATGAAAGTGAAAGTCGGCCTTCGCGCCGATCGAGGGAGGATGGGCCGCGTAACAATGCGGCCCCGCACTCCCGGGGCGTCTCGTTCTCACTGCGAGAAGAGGCG

>HT53 NSTCHUN6

GGAATTCTGTATATAATTTTTGCTATCTGATCAGGTATTGTTGGTTCTTCCTTCAGAATACTTATTCGAATGGAACTTAATAGCCCTGGAACTTGAATTAGTAATGATCAGATCTACAACTCTATTGTGACTAGTCATGCATTTCTAATAATTTTTTTTATAGTAATACCTTTTATAATCGGAGGCTTTGGAAATTTTTTAATCCCGTTAATACTAGGTTCCCCTGATATAACTTTCCCTCGAATAAACAATATTAGCTTCTGACTACTACCTCCTTCACTTATACTGCTAATAACAAATAATCTATTTTTTCCGAACTCAGGAACTGGATGAACTGTATACCCTCCTCTTTCTCTTTACATATATCATCCATCTCCGTCAGTGGACTTTACTATTTTTTCAATTCATATAACAGGAATTTCATCAATTCTTGGGTCACTTAACTTTATTGTAACAATTTTTATAATAAAGAACTTTTCTTTAAAGTACGATCAAATTAATCTTTTTTCATGATCTATCTCAATTACTGTAATTTTACTAATTCTTTCTCTTCCAGTTCTAGCAGGAGCAATTACTATGCTGTTATTTGATCGAAATTTTAATACTTCCTTCTTTGATCCAATAGGAGGGGGAGACCCGATTCTTTATCAACATTTATTTATCTGCCCAATGAATTT-----TTTTAATGGCTGCAGTATAACTGACTGTACAAAGGTAGCAT---CAATTGGTTTT---ATGAAATCTGGAATGAAAGGATTAATGAAATATGTACTGTCTCAATTGTACAATTATGAAATTAAAATTTTAATAAAAATGTTAAAATTCACTTATGGGACGATAAGACCCTATAGAATTTTATATTGAAATTACTCAGTAGTTAATTCAGAAATAGTTTCAATATTTGATTGGGAGGATTAT---ACT---CCAACTT--TAATT---GTTAACTTTAATTTAAGA-GTAGATAATGATCTTCAATTTGAAATTGCTAGAATAAATTACCTTAGGGATAACAGCGTAATACTTTTTTATAGGCCATATAGAAAAAAGTGGTTGCGACCTCGATGTTGAATTAGGATAAATTTTAAATGCAGGAGTTTAATAATTAAGTCTGTCGTCGACGGCGTAGGCTACCGTTGGTGCGCGATGCTCCGGATGGACCCCC-GCGGTTCCAT---CGAGGGCACGCCACCCTCGGATCGAACGCTCCTGCGTCGT---CGTGCACTTCTCCCCTAGTAGAACGTCGCGACCCGTTGTGTGTCGGTCTACGGCCCGAGCGGGAGACTGTCGCGTCGCTTCGGCGCACGCGGCAGACCCTCGGTCGCCCGGCCGGCTGCACGACGGTACACTCACGGTATCGGGCCGCAGCCAATCCATT--CTCGAATGTGTGTGCGTCCATCCCGCCGCAAGCTCGGTCAGTTCTTACCCGGAGGCCACGGACCCAGTGCCGTCCCCGGGCCTGGCCAGCTGTTAGCGGGCGGTGTCCTCGGACCGGCCAAACCTCGGATTACCGGTCGGCGACGCTATTGCTTTGGGTACTCTCAGGACCCGTCTTGAAACACGGACCAAGGAGTCTAACATGTGCGCGAGTCATTGGGATGCATAAACCTAAAGGCGAAATGAAAGTGAAAGTCGGCCTTCGCGCCGATCGAGGGAGGATGGGCCGCGTAACAATGCGGCCCCGCACTCCCGGGGCGTCTCGTTCTCACTGCGAGAAGAGGCG

>HT54 NSTCHUN7

GGAATTCTGTATATAATTTTTGCTATCTGATCAGGTATTGTTGGTTCTTCCTTCAGAATACTTATTCGAATGGAACTTAATAGCCCTGGAACTTGAATTAGTAATGATCAGATCTACAACTCTATTGTGACTAGTCATGCATTTCTAATAATTTTTTTTATAGTAATACCTTTTATAATCGGAGGCTTTGGAAATTTTTTAATCCCGTTAATACTAGGTTCCCCTGATATAGCTTTCCCTCGAATAAACAATATTAGCTTCTGACTACTACCTCCTTCACTTATACTGCTAATAACAAATAATCTATTTTTTCCGAACTCAGGAACTGGATGAACTGTATACCCTCCTCTTTCTCTTTACATATATCATCCATCTCCGTCAGTGGACTTTACTATTTTTTCAATTCATATAACAGGAATTTCATCAATTCTTGGGTCACTTAACTTTATTGTAACAATTTTTATAATAAAGAACTTTTCTTTAAAGTACGATCAAATTAATCTTTTTTCATGATCTATCTCAATTACTGTAATTTTACTAATTCTTTCTCTTCCAGTTCTAGCAGGAGCAATTACTATGCTGTTATTTGATCGAAATTTTAATACTTCCTTCTTTGATCCAATAGGAGGGGGAGACCCGATTCTTTATCAACATTTATTTATCTGCCCAATGAATTT-----TTTTAATGGCTGCAGTATAACTGACTGTACAAAGGTAGCAT---CAATTGGTTTT---ATGAAATCTGGAATGAAAGGATTAATGAAATATGTACTGTCTCAATTGTACAATTATGAAATTAAAATTTTAATAAAAATGTTAAAATTCACTTATGGGACGATAAGACCCTATAGAATTTTATATTGAAATTACTCAGTAGTTAATTCAGAAATAGTTTCAATATTTGATTGGGAGGATTAT---ACT---CCAACTT--TAATT---GTTAACTTTAATTTAAGA-GTAGATAATGATCTTCAATTTGAAATTGCTAGAATAAATTACCTTAGGGATAACAGCGTAATACTTTTTTATAGGCCATATAGAAAAAAGTGGTTGCGACCTCGATGTTGAATTAGGATAAATTTTAAATGCAGGAGTTTAATAATTAAGTCTGTCGTCGACGGCGTAGGCTACCGTTGGTGCGCGATGCTCCGGATGGACCCCC-GCGGTTCCAT---CGAGGGCACGCCACCCTCGGATCGAACGCTCCTGCGTCGT---CGTGCACTTCTCCCCTAGTAGAACGTCGCGACCCGTTGTGTGTCGGTCTACGGCCCGAGCGGGAGGCTGTCGCGTCGCTTCGGCGCACGCGGCAGACCCTCGGTCGCCCGGCCGGCTGCACGACGGTACACTCACGGTATCGGGCCGCAGCCAATCCATT--CTCGAATGTGTGTGCGTCCATCCCGCCGCAAGCTCGGTCAGTTCTTACCCGGAGGCCACGGACCCAGTGCCGTCCCCGGGCCTGGCCAGCTGTTAGCGGGCGGTGTCCTCGGACCGGCCAAACCTCGGATTACCGGTCGGCGACGCTATTGCTTTGGGTACTCTCAGGACCCGTCTTGAAACACGGACCAAGGAGTCTAACATGTGCGCGAGTCATTGGGATGCATAAACCTAAAGGCGAAATGAAAGTGAAAGTCGGCCTTCGCGCCGATCGAGGGAGGATGGGCCGCGTAACAATGCGGCCCCGCACTCCCGGGGCGTCTCGTTCTCACTGCGAGAAGAGGCG

>HT55 NSTCHUN8

GGAATTCTGTATATAATTTTTGCTATCTGATCAGGTATTGTTGGTTCTTCCTTCAGAATACTTATTCGAATGGAACTTAATAGCCCTGGAACTTGAATTAGTAATGATCAGATCTACAACTCTATTGTGACTAGTCATGCATTTCTAATAATTTTTTTTATAGTAATACCTTTTATAATCGGAGGCTTTGGAAATTTTTTAATCCCGTTAATACTAGGTTCCCCTGATATAACTTTCCCTCAAATAAACAATATTAACTTCTGACTACTACCTCCTTCACTTATACTGCTAATAACAAATAATCTATTTTTTCCGAACTCAGGAACTGGATGAACTGTATACCCTCCTCTTTCTCTTTACATATATCATCCATCTCCGTCAGTGGACTTTACTATTTTTTCAATTCATATAACAGGAATTTCATCAATTCTTGGGTCACTTAACTTTATTGTAACAATTTTTATAATAAAGAACTTTTCTTTAAAGTACGATCAAATTAATCTTTTTTCATGATCTATCTCAATTACTGTAATTTTACTAATTCTTTCTCTTCCAGTTCTAGCAGGAGCAATTACTATGCTGTTATTTGATCGAAATTTTAATACTTCCTTCTTTGATCCAATAGGAGGGGGAGACCCGATTCTTTATCAACATTTATTTATCTGCCCAATGAATTT-----TTTTAATGGCTGCAGTATAACTGACTGTACAAAGGTAGCAT---CAATTGGTTTT---ATGAAATCTGGAATGAAAGGATTAATGAAATATGTACTGTCTCAATTGTACAATTATGAAATTAAAATTTTAATAAAAATGTTAAAATTCACTTATGGGACGATAAGACCCTATAGAATTTTATATTGAAATTACTCAGTAGTTAATTCAGAAATAGTTTCAATATTTGATTGGGAGGATTAT---ACT---CCAACTT--TAATT---GTTAACTTTAATTTAAGA-GTAGATAATGATCTTCAATTTGAAATTGCTAGAATAAATTACCTTAGGGATAACAGCGTAATACTTTTTTATAGGCCATATAGAAAAAAGTGGTTGCGACCTCGATGTTGAATTAGGATAAATTTTAAATGCAGGAGTTTAATAATTAAGTCTGTCGTCGACGGCGTAGGCTACCGTTGGTGCGCGATGCTCCGGATGGACCCCC-GCGGTTCCAT---CGAGGGCACGCCACCCTCGGATCGAACGCTCCTGCGTCGT---CGTGCACTTCTCCCCTAGTAGAACGTCGCGACCCGTTGTGTGTCGGTCTACGGCCCGAGCGGGAGACTGTCGCGTCGCTTCGGCGCACGCGGCAGACCCTCGGTCGCCCGGCCGGCTGCACGACGGTACACTCACGGTATCGGGCCGCAGCCAATCCATT--CTCGAATGTGTGTGCGTCCATCCCGCCGCAAGCTCGGTCAGTTCTTACCCGGAGGCCACGGACCCAGTGCCGTCCCCGGGCCTGGCCAGCTGTTAGCGGGCGGTGTCCTCGGACCGGCCAAACCTCGGATTACCGGTCGGCGACGCTATTGCTTTGGGTACTCTCAGGACCCGTCTTGAAACACGGACCAAGGAGTCTAACATGTGCGCGAGTCATTGGGATGCATAAACCTAAAGGCGAAATGAAAGTGAAAGTCGGCCTTCGCGCCGATCGAGGGAGGATGGGCCGCGTAACAATGCGGCCCCGCACTCCCGGGGCGTCTCGTTCTCACTGCGAGAAGAGGCG

>HT56 NSTCHUN9

GGAATTCTGTATATAATTTTTGCTATCTGATCAGGTATTGTTGGTTCTTCCTTCAGAATACTTATTCGAATGGAACTTAATAGCCCTGGAACTTGAATTAGTAATGATCAGATCTACAACTCTATTGTGACTAGTCATGCATTTCTAATAATTTTTTTTATAGTAATACCTTTTATAATCGGAGGCTTTGGAAATTTTTTAATCCCGTTAATACTAGGTTCCCCTGATATAACTTTCCCTCAAATAAACAATATTAACTTCTGACTACTACCTCCTTCACTTATACTGCTAATAACAAATAATCTATTTTTTCCGAATTCAGGAACTGGATGAACTGTATACCCTCCTCTTTCTCTTTACATATATCATCCATCTCCGTCAGTGGACTTTACTATTTTTTCAATTCATATAACAGGAATTTCATCAATTCTTGGGTCACTTAACTTTATTGTAACAATTTTTATAATAAAGAACTTTTCTTTAAAGTACGATCAAATTAATCTTTTTTCATGATCTATCTCAATTACTGTAATTTTACTAATTCTTTCTCTTCCAGTTCTAGCAGGAGCAATTACTATGCTGTTATTTGATCGAAATTTTAATACTTCCTTCTTTGATCCAATAGGAGGGGGAGACCCGATTCTTTATCAACATTTATTTATCTGCCCAATGAATTT-----TTTTAATGGCTGCAGTATAACTGACTGTACAAAGGTAGCAT---CAATTGGTTTT---ATGAAATCTGGAATGAAAGGATTAATGAAATATGTACTGTCTCAATTGTACAATTATGAAATTAAAATTTTAATAAAAATGTTAAAATTCACTTATGGGACGATAAGACCCTATAGAATTTTATATTGAAATTACTCAGTAGTTAATTCAGAAATAGTTTCAATATTTGATTGGGAGGATTAT---ACT---CCAACTT--TAATT---GTTAACTTTAATTTAAGA-GTAGATAATGATCTTCAATTTGAAATTGCTAGAATAAATTACCTTAGGGATAACAGCGTAATACTTTTTTATAGGCCATATAGAAAAAAGTGGTTGCGACCTCGATGTTGAATTAGGATAAATTTTAAATGCAGGAGTTTAATAATTAAGTCTGTCGTCGACGGCGTAGGCTACCGTTGGTGCGCGATGCTCCGGATGGACCCCC-GCGGTTCCAT---CGAGGGCACGCCACCCTCGGATCGAACGCTCCTGCGTCGT---CGTGCACTTCTCCCCTAGTAGAACGTCGCGACCCGTTGTGTGTCGGTCTACGGCCCGAGCGGGAGGCTGTCGCGTCGCTTCGGCGCACGCGGCAGACCCTCGGTCGCCCGGCCGGCTGCACGACGGTACACTCACGGTATCGGGCCGCAGCCAATCCATT--CTCGAATGTGTGTGCGTCCATCCCGCCGCAAGCTCGGTCAGTTCTTACCCGGAGGCCACGGACCCAGTGCCGTCCCCGGGCCTGGCCAGCTGTTAGCGGGCGGTGTCCTCGGACCGGCCAAACCTCGGATTACCGGTCGGCGACGCTATTGCTTTGGGTACTCTCAGGACCCGTCTTGAAACACGGACCAAGGAGTCTAACATGTGCGCGAGTCATTGGGATGCATAAACCTAAAGGCGAAATGAAAGTGAAAGTCGGCCTTCGCGCCGATCGAGGGAGGATGGGCCGCGTAACAATGCGGCCCCGCACTCCCGGGGCGTCTCGTTCTCACTGCGAGAAGAGGCG

>HT57 NSTCHUN10

GGAATTCTGTATATAATTTTTGCTATCTGATCAGGTATTGTTGGTTCTTCCTTCAGAATACTTATTCGAATGGAACTTAATAGCCCTGGAACTTGAATTAGTAATGATCAGATCTACAACTCTATTGTGACTAGTCATGCATTTCTAATAATTTTTTTTATAGTAATACCTTTTATAATCGGAGGCTTTGGAAATTTTTTAATCCCGTTAATACTAGGTTCCCCTGATATAGCTTTCCCTCGAATAAACAATATTAGCTTCTGACTACTACCTCCTTCACTTATACTGCTAATAACAAATAATCTATTTTTTCCGAACTCAGGAACTGGATGAACTGTATACCCTCCTCTTTCTCTTTACATATATCATCCATCTCCGTCAGTGGACTTTACTATTTTTTCAATTCATATAACAGGAATTTCATCAATTCTTGGGTCACTTAACTTTATTGTAACAATTTTTATAATAAAGAACTTTTCTTTAAAGTACGATCAAATTAATCTTTTTTCATGATCTATCTCAATTACTGTAATTTTACTAATTCTTTCTCTTCCAGTTCTAGCAGGAGCAATTACTATGCTGTTATTTGATCGAAATTTTAATACTTCCTTCTTTGATCCAATAGGAGGGGGAGACCCGATTCTTTATCAACATTTATTTATCTGCCCAATGAATTT-----TTTTAATGGCTGCAGTATAACTGACTGTACAAAGGTAGCAT---CAATTGGTTTT---ATGAAATCTGGAATGAAAGGATTAATGAAATATGTACTGTCTCAATTGTACAATTATGAAATTAAAATTTTAATAAAAATGTTAAAATTCACTTATGGGACGATAAGACCCTATAGAATTTTATATTGAAATTACTCAGTAGTTAATTCAGAAATAGTTTCAATATTTGATTGGGAGGATTAT---ACT---CCAACTT--TAATT---GTTAACTTTAATTTAAGA-GTAGATAATGATCTTCAATTTGAAATTGCTAGAATAAATTACCTTAGGGATAACAGCGTAATACTTTTTTATAGGCCATATAGAAAAAAGTGGTTGCGACCTCGATGTTGAATTAGGATAAATTTTAAATGCAGGAGTTTAATAATTAAGTCTGTCGTCGACGGCGTAGGCTACCGTTGGTGCGCGATGCTCCGGATGGACCCCC-GCGGTTCCAT---CGAGGGCACGCCACCCTCGGATCGAACGCTCCTGCGTCGT---CGTGCACTTCTCCCCTAGTAGAACGTCGCGACCCGTTGTGTGTCGGTCTACGGCCCGAGCGGGAGACTGTCGCGTCGCTTCGGCGCACGCGGCAGACCCTCGGTCGCCCGGCCGGCTGCACGACGGTACACTCACGGTATCGGGCCGCAGCCAATCCATT--CTCGAATGTGTGTGCGTCCATCCCGCCGCAAGCTCGGTCAGTTCTTACCCGGAGGCCACGGACCCAGTGCCGTCCCCGGGCCTGGCCAGCTGTTAGCGGGCGGTGTCCTCGGACCGGCCAAACCTCGGATTACCGGTCGGCGACGCTATTGCTTTGGGTACTCTCAGGACCCGTCTTGAAACACGGACCAAGGAGTCTAACATGTGCGCGAGTCATTGGGATGCATAAACCTAAAGGCGAAATGAAAGTGAAAGTCGGCCTTCGCGCCGATCGAGGGAGGATGGGCCGCGTAACAATGCGGCCCCGCACTCCCGGGGCGTCTCGTTCTCACTGCGAGAAGAGGCG

>HT58 NSTCHUN11

GGAATTCTGTATATAATTTTTGCTATCTGATCAGGTATTGTTGGTTCTTCCTTCAGAATACTTATTCGAATGGAACTTAATAGCCCTGGAACTTGAATTAGTAATGATCAAATCTACAACTCTATTGTGACTAGTCATGCATTTCTAATAATTTTTTTTATAGAAATACCTTTTATAATCGGAGGCTTTGGAAATTTTTTAATCCCGTTAATACTAGGTTCCCCTGATATAACTTTCCCTCAAATAAACAATATTAACTTCTGACTACTACCTCCTTCACTTATACTGCTAATAACAAATAATCTATTTTTTCCGAACTCAGGAACTGGATGAACTGTATACCCTCCTCTTTCTCTTTACATATATCATCCATCTCCGTCAGTGGACTTTACTATTTTTTCAATTCATATAACAGGAATTTCATCAATTCTTGGGTCACTTAACTTTATTGTAACAATTTTTATAATAAAGAACTTTTCTTTAAAGTACGATCAAATTAATCTTTTTTCATGATCTATCTCAATTACTGTAATTTTACTAATTCTTTCTCTTCCAGTTCTAGCAGGAGCAATTACTATGCTGTTATTTGATCGAAATTTTAATACTTCCTTCTTTGATCCAATAGGAGGGGGAGACCCGATTCTTTATCAACATTTATTT-------------------------------------------------------------------------------------------------------------------------------------------------------------------------------------------------------------------------------------------------------------------------------------------------------------------------------------------------------------------------------------------------------------------------------------------------------------------------------CGTCGACGGCGTAGGCTACCGTTGGTGCGCGATGCTCCGGATGGACCCCC-GCGGTTCCAT---CGAGGGCACGCCACCCTCGGATCGAACGCTCCTGCGTCGT---CGTGCACTTCTCCCCTAGTAGAACGTCGCGACCCGTTGTGTGTCGGTCTACGGCCCGAGCGGGAGACTGTCGCGTCGCTTCGGCGCACGCGGCAGACCCTCGGTCGCCCGGCCGGCTGCACGACGGTACACTCACGGTATCGGGCCGCAGCCAATCCATT--CTCGAATGTGTGTGCGTCCATCCCGCCGCAAGCTCGGTCAGTTCTTACCCGGAGGCCACGGACCCAGTGCCGTCCCCGGGCCTGGCCAGCTGTTAGCGGGCGGTGTCCTCGGACCGGCCAAACCTCGGATTACCGGTCGGCGACGCTATTGCTTTGGGTACTCTCAGGACCCGTCTTGAAACACGGACCAAGGAGTCTAACATGTGCGCGAGTCATTGGGATGCATAAACCTAAAGGCGAAATGAAAGTGAAAGTCGGCCTTCGCGCCGATCGAGGGAGGATGGGCCGCGTAACAATGCGGCCCCGCACTCCCGGGGCGTCTCGTTCTCACTGCGAGAAGAGGCG

>HT59 NSTCHUN12

GGAATTCTGTATATAATTTTTGCTATCTGATCAGGTATTGTTGGTTCTTCCTTCAGAATACTTATTCGAATGGAACTTAATAGCCCTGGAACTTGAATTAGTAATGATCAAATCTACAACTCTATTGTGACTAGTCATGCATTTCTAATAATTTTTTTTATAGTAATACCTTTTATAATCGGAGGCTTTGGAAATTTTTTAATCCCGTTAATACTAGGTTCCCCTGATATAACTTTCCCTCAAATAAACAATATTAACTTCTGACTACTACCTCCTTCACTTATACTGCTAATAACAAATAATCTATTTTTTCCGAAGTCAGGAACTGGATGAACTGTATACCCTCCTCTTTCTCTTTACATATATCATCCATCTCCGTCAGTGGACTTTACTATTTTTTCAATTCATATAACAGGAATTTCATCAATTCTTGGGTCACTTAACTTTATTGTAACAATTTTTATAATAAAGAACTTTTCTTTAAAGTACGATCAAATTAATCTTTTTTCATGATCTATCTCAGTTACTGTAATTTTACTAATTCTTTCTCTTCCAGTTCTAGCAGGAGCAATTACTATGCTGTTATTTGATCGAAATTTTAATACTTCCTTCTTTGATCCAATAGGAGGGGGAGACCCGATTCTTTATCAACATTTATTTATCTGCCCAATGAATTT-----TTTTAATGGCTGCAGTATAACTGACTGTACAAAGGTAGCAT---CAATTGGTTTT---ATGAAATCTGGAATGAAAGGATTAATGAAATATGTACTGTCTCAATTGTACAATTATGAAATTAAAATTTTAATAAAAATGTTAAAATTCACTTATGGGACGATAAGACCCTATAGAATTTTATATTGAAATTACTCAGTAGTTAATTCAGAAATAGTTTCAATATTTGATTGGGAGGATTAT---ACT---CCAACTT--TAATT---GTTAACTTTAATTTAAGA-GTAGATAATGATCTTCAATTTGAAATTGCTAGAATAAATTACCTTAGGGATAACAGCGTAATACTTTTTTATAGGCCATATAGAAAAAAGTGGTTGCGACCTCGATGTTGAATTAGGATAAATTTTAAATGCAGGAGTTTAATAATTAAGTCTGTCGTCGACGGCGTAGGCTACCGTTGGTGCGCGATGCTCCGGATGGACCCCC-GCGGTTCCAT---CGAGGGCACGCCACCCTCGGATCGAACGCTCCTGCGTCGT---CGTGCACTTCTCCCCTAGTAGAACGTCGCGACCCGTTGTGTGTCGGTCTACGGCCCGAGCGGGAGACTGTCGCGTCGCTTCGGCGCACGCGGCAGACCCTCGGTCGCCCGGCCGGCTGCACGACGGTACACTCACGGTATCGGGCCGCAGCCAATCCATT--CTCGAATGTGTGTGCGTCCATCCCGCCGCAAGCTCGGTCAGTTCTTACCCGGAGGCCACGGACCCAGTGCCGTCCCCGGGCCTGGCCAGCTGTTAGCGGGCGGTGTCCTCGGACCGGCCAAACCTCGGATTACCGGTCGGCGACGCTATTGCTTTGGGTACTCTCAGGACCCGTCTTGAAACACGGACCAAGGAGTCTAACATGTGCGCGAGTCATTGGGATGCATAAACCTAAAGGCGAAATGAAAGTGAAAGTCGGCCTTCGCGCCGATCGAGGGAGGATGGGCCGCGTAACAATGCGGCCCCGCACTCCCGGGGCGTCTCGTTCTCACTGCGAGAAGAGGCG

>HT60 NSTCHUN13

GGAATTCTGTATATAATTTTTGCTATCTGATCAGGTATTGTTGGTTCTTCCTTCAGAATACTTATTCGAATGGAGCTTAATAGTCCTGGAACTTGAATTAATAATGATCAAATTTACAATTCTATTGTAACTAGTCATGCATTTCTAATAATTTTCTTTATAGTTATACCCTTTATAATTGGAGGGTTCGGGAACTTTCTAATTCCACTAATAATGGGTTCCCCTGATATAGCCTTTCCTCGAATAAATAACGTTAGATTTTGATTACTTCCACCCTCAATTATACTACTTATAACAAATAATCTATTTTATCCTAATTCAGGAACTGGATGAACTGTATATCCTCCACTTTCTCTTTATATATATCATTCATCTCCATCTGTAGATTTCACTATTTTTTCAATTCATATAACAGGAATTTCATCAATTCTAGGATCTCTTAATTTTATTGTTACAATTTTTATAATAAAAAATTTCTCTTTGAAGTATGATCAAATTAATCTATTTTCATGATCTATCTCAATTACTGTAATCTTACTAATTCTTTCCCTTCCGGTTCTAGCGGGAGCAATTACTATACTTTTATTTGACCGAAACTTCAACACCTCATTTTTTGATCCAATAGGAGGAGGAGATCCTATTCTATACCAACATCTGTTCATCTGCCCAATGAAGTT-----CTTTAATGGCTGCAGTATAACTGACTGTACAAAGGTAGCAT---CAATTGGTTTT---ATGAAATCTGGAATGAAAGGATTAATGAAATATGTACTGTCTCAATTGTATAATTATGAAATTAAAATTTTAATAAAAATGTTAAAATTTACTTATGGGACGATAAGACCCTATAGAATTTTATATTGAAATTACTCAGTAGTTAGTACAGAAATAATTTTAATATTTGGTTGGGAGGACTAT---ATT---CTAACTT--TAATT---ATTAACTTTAATTTAAGA-GTAAATAAGGATCTTCAATTTGAAATTATCAGAGTAAATTACCTTAGGGATAACAGCGTAATACTTTTTTATAGGCCATATAGAAAAAAGTGATTGCGACCTCGATGTTGAATTAGGATAAATTTTAAATGCAGGAGTTTAATAATTAAGTCTGTCGTCGACGGCGTAGGCTACCGTTGGTGCGCGATGCTCCGGATGGACCCCC-GCGGTTCCAT---CGAGGGCACGCCACCCTCGGATCGAACGCTCCTGCGTCGT---CGTGCACTTCTCCCCTAGTAGAACGTCGCGACCCGTTGTGTGTCGGTCTACGGCCCGAGCGGGAGACTGTCGCGTCGCTTCGGCGCACGCGGCAGACCCTCGGTCGCCCGGCCGGCTGCACGACGGTACACTCACGGTATCGGGCCGCAGCCAATCCATT--CTCGAATGTGTGTGCGTCCATCCCGCCGCAAGCTCGGTCAGTTCTTACCCGGAGGCCACGGACCCAGTGCCGTCCCCGGGCCTGGCCAGCTGTTAGCGGGCGGTGTCCTCGGACCGGCCAAACCTCGGATTACCGGTCGGCGACGCTATTGCTTTGGGTACTCTCAGGACCCGTCTTGAAACACGGACCAAGGAGTCTAACATGTGCGCGAGTCATTGGGATGCATAAACCTAAAGGCGAAATGAAAGTGAAAGTCGGCCTTCGCGCCGATCGAGGGAGGATGGGCCGCGTAACAATGCGGCCCCGCACTCCCGGGGCGTCTCGTTCTCACTGCGAGAAGAGGCG

>HT61 NSTCHUN14

GGAATTCTATATATAATTTTTGCTATCTGATCAGGTATTGTTGGTTCTTCCTTCAGAATACTTATTCGAATGGAACTTAATAGCCCTGGAACTTGAATTAGTAATGATCAGATCTACAACTCTATTGTGACTAGGCATGCATTTCTAATAATTTTTTTTATAGTAATACCTTTTATAATCGGGGGTTTTGGAAATTTTTTAATTCCGCTAATACTAGGTTCTCCTGATATAGCTTTCCCTCGAATGAACAATATTAGCTTCTGACTACTACCTCCTTCACTTATACTGCTAATAACAAATAATTTATTCTTTCCGAATTCAGGAACTGGATGAACTGTGTACCCCCCTCTTTCTCTCTACATATATCATTCATCTCCGTCAGTGGACTTTACTATTTTTTCAATTCATATAACAGGAATTTCATCAATTCTTGGGTCACTTAACTTTATTGTAACAATTTTTATAATAAAGAACTTTTCTTTGAAGTACGATCAAATTAATCTTTTTTCATGATCTATCTCAATCACTGTAATTTTACTAATTCTTTCTCTTCCAGTTCTAGCAGGAGCAATTACTATGCTGTTATTTGATCGAAATTTTAATACTTCCTTCTTTGATCCAATAGGAGGGGGAGACCCGATCCTTTATCAACATTTGTTTATCTGCCCAATGAATTC-----TTTTAATGGCTGCAGTATAACTGACTGTACAAAGGTAGCAT---CAATTGGTTTT---ATGAAATCTGGAATGAAAGGATTAATGAAATATGTACTGTCTCAATTGTACAATTATGAAATTAAAATTTTAATAAAAATGTTAAAATTTACTTATGGGACGATAAGACCCTATAGAATTTTATATTGAAATTACTCAGTAGTTAATTCAGAAATAGTTTCAATATTTGATTGGGAGGATTAT---ACT---CCAACTT--TAATT---GTTAACTTTAATTTAAGG-GTAGATAATGATCTTCAATTTGAAATTGCTAGAATAAATTACCTTAGGGATAACAGCGTAATACTTTTTTATAGGCCATATAGAAAAAAGTGGTTGCGACCTCGATGTTGAATTAGGATAAATTTTAAATGCAGGAGTTTAATAATTAAGTCTGTCGTCGACGGCGTAGGCTACCGTTGGTGCGCGATGCTCCGGATGGACCCCC-GCGGTTCCAT---CGAGGGCACGCCACCCTCGGATCGAACGCTCCTGCGTCGT---CGTGCACTTCTCCCCTAGTAGAACGTCGCGACCCGTTGTGTGTCGGTCTACGGCCCGAGCGGGAGACTGTCGCGTCGCTTCGGCGCACGCGGCAGACCCTCGGTCGCCCGGCCGGCTGCACGACGGTACACTCACGGTATCGGGCCGCAGCCAATCCATT--CTCGAATGTGTGTGCGTCCATCCCGCCGCAAGCTCGGTCAGTTCTTACCCGGAGGCCACGGACCCAGTGCCGTCCCCGGGCCTGGCCAGCTGTTAGCGGGCGGTGTCCTCGGACCGGCCAAACCTCGGATTACCGGTCGGCGACGCTATTGCTTTGGGTACTCTCAGGACCCGTCTTGAAACACGGACCAAGGAGTCTAACATGTGCGCGAGTCATTGGGATGCATAAACCTAAAGGCGAAATGAAAGTGAAAGTCGGCCTTCGCGCCGATCGAGGGAGGATGGGCCGCGTAACAATGCGGCCCCGCACTCCCGGGGCGTCTCGTTCTCACTGCGAGAAGAGGCG

>HT62 NSTCHUN15

GGAATTCTATATATAATTTTTGCTATCTGATCAGGTATTGTTGGTTCTTCCTTCAGAATACTTATTCGAATGGAACTTAATAGCCCTGGAACTTGAATTAGTAATGATCAGATCTACAACTCTATTGTGACTAGGCATGCATTTCTAATAATTTTTTTTATAGTAATACCTTTTATAATCGGGGGTTTTGGAAATTTTTTAATTCCGCTAATACTAGGTTCTCCTGATATAGCTTTCCCTCGAATGAACAATATTAGCTTCTGACTACTACCTCCTTCACTTATACTGCTAATAACAAATAATTTATTCTTTCCGAATTCAGGAACTGGATGAACTGTGTACCCCCCTCTTTCTCTCTACATATATCATTCATCTCCGTCAGTGGACTTTACTATTTTTTCAATTCATATAACAGGAATTTCATCAATTCTTGGGTCACTTAACTTTATTGTAACAATTTTTATAATAAAGAACTTTTCTTTGAAGTACGATCAAATTAATCTTTTTTCATGATCTATCTCAATCACTGTAATTTTACTAATTCTTTCTCTTCCAGTTCTAGCAGGAGCAATTACTATGCTGTTATTTGATCGAAATTTTAATACTTCCTTCTTTGATCCAATAGGAGGGGGAGACCCGATCCTTTATCAACATTTGTTTATCTGCCCAATGAATTC-----TTTTAATGGCTGCAGTATAACTGACTGTACAAAGGTAGCAT---CAATTGGTTTT---ATGAAATCTGGAATGAAAGGATTAATGAAATATGTACTGTCTCAATTGTACAATTATGAAATTAAAATTTTAATAAAAATGTTAAAATTTACTTATGGGACGATAAGACCCTATAGAATTTTATATTGAAATTACTCAGTAGTTAATTCAGAAATAGTTTCAATATTTGATTGGGAGGATTAT---ACT---CCAACTT--TAATT---GTTAACTTTAATTTAAGG-GTAGATAATGATCTTCAATTTGAAATTGCTAGAATAAATTACCTTAGGGATAACAGCGTAATACTTTTTTATAGGCCATATAGAAAAAAGTGGTTGCGACCTCGATGTTGAATTAGGATAAATTTTAAATGCAGGAGTTTAATAATTAAGTCTGT--------------------------------------------------------------------------------------------------------------------------------------------------------------------------------------------------------------------------------------------------------------------------------------------------------------------------------------------------------------------------------------------------------------------------------------------------------------------------------------------------------------------------------------------------------------------------------------------------------------------------------------

>HT63 NSTCHUN16

GGAATTCTGTATATAATTTTTGCTATCTGATCAGGTATTGTTGGTTCTTCCTTCAGAATACTTATTCGAATGGAACTTAATAGCCCTGGAACTTGAATTAGTAATGATCAGATCTACAACTCTATTGTGACTAGTCATGCATTTCTAATAATTTTTTTTATAGTAATACCTTTTATAATCGGAGGCTTTGGAAATTTTTTAATCCCGTTAATACTAGGTTCCCCTGATATAGCTTTCCCTCGAATAAACAATATTAGCTTCTGACTACTACCTCCTTCACTTATACTGCTAATAACAAATAATCTATTTTTTCCGAACTCAGGAACTGGATGAACTGTATACCCTCCTCTTTCTCTTTACATATATCATCCATCTCCGTCAGTGGACTTTACTATTTTTTCAATTCATATAACAGGAATTTCATCAATTCTTGGGTCACTTAACTTTATTGTAACAATTTTTATAATAAAGAACTTTTCTTTAAAGTACGATCAAATTAATCTTTTTTCATGATCTATCTCAATTACTGTAATTTTACTAATTCTTTCTCTTCCAGTTCTAGCAGGAGCAATTACTATGCTGTTATTTGATCGAAATTTTAATACTTCCTTCTTTGATCCAATAGGAGGGGGAGACCCGATTCTTTATCAACATTTATTTATCTGCCCAATGAATTT-----TTTTAATGGCTGCAGTATAACTGACTGTACAAAGGTAGCAT---CAATTGGTTTT---ATGAAATCTGGAATGAAAGGATTAATGAAATATGTACTGTCTCAATTGTACAATTATGAAATTAAAATTTTAATAAAAATGTTAAAATTCACTTATGGGACGATAAGACCCTATAGAATTTTATATTGAAATTACTCAGTAGTTAATTCAGAAATAGTTTCAATATTTGATTGGGAGGATTAT---ACT---CCAACTT--TAATT---GTTAACTTTAATTTAAGA-GTAGATAATGATCTTCAATTTGAAATTGCTAGAATAAATTACCTTAGGGATAACAGCGTAATACTTTTTTATAGGCCATATAGAAAAAAGTGGTTGCGACCTCGATGTTGAATTAGGATAAATTTTAAATGCAGGAGTTTAATAATTAAGTCTGTCGTCGACGGCGTAGGCTACCGTTGGTGCGCGATGCTCCGGATGGACCCCC-GCGGTTCCAT---CGAGGGCACGCCACCCTCGGATCGAACGCTCCTGCGTCGT---CGTGCACTTCTCCCCTAGTAGAACGTCGCGACCCGTTGTGTGTCGGTCTACGGCCCGAGCGGGAGACTGTCGCGTCGCTTCGGCGCACGCGGCAGACCCTCGGTCGCCCGGCCGGCTGCACGACGGTACACTCACGGTATCGGGCCGCAGCCAATCCATT--CTCGAATGTGTGTGCGTCCATCCCGCCGCAAGCTCGGTCAGTTCTTACCCGGAGGCCACGGACCCAGTGCCGTCCCCGGGCCTGGCCAGCTGTTAGCGGGCGGTGTCCTCGGACCGGCCAAACCTCGGATTACCGGTCGGCGACGCTATTGCTTTGGGTACTCTCAGGACCCGTCTTGAAACACGGACCAAGGAGTCTAACATGTGCGCGAGTCATTGGGATGCATAAACCTAAAGGCGAAATGAAAGTGAAAGTCGGCCTTCGCGCCGATCGAGGGAGGATGGGCCGCGTAACAATGCGGCCCCGCACTCCCGGGGCGTCTCGTTCTCACTGCGAGAAGAGGCG

>HT64 NSTCHUN17

GGAATTCTGTATATAATTTTTGCTATCTGATCAGGTATTGTTGGTTCTTCCTTCAGAATACTTATTCGAATGGAGCTTAATAGTCCTGGAACTTGAATTAATAATGATCAAATTTACAATTCTATTGTAACTAGTCATGCATTTCTAATAATTTTCTTTATAGTTATACCCTTTATAATTGGAGGGTTCGGGAACTTTCTAATTCCACTAATATTGGGTTCCCCTGATATAGCCTTTCCTCGAATAAATAACGTTAGATTTTGATTACTTCCACCCTCAATTATACTACTTATAACAAATAATCTATTTTATCCTAATTCAGGAACTGGATGAACTGTATATCCTCCACTTTCTCTTTATATATATCATTCATCTCCATCTGTAGATTTCACTATTTTTTCAATTCATATAACAGGAATTTCATCAATTCTAGGATCTCTTAATTTTATTGTTACAATTTTTATAATAAAAAATTTCTCTTTGAAGTATGATCAAATTAATCTATTTTCATGATCTATCTCAATTACTGTGATTTTACTAATTCTTTCCCTTCCGGTTCTAGCGGGAGCAATTACTATACTTTTATTTGACCGAAACTTCAACACCTCATTTTTTGATCCAATAGGAGGAGGAGATCCTATTCTATACCAACATCTGTTCATCTGCCCAATGAAGTT-----CTTTAATGGCTGCAGTATAACTGACTGTACAAAGGTAGCAT---CAATTGGTTTT---ATGAAATCTGGAATGAAAGGATTAATGAAATATGTACTGTCTCAATTGTATAATTATGAAATTAAAATTTTAATAAAAATGTTAAAATTTACTTATGGGACGATAAGACCCTATAGAATTTTATATTGAAATTACTCAGTAGTTAGTACAGAAATAATTTTAATATTTGGTTGGGAGGACTAT---ATT---CTAACTT--TAATT---ATTAACTTTAATTTAAGA-GTAAATAAGGATCTTCAATTTGAAATTATCAGAGTAAATTACCTTAGGGATAACAGCGTAATACTTTTTTATAGGCCATATAGAAAAAAGTGATTGCGACCTCGATGTTGAATTAGGATAAATTTTAAATGCAGGAGTTTAATAATTAAGTCTGTCGTCGACGGCGTAGGCTACCGTTGGTGCGCGATGCTCCGGATGGACCCCC-GCGGTTCCAT---CGAGGGCACGCCACCCTCGGATCGAACGCTCCTGCGTCGT---CGTGCACTTCTCCCCTAGTAGAACGTCGCGACCCGTTGTGTGTCGGTCTACGGCCCGAGCGGGAGACTGTCGCGTCGCTTCGGCGCACGCGGCAGACCCTCGGTCGCCCGGCCGGCTGCACGACGGTACACTCACGGTATCGGGCCGCAGCCAATCCATT--CTCGAATGTGTGTGCGTCCATCCCGCCGCAAGCTCGGTCAGTTCTTACCCGGAGGCCACGGACCCAGTGCCGTCCCCGGGCCTGGCCAGCTGTTAGCGGGCGGTGTCCTCGGACCGGCCAAACCTCGGATTACCGGTCGGCGACGCTATTGCTTTGGGTACTCTCAGGACCCGTCTTGAAACACGGACCAAGGAGTCTAACATGTGCGCGAGTCATTGGGATGCATAAACCTAAAGGCGAAATGAAAGTGAAAGTCGGCCTTCGCGCCGATCGAGGGAGGATGGGCCGCGTAACAATGCGGCCCCGCACTCCCGGGGCGTCTCGTTCTCACTGCGAGAAGAGGCG

>HT65 NSTCHUN18

GGAATTCTGTATATAATTTTTGCTATCTGATCAGGTATTGTTGGTTCTTCCTTCAGAATACTTATTCGAATGGAACTTAATAGCCCTGGAACTTGAATTAGTAATGATCAGATCTACAACTCTATTGTGACTAGTCATGCATTTCTAATAATTTTTTTTATAGTAATACCTTTTATAATCGGAGGCTTTGGAAATTTTTTAATCCCGTTAATACTAGGTTCCCCTGATATAGCTTTCCCTCGAATAAACAATATTAGCTTCTGACTACTACCTCCTTCACTTATACTGCTAATAACAAATAATCTATTTTTTCCGAACTCAGGAACTGGATGAACTGTATACCCTCCTCTTTCTCTTTACATATATCATCCATCCCCGTCAGTGGACTTTACTATTTTTTCAATTCATATAACAGGAATTTCATCAATTCTTGGGTCACTTAACTTTATTGTAACAATTTTTATAATAAAGAACTTTTCTTTAAAGTACGATCAAATTAATCTTTTTTCATGATCTATCTCAATTACTGTAATTTTACTAATTCTTTCTCTTCCAGTTCTAGCAGGAGCAATTACTATGCTGTTATTTGATCGAAATTTTAATACTTCCTTCTTTGATCCAATAGGAGGGGGAGACCCGATTCTTTATCAACATTTATTTATCTGCCCAATGAATTT-----TTTTAATGGCTGCAGTATAACTGACTGTACAAAGGTAGCAT---CAATTGGTTTT---ATGAAATCTGGAATGAAAGGATTAATGAAATATGTACTGTCTCAATTGTACAATTATGAAATTAAAATTTTAATAAAAATGTTAAAATTCACTTATGGGACGATAAGACCCTATAGAATTTTATATTGAAATTACTCAGTAGTTAATTCAGAAATAGTTTCAATATTTGATTGGGAGGATTAT---ACT---CCAACTT--TAATT---GTTAACTTTAATTTAAGA-GTAGATAATGATCTTCAATTTGAAATTGCTAGAATAAATTACCTTAGGGATAACAGCGTAATACTTTTTTATAGGCCATATAGAAAAAAGTGGTTGCGACCTCGATGTTGAATTAGGATAAATTTTAAATGCAGGAGTTTAATAATTAAGTCTGTCGTCGACGGCGTAGGCTACCGTTGGTGCGCGATGCTCCGGATGGACCCCC-GCGGTTCCAT---CGAGGGCACGCCACCCTCGGATCGAACGCTCCTGCGTCGT---CGTGCACTTCTCCCCTAGTAGAACGTCGCGACCCGTTGTGTGTCGGTCTACGGCCCGAGCGGGAGACTGTCGCGTCGCTTCGGCGCACGCGGCAGACCCTCGGTCGCCCGGCCGGCTGCACGACGGTACACTCACGGTATCGGGCCGCAGCCAATCCATT--CTCGAATGTGTGTGCGTCCATCCCGCCGCAAGCTCGGTCAGTTCTTACCCGGAGGCCACGGACCCAGTGCCGTCCCCGGGCCTGGCCAGCTGTTAGCGGGCGGTGTCCTCGGACCGGCCAAACCTCGGATTACCGGTCGGCGACGCTATTGCTTTGGGTACTCTCAGGACCCGTCTTGAAACACGGACCAAGGAGTCTAACATGTGCGCGAGTCATTGGGATGCATAAACCTAAAGGCGAAATGAAAGTGAAAGTCGGCCTTCGCGCCGATCGAGGGAGGATGGGCCGCGTAACAATGCGGCCCCGCACTCCCGGGGCGTCTCGTTCTCACTGCGAGAAGAGGCG

>HT66 NSTCHUN19

GGAATTCTGTATATAATTTTTGCTATCTGATCAGGTATTGTTGGTTCTTCCTTCAGAATACTTATTCGAATGGAACTTAATAGCCCTGGAACTTGAATTAGTAATGATCAAATCTACAACTCTATTGTGACTAGTCATGCATTTCTAATAATTTTTTTTAAAGAAATACCTTTTATAATCGGAGGCTTTGGAAATTTTTTAATCCCGTTAATACTAGGTTCCCCTGATATAACTTTCCCTCAAATAAACAATATTAACTTCTGACTACTACCTCCTTCACTTATACTGCTAATAACAAATAATCTATTTTTTCCGAACTCAGGAACTGGATGAACTGTATACCCTCCTCTTTCTCTTTACATATATCATCCATCTCCGTCAGTGGACTTTACTATTTTTTCAATTCATATAACAGGAATTTCATCAATTCTTGGGTCACTTAACTTTATTGTAACAATTTTTATAATAAAGAACTTTTCTTTAAAGTACGATCAAATTAATCTTTTTTCATGATCTATCTCAATTACTGTAATTTTACTAATTCTTTCTCTTCCAGTTCTAGCAGGAGCAATTACTATGCTGTTATTTGATCGAAATTTTAATACTTCCTTCTTTGATCCAATAGGAGGGGGAGACCCGATTCTTTATCAACATTTATTTATCTGCCCAATGAATTT-----TTTTAATGGCTGCAGTATAACTGACTGTACAAAGGTAGCAT---CAATTGGTTTT---ATGAAATCTGGAATGAAAGGATTAATGAAATATGTACTGTCTCAATTGTACAATTATGAAATTAAAATTTTAATAAAAATGTTAAAATTCACTTATGGGACGATAAGACCCTATAGAATTTTATATTGAAATTACTCAGTAGTTAATTCAGAAATAGTTTCAATATTTGATTGGGAGGATTAT---ACT---CCAACTT--TAATT---GTTAACTTTAATTTAAGA-GTAGATAATGATCTTCAATTTGAAATTGCTAGAATAAATTACCTTAGGGATAACAGCGTAATACTTTTTTATAGGCCATATAGAAAAAAGTGGTTGCGACCTCGATGTTGAATTAGGATAAATTTTAAATGCAGGAGTTTAATAATTAAGTCTGTCGTCGACGGCGTAGGCTACCGTTGGTGCGCGATGCTCCGGATGGACCCCC-GCGGTTCCAT---CGAGGGCACGCCACCCTCGGATCGAACGCTCCTGCGTCGT---CGTGCACTTCTCCCCTAGTAGAACGTCGCGACCCGTTGTGTGTCGGTCTACGGCCCGAGCGGGAGACTGTCGCGTCGCTTCGGCGCACGCGGCAGACCCTCGGTCGCCCGGCCGGCTGCACGACGGTACACTCACGGTATCGGGCCGCAGCCAATCCATT--CTCGAATGTGTGTGCGTCCATCCCGCCGCAAGCTCGGTCAGTTCTTACCCGGAGGCCACGGACCCAGTGCCGTCCCCGGGCCTGGCCAGCTGTTAGCGGGCGGTGTCCTCGGACCGGCCAAACCTCGGATTACCGGTCGGCGACGCTATTGCTTTGGGTACTCTCAGGACCCGTCTTGAAACACGGACCAAGGAGTCTAACATGTGCGCGAGTCATTGGGATGCATAAACCTAAAGGCGAAATGAAAGTGAAAGTCGGCCTTCGCGCCGATCGAGGGAGGATGGGCCGCGTAACAATGCGGCCCCGCACTCCCGGGGCGTCTCGTTCTCACTGCGAGAAGAGGCG

>HT67 NSTCHUN20

GGTATTTTATATATAATTTTTGCCCTGTGATCTGGAATTATTGGATCATCTTTTAGTATATTAATTCGTATAGAACTTAATAGACCTGGAATATGAATTAATAATGATCAGATCTATAATTCAATTATTACAAGTCATGCGTTTTTGATAATTTTTTTCATAGTTATGCCTTTTATAATTGGTGGATTTGGGAATTTTCTAGTTCCAATAATACTTGGATCACCTGATATAGCATTTCCTCGAATAAACAATATTAGTTTTTGATTATTACCTCCTTCATTGATATTACTTTTACTCAGAAGATTATTTTTTCCAAGATCAGGAACGGGATGAACAATCTATCCTCCATTATCTTTGTATATGTATCATTCGTCTCCTTCAGTTGATTTTACAATTTTCTCTATTCACATAACTGGAATTTCATCTATTTTAGGATCATTGAATTTCATTGTAACTATTATAATAATAAAAAATTTATCTTTAAACTACGATCAGATTAATTTATTTTCATGATCTATTTCAATTACTGTAATTCTATTAATTTTATCACTTCCTGTTTTAGCTGGAGCAATTACAATATTGTTATTTGACCGAAATTTCAACACTTCTTTCTTTGATCCCATAGGAGGAGGAGATCCTATCTTATATCAACATTTATTT-------------------------------------------------------------------------------------------------------------------------------------------------------------------------------------------------------------------------------------------------------------------------------------------------------------------------------------------------------------------------------------------------------------------------------------------------------------------------------CGTCGACGGCGTAGGCTACCGTTGGTGCGCGATGCTCCGGATGGACCCCC-GCGGTTCCAT---CGAGGGCACGCCACCCTCGGATCGAACGCTCCTGCGTCGT---CGTGCACTTCTCCCCTAGTAGAACGTCGCGACCCGTTGTGTGTCGGTCTACGGCCCGAGCGGGAGACTGTCGCGTCGCTTCGGCGCACGCGGCAGACCCTCGGTCGCCCGGCCGGCTGCACGACGGTACACTCACGGTATCGGGCCGCAGCCAATCCATT--CTCGAATGTGTGTGCGTCCATCCCGCCGCAAGCTCGGTCAGTTCTTACCCGGAGGCCACGGACCCAGTGCCGTCCCCGGGCCTGGCCAGCTGTTAGCGGGCGGTGTCCTCGGACCGGCCAAACCTCGGATTACCGGTCGGCGACGCTATTGCTTTGGGTACTCTCAGGACCCGTCTTGAAACACGGACCAAGGAGTCTAACATGTGCGCGAGTCATTGGGATGCATAAACCTAAAGGCGAAATGAAAGTGAAAGTCGGCCTTCGCGCCGATCGAGGGAGGATGGGCCGCGTAACAATGCGGCCCCGCACTCCCGGGGCGTCTCGTTCTCACTGCGAGAAGAGGCG

>HT70 NARASKN21

GGGATTCTCTATATAATTTTTGCTATCTGGTCAGGCATTGCTGGTTCTTCATTTAGAATACTTATTCGAATAGAACTTAATAGACCTGGGACTTGAATTAGTAATGATCAGATCTACAACTCTATTGTAACTAGGCATGCATTTCTAATAATTTTCTTTATAGTAATGCCTTTTATAATTGGAGGGTTTGGAAACTTTCTAATTCCAATAATACTAGGATCTCCTGATATAGCTTTCCCCCGAATAAACAATATTAGATTTTGACTACTACCCCCTTCACTTATACTGCTAATAACAAATAATTTATTTTTTCCCAATTCAGGAACTGGATGGACTGTATATCCTCCTCTTTCCCTTTACATATACCATTCATCTCCTTCTGTAGACTTTACTATTTTTTCAATTCATATGACAGGAATTTCATCAATTCTCGGGTCACTTAACTTTATTGTAACAATTTTTATAATAAAGAATTTTTCTTTAAAGTACGATCAGATTAATCTTTTTTCATGATCTATCTCTATTACTGTGATTTTACTAATTATTTCTCTCCCAGTTCTAGCAGGAGCAATTACTATGCTGTTATTTGATCGAAATTTTAATACATCATTTTTTGATCCGATAGGAGGAGGAGATCCGATTCTTTATCAACATTTATTTATCTGCCCAATGAATTT-----TTTTAATGGCTGCAGTATAACTGACTGTACAAAGGTAGCAT---CAATTGGTTTT---ATGAAATCTGGAATGAAAGGATTAATGAAATATGTACTGTCTCAATTGTACAACTATGAAATTAAAATTTTAATAAAAATGTTAAAATTCACTTATGGGACGATAAGACCCTATAGAATTTTATATTGAAATTACTCAGTAGTTAATTCAGAAATAATTTCAATATTTGATTGGGAGGATTAT---ATT---TCAACTT--TAATT---GTTAACTTTAATTTAAGA-GTAAATAATGATCTTCAATTTGAAATTGCTAGAATAAATTACCTTAGGGATAACAGCGTAATACTTTTTTATAGGTCATATAGAAAAAAGTGGTTGCGACCTCGATGTTGAATTAGGATAAATTTTAAATGCAGGAGTTTAATAATTAAGTCTGTCGTCGACGGCGTAGGCTACCGTTGGTGCGCGATGCTCCGGATGGACCCCC-GCGGTTCCAT---CGAGGGCACGCCACCCTCGGATCGAACGCTCCTGCGTCGT---CGTGCACTTCTCCCCTAGTAGAACGTCGCGACCCGTTGTGTGTCGGTCTACGGCCCGAGCGGGAGGCTGTCGCGTCGCTTCGGCGCACGCGGCAGACCCTCGGTCGCCCGGCCGGCTGCACGACGGTACACTCACGGTATCGGGCCGCAGCCAATCCATT--CTCGAATGTGTGTGCGTCCATCCCGCCGCAAGCTCGGTCAGTTCTTACCCGGAGGCCACGGACCCAGTGCCGTCCCCGGGCCTGGCCAGCTGTTAGCGGGCGGTGTCCTCGGACCGGCCAAACCTCGGATTACCGGTCGGCGACGCTATTGCTTTGGGTACTCTCAGGACCCGTCTTGAAACACGGACCAAGGAGTCTAACATGTGCGCGAGTCATTGGGATGCATAAACCTAAAGGCGAAATGAAAGTGAAAGTCGGCCTTCGCGCCGATCGAGGGAGGATGGGCCGCGTAACAATGCGGCCCCGCACTCCCGGGGCGTCTCGTTCTCACTGCGAGAAGAGGCG

>HT71 NARASKN22

GGGATTCTCTATATAATTTTTGCTATCTGGTCAGGCATTGCTGGTTCTTCATTTAGAATACTTATTCGAATAGAACTTAATAGACCTGGGACTTGAATTAGTAATGATCAGATCTACAACTCTATTGTAACTAGGCATGCATTTCTAATAATTTTCTTTATAGTAATGCCTTTTATAATTGGAGGGTTTGGAAACTTTCTAATTCCAATAATACTAGGATCTCCTGATATAGCTTTCCCCCGAATAAACAATATTAGATTTTGACTACTACCCCCTTCACTTATACTGCTAATAACAAATAATTTATTTTTTCCCAATTCAGGAACTGGATGGACTGTATATCCTCCTCTTTCCCTTTACATATACCATTCATCTCCTTCTGTAGACTTTACTATTTTTTCAATTCATATGACAGGAATTTCATCAATTCTCGGGTCACTTAACTTTATTGTAACAATTTTTATAATAAAGAATTTTTCTTTAAAGTACGATCAGATTAATCTTTTTTCATGATCTATCTCTATTACTGTGATTTTACTAATTATTTCTCTCCCAGTTCTAGCAGGAGCAATTACTATGCTGTTATTTGATCGAAATTTTAATACATCATTTTTTGATCCGATAGGAGGAGGAGATCCGATTCTTTATCAACATTTATTTATCTGCCCAATGAATTT-----TTTTAATGGCTGCAGTATAACTGACTGTACAAAGGTAGCAT---CAATTGGTTTT---ATGAAATCTGGAATGAAAGGATTAATGAAATATGTACTGTCTCAATTGTACAACTATGAAATTAAAATTTTAATAAAAATGTTAAAATTCACTTATGGGACGATAAGACCCTATAGAATTTTATATTGAAATTACTCAGTAGTTAATTCAGAAATAATTTCAATATTTGATTGGGAGGATTAT---ATT---TCAACTT--TAATT---GTTAACTTTAATTTAAGA-GTAAATAATGATCTTCAATTTGAAATTGCTAGAATAAATTACCTTAGGGATAACAGCGTAATACTTTTTTATAGGTCATATAGAAAAAAGTGGTTGCGACCTCGATGTTGAATTAGGATAAATTTTAAATGCAGGAGTTTAATAATTAAGTCTGTCGTCGACGGCGTAGGCTACCGTTGGTGCGCGATGCTCCGGATGGACCCCC-GCGGTTCCAT---CGAGGGCACGCCACCCTCGGATCGAACGCTCCTGCGTCGT---CGTGCACTTCTCCCCTAGTAGAACGTCGCGACCCGTTGTGTGTCGGTCTACGGCCCGAGCGGGAGACTGTCGCGTCGCTTCGGCGCACGCGGCAGACCCTCGGTCGCCCGGCCGGCTGCACGACGGTACACTCACGGTATCGGGCCGCAGCCAATCCATT--CTCGAATGTGTGTGCGTCCATCCCGCCGCAAGCTCGGTCAGTTCTTACCCGGAGGCCACGGACCCAGTGCCGTCCCCGGGCCTGGCCAGCTGTTAGCGGGCGGTGTCCTCGGACCGGCCAAACCTCGGATTACCGGTCGGCGACGCTATTGCTTTGGGTACTCTCAGGACCCGTCTTGAAACACGGACCAAGGAGTCTAACATGTGCGCGAGTCATTGGGATGCATAAACCTAAAGGCGAAATGAAAGTGAAAGTCGGCCTTCGCGCCGATCGAGGGAGGATGGGCCGCGTAACAATGCGGCCCCGCACTCCCGGGGCGTCTCGTTCTCACTGCGAGAAGAGGCG

>HT72 NARASKN23

GGGATTCTCTATATAATTTTTGCTATCTGGTCAGGCATTGCTGGTTCTTCATTTAGAATACTTATTCGAATAGAACTTAATAGACCTGGGACTTGAATTAGTAATGATCAGATCTACAACTCTATTGTAACTAGGCATGCATTTCTAATAATTTTCTTTATAGTAATGCCTTTTATAATTGGAGGGTTTGGAAACTTTCTAATTCCAATAATACTAGGATCTCCTGATATAGCTTTCCCCCGAATAAACAATATTAGATTTTGACTACTACCCCCTTCACTTATACTGCTAATAACAAATAATTTATTTTTTCCCAATTCAGGAACTGGATGGACTGTATATCCTCCTCTTTCCCTTTACATATACCATTCATCTCCTTCTGTAGACTTTACTATTTTTTCAATTCATATGACAGGAATTTCATCAATTCTCGGGTCACTTAACTTTATTGTAACAATTTTTATAATAAAGAATTTTTCTTTAAAGTACGATCAGATTAATCTTTTTTCATGATCTATCTCTATTACTGTGATTTTACTAATTATTTCTCTCCCAGTTCTAGCAGGAGCAATTACTATGCTGTTATTTGATCGAAATTTTAATACATCATTTTTTGATCCGATAGGAGGAGGAGATCCGATTCTTTATCAACATTTATTTATCTGCCCAATGAATTT-----TTTTAATGGCTGCAGTATAACTGACTGTACAAAGGTAGCAT---CAATTGGTTTT---ATGAAATCTGGAATGAAAGGATTAATGAAATATGTACTGTCTCAATTGTACAACTATGAAATTAAAATTTTAATAAAAATGTTAAAATTCACTTATGGGACGATAAGACCCTATAGAATTTTATATTGAAATTACTCAGTAGTTAATTCAGAAATAATTTCAATATTTGATTGGGAGGATTAT---ATT---TCAACTT--TAATT---GTTAACTTTAATTTAAGA-GTAAATAATGATCTTCAATTTGAAATTGCTAGAATAAATTACCTTAGGGATAACAGCGTAATACTTTTTTATAGGTCATATAGAAAAAAGTGGTTGCGACCTCGATGTTGAATTAGGATAAATTTTAAATGCAGGAGTTTAATAATTAAGTCTGTCGTCGACGGCGTAGGCTACCGTTGGTGCGCGATGCTCCGGATGGACCCCC-GCGGTTCCAT---CGAGGGCACGCCACCCTCGGATCGAACGCTCCTGCGTCGT---CGTGCACTTCTCCCCTAGTAGAACGTCGCGACCCGTTGTGTGTCGGTCTACGGCCCGAGCGGGAGACTGTCGCGTCGCTTCGGCGCACGCGGCAGACCCTCGGTCGCCCGGCCGGCTGCACGACGGTACACTCACGGTATCGGGCCGCAGCCAATCCATT--CTCGAATGTGTGTGCGTCCATCCCGCCGCAAGCTCGGTCAGTTCTTACCCGGAGGCCACGGACCCAGTGCCGTCCCCGGGCCTGGCCAGCTGTTAGCGGGCGGTGTCCTCGGACCGGCCAAACCTCGGATTACCGGTCGGCGACGCTATTGCTTTGGGTACTCTCAGGACCCGTCTTGAAACACGGACCAAGGAGTCTAACATGTGCGCGAGTCATTGGGATGCATAAACCTAAAGGCGAAATGAAAGTGAAAGTCGGCCTTCGCGCCGATCGAGGGAGGATGGGCCGCGTAACAATGCGGCCCCGCACTCCCGGGGCGTCTCGTTCTCACTGCGAGAAGAGGCG

>HT73 NARASKN24

GGGATTCTCTATATAATTTTTGCTATCTGGTCAGGCATTGCTGGTTCTTCATTTAGAATACTTATTCGAATAGAACTTAATAGACCTGGGACTTGAATTAGTAATGATCAGATCTACAACTCTATTGTAACTAGGCATGCATTTCTAATAATTTTCTTTATAGTAATGCCTTTTATAATTGGAGGGTTTGGAAACTTTCTAATTCCAATAATACTAGGATCTCCTGATATAGCTTTCCCCCGAATAAACAATATTAGATTTTGACTACTACCCCCTTCACTTATACTGCTAATAACAAATAATTTATTTTTTCCCAATTCAGGAACTGGATGGACTGTATATCCTCCTCTTTCCCTTTACATATACCATTCATCTCCTTCTGTAGACTTTACTATTTTTTCAATTCATATGACAGGAATTTCATCAATTCTCGGGTCACTTAACTTTATTGTAACAATTTTTATAATAAAGAATTTTTCTTTAAAGTACGATCAGATTAATCTTTTTTCATGATCTATCTCTATTACTGTGATTTTACTAATTATTTCTCTCCCAGTTCTAGCAGGAGCAATTACTATGCTGTTATTTGATCGAAATTTTAATACATCATTTTTTGATCCGATAGGAGGAGGAGATCCGATTCTTTATCAACATTTATTTATCTGCCCAATGAATTT-----TTTTAATGGCTGCAGTATAACTGACTGTACAAAGGTAGCAT---CAATTGGTTTT---ATGAAATCTGGAATGAAAGGATTAATGAAATATGTACTGTCTCAATTGTACAACTATGAAATTAAAATTTTAATAAAAATGTTAAAATTCACTTATGGGACGATAAGACCCTATAGAATTTTATATTGAAATTACTCAGTAGTTAATTCAGAAATAATTTCAATATTTGATTGGGAGGATTAT---ATT---TCAACTT--TAATT---GTTAACTTTAATTTAAGA-GTAAATAATGATCTTCAATTTGAAATTGCTAGAATAAATTACCTTAGGGATAACAGCGTAATACTTTTTTATAGGTCATATAGAAAAAAGTGGTTGCGACCTCGATGTTGAATTAGGATAAATTTTAAATGCAGGAGTTTAATAATTAAGTCTGTCGTCGACGGCGTAGGCTACCGTTGGTGCGCGATGCTCCGGATGGACCCCC-GCGGTTCCAT---CGAGGGCACGCCACCCTCGGATCGAACGCTCCTGCGTCGT---CGTGCACTTCTCCCCTAGTAGAACGTCGCGACCCGTTGTGTGTCGGTCTACGGCCCGAGCGGGAGACTGTCGCGTCGCTTCGGCGCACGCGGCAGACCCTCGGTCGCCCGGCCGGCTGCACGACGGTACACTCACGGTATCGGGCCGCAGCCAATCCATT--CTCGAATGTGTGTGCGTCCATCCCGCCGCAAGCTCGGTCAGTTCTTACCCGGAGGCCACGGACCCAGTGCCGTCCCCGGGCCTGGCCAGCTGTTAGCGGGCGGTGTCCTCGGACCGGCCAAACCTCGGATTACCGGTCGGCGACGCTATTGCTTTGGGTACTCTCAGGACCCGTCTTGAAACACGGACCAAGGAGTCTAACATGTGCGCGAGTCATTGGGATGCATAAACCTAAAGGCGAAATGAAAGTGAAAGTCGGCCTTCGCGCCGATCGAGGGAGGATGGGCCGCGTAACAATGCGGCCCCGCACTCCCGGGGCGTCTCGTTCTCACTGCGAGAAGAGGCG

>HT74 NARASKN25

GGGATTCTCTATATAATTTTTGCTATCTGGTCAGGCATTGCTGGTTCTTCATTTAGAATACTTATTCGAATAGAACTTAATAGACCTGGGACTTGAATTAGTAATGATCAGATCTACAACTCTATTGTAACTAGGCATGCATTTCTAATAATTTTCTTTATAGTAATGCCTTTTATAATTGGAGGGTTTGGAAACTTTCTAATTCCAATAATACTAGGATCTCCTGATATAGCTTTCCCCCGAATAAACAATATTAGATTTTGACTACTACCCCCTTCACTTATACTGCTAATAACAAATAATTTATTTTTTCCCAATTCAGGAACTGGATGGACTGTATATCCTCCTCTTTCCCTTTACATATACCATTCATCTCCTTCTGTAGACTTTACTATTTTTTCAATTCATATGACAGGAATTTCATCAATTCTCGGGTCACTTAACTTTATTGTAACAATTTTTATAATAAAGAATTTTTCTTTAAAGTACGATCAGATTAATCTTTTTTCATGATCTATCTCTATTACTGTGATTTTACTAATTATTTCTCTCCCAGTTCTAGCAGGAGCAATTACTATGCTGTTATTTGATCGAAATTTTAATACATCATTTTTTGATCCGATAGGAGGAGGAGATCCGATTCTTTATCAACATTTATTTATCTGCCCAATGAATTT-----TTTTAATGGCTGCAGTATAACTGACTGTACAAAGGTAGCAT---CAATTGGTTTT---ATGAAATCTGGAATGAAAGGATTAATGAAATATGTACTGTCTCAATTGTACAACTATGAAATTAAAATTTTAATAAAAATGTTAAAATTCACTTATGGGACGATAAGACCCTATAGAATTTTATATTGAAATTACTCAGTAGTTAATTCAGAAATAATTTCAATATTTGATTGGGAGGATTAT---ATT---TCAACTT--TAATT---GTTAACTTTAATTTAAGA-GTAAATAATGATCTTCAATTTGAAATTGCTAGAATAAATTACCTTAGGGATAACAGCGTAATACTTTTTTATAGGTCATATAGAAAAAAGTGGTTGCGACCTCGATGTTGAATTAGGATAAATTTTAAATGCAGGAGTTTAATAATTAAGTCTGTCGTCGACGGCGTAGGCTACCGTTGGTGCGCGATGCTCCGGATGGACCCCC-GCGGTTCCAT---CGAGGGCACGCCACCCTCGGATCGAACGCTCCTGCGTCGT---CGTGCACTTCTCCCCTAGTAGAACGTCGCGACCCGTTGTGTGTCGGTCTACGGCCCGAGCGGGAGGCTGTCGCGTCGCTTCGGCGCACGCGGCAGACCCTCGGTCGCCCGGCCGGCTGCACGACGGTACACTCACGGTATCGGGCCGCAGCCAATCCATT--CTCGAATGTGTGTGCGTCCATCCCGCCGCAAGCTCGGTCAGTTCTTACCCGGAGGCCACGGACCCAGTGCCGTCCCCGGGCCTGGCCAGCTGTTAGCGGGCGGTGTCCTCGGACCGGCCAAACCTCGGATTACCGGTCGGCGACGCTATTGCTTTGGGTACTCTCAGGACCCGTCTTGAAACACGGACCAAGGAGTCTAACATGTGCGCGAGTCATTGGGATGCATAAACCTAAAGGCGAAATGAAAGTGAAAGTCGGCCTTCGCGCCGATCGAGGGAGGATGGGCCGCGTAACAATGCGGCCCCGCACTCCCGGGGCGTCTCGTTCTCACTGCGAGAAGAGGCG

>HT75 NARASKN26

GGGATTCTCTATATAATTTTTGCTATCTGGTCAGGCATTGCTGGTTCTTCATTTAGAATACTTATTCGAATAGAACTTAATAGACCTGGGACTTGAATTAGTAATGATCAGATCTACAACTCTATTGTAACTAGGCATGCATTTCTAATAATTTTCTTTATAGTAATGCCTTTTATAATTGGAGGGTTTGGAAACTTTCTAATTCCAATAATACTAGGATCTCCTGATATAGCTTTCCCCCGAATAAACAATATTAGATTTTGACTACTACCCCCTTCACTTATACTGCTAATAACAAATAATTTATTTTTTCCCAATTCAGGAACTGGATGGACTGTATATCCTCCTCTTTCCCTTTACATATACCATTCATCTCCTTCTGTAGACTTTACTATTTTTTCAATTCATATGACAGGAATTTCATCAATTCTCGGGTCACTTAACTTTATTGTAACAATTTTTATAATAAAGAATTTTTCTTTAAAGTACGATCAGATTAATCTTTTTTCATGATCTATCTCTATTACTGTGATTTTACTAATTATTTCTCTCCCAGTTCTAGCAGGAGCAATTACTATGCTGTTATTTGATCGAAATTTTAATACATCATTTTTTGATCCGATAGGAGGAGGAGATCCGATTCTTTATCAACATTTATTTATCTGCCCAATGAATTT-----TTTTAATGGCTGCAGTATAACTGACTGTACAAAGGTAGCAT---CAATTGGTTTT---ATGAAATCTGGAATGAAAGGATTAATGAAATATGTACTGTCTCAATTGTACAACTATGAAATTAAAATTTTAATAAAAATGTTAAAATTCACTTATGGGACGATAAGACCCTATAGAATTTTATATTGAAATTACTCAGTAGTTAATTCAGAAATAATTTCAATATTTGATTGGGAGGATTAT---ATT---TCAACTT--TAATT---GTTAACTTTAATTTAAGA-GTAAATAATGATCTTCAATTTGAAATTGCTAGAATAAATTACCTTAGGGATAACAGCGTAATACTTTTTTATAGGTCATATAGAAAAAAGTGGTTGCGACCTCGATGTTGAATTAGGATAAATTTTAAATGCAGGAGTTTAATAATTAAGTCTGTCGTCGACGGCGTAGGCTACCGTTGGTGCGCGATGCTCCGGATGGACCCCC-GCGGTTCCAT---CGAGGGCACGCCACCCTCGGATCGAACGCTCCTGCGTCGT---CGTGCACTTCTCCCCTAGTAGAACGTCGCGACCCGTTGTGTGTCGGTCTACGGCCCGAGCGGGAGACTGTCGCGTCGCTTCGGCGCACGCGGCAGACCCTCGGTCGCCCGGCCGGCTGCACGACGGTACACTCACGGTATCGGGCCGCAGCCAATCCATT--CTCGAATGTGTGTGCGTCCATCCCGCCGCAAGCTCGGTCAGTTCTTACCCGGAGGCCACGGACCCAGTGCCGTCCCCGGGCCTGGCCAGCTGTTAGCGGGCGGTGTCCTCGGACCGGCCAAACCTCGGATTACCGGTCGGCGACGCTATTGCTTTGGGTACTCTCAGGACCCGTCTTGAAACACGGACCAAGGAGTCTAACATGTGCGCGAGTCATTGGGATGCATAAACCTAAAGGCGAAATGAAAGTGAAAGTCGGCCTTCGCGCCGATCGGGGGAGGATGGGCCGCGTAACAATGCGGCCCCGCACTCCCGGGGCGTCTCGTTCTCACTGCGAGAAGAGGCG

>HT76 NARASKN27

GGGATTCTCTATATAATTTTTGCTATCTGGTCAGGCATTGCTGGTTCTTCATTTAGAATACTTATTCGAATAGAACTTAATAGACCTGGGACTTGAATTAGTAATGATCAGATCTACAACTCTATTGTAACTAGGCATGCATTTCTAATAATTTTCTTTATAGTAATGCCTTTTATAATTGGAGGGTTTGGAAACTTTCTAATTCCAATAATACTAGGATCTCCTGATATAGCTTTCCCCCGAATAAACAATATTAGATTTTGACTACTACCCCCTTCACTTATACTGCTAATAACAAATAATTTATTTTTTCCCAATTCAGGAACTGGATGGACTGTATATCCTCCTCTTTCCCTTTACATATACCATTCATCTCCTTCTGTAGACTTTACTATTTTTTCAATTCATATGACAGGAATTTCATCAATTCTCGGGTCACTTAACTTTATTGTAACAATTTTTATAATAAAGAATTTTTCTTTAAAGTACGATCAGATTAATCTTTTTTCATGATCTATCTCTATTACTGTGATTTTACTAATTATTTCTCTCCCAGTTCTAGCAGGAGCAATTACTATGCTGTTATTTGATCGAAATTTTAATACATCATTTTTTGATCCGATAGGAGGAGGAGATCCGATTCTTTATCAACATTTATTTATCTGCCCAATGAATTT-----TTTTAATGGCTGCAGTATAACTGACTGTACAAAGGTAGCAT---CAATTGGTTTT---ATGAAATCTGGAATGAAAGGATTAATGAAATATGTACTGTCTCAATTGTACAACTATGAAATTAAAATTTTAATAAAAATGTTAAAATTCACTTATGGGACGATAAGACCCTATAGAATTTTATATTGAAATTACTCAGTAGTTAATTCAGAAATAATTTCAATATTTGATTGGGAGGATTAT---ATT---TCAACTT--TAATT---GTTAACTTTAATTTAAGA-GTAAATAATGATCTTCAATTTGAAATTGCTAGAATAAATTACCTTAGGGATAACAGCGTAATACTTTTTTATAGGTCATATAGAAAAAAGTGGTTGCGACCTCGATGTTGAATTAGGATAAATTTTAAATGCAGGAGTTTAATAATTAAGTCTGTCGTCGACGGCGTAGGCTACCGTTGGTGCGCGATGCTCCGGATGGACCCCC-GCGGTTCCAT---CGAGGGCACGCCACCCTCGGATCGAACGCTCCTGCGTCGT---CGTGCACTTCTCCCCTAGTAGAACGTCGCGACCCGTTGTGTGTCGGTCTACGGCCCGAGCGGGAGACTGTCGCGTCGCTTCGGCGCACGCGGCAGACCCTCGGTCGCCCGGCCGGCTGCACGACGGTACACTCACGGTATCGGGCCGCAGCCAATCCATT--CTCGAATGTGTGTGCGTCCATCCCGCCGCAAGCTCGGTCAGTTCTTACCCGGAGGCCACGGACCCAGTGCCGTCCCCGGGCCTGGCCAGCTGTTAGCGGGCGGTGTCCTCGGACCGGCCAAACCTCGGATTACCGGTCGGCGACGCTATTGCTTTGGGTACTCTCAGGACCCGTCTTGAAACACGGACCAAGGAGTCTAACATGTGCGCGAGTCATTGGGATGCATAAACCTAAAGGCGAAATGAAAGTGAAAGTCGGCCTTCGCGCCGATCGAGGGAGGATGGGCCGCGTAACAATGCGGCCCCGCACTCCCGGGGCGTCTCGTTCTCACTGCGAGAAGAGGCG

>HT77 NARASKN28

GGGATTCTCTATATAATTTTTGCTATCTGGTCAGGCATTGCTGGTTCTTCATTTAGAATACTTATTCGAATAGAACTTAATAGACCTGGGACTTGAATTAGTAATGATCAGATCTACAACTCTATTGTAACTAGGCATGCATTTCTAATAATTTTCTTTATAGTAATGCCTTTTATAATTGGAGGGTTTGGAAACTTTCTAATTCCAATAATACTAGGATCTCCTGATATAGCTTTCCCCCGAATAAACAATATTAGATTTTGACTACTACCCCCTTCACTTATACTGCTAATAACAAATAATTTATTTTTTCCCAATTCAGGAACTGGATGGACTGTATATCCTCCTCTTTCCCTTTACATATACCATTCATCTCCTTCTGTAGACTTTACTATTTTTTCAATTCATATGACAGGAATTTCATCAATTCTCGGGTCACTTAACTTTATTGTAACAATTTTTATAATAAAGAATTTTTCTTTAAAGTACGATCAGATTAATCTTTTTTCATGATCTATCTCTATTACTGTGATTTTACTAATTATTTCTCTCCCAGTTCTAGCAGGAGCAATTACTATGCTGTTATTTGATCGAAATTTTAATACATCATTTTTTGATCCGATAGGAGGAGGAGATCCGATTCTTTATCAACATTTATTTATCTGCCCAATGAATTT-----TTTTAATGGCTGCAGTATAACTGACTGTACAAAGGTAGCAT---CAATTGGTTTT---ATGAAATCTGGAATGAAAGGATTAATGAAATATGTACTGTCTCAATTGTACAACTATGAAATTAAAATTTTAATAAAAATGTTAAAATTCACTTATGGGACGATAAGACCCTATAGAATTTTATATTGAAATTACTCAGTAGTTAATTCAGAAATAATTTCAATATTTGATTGGGAGGATTAT---ATT---TCAACTT--TAATT---GTTAACTTTAATTTAAGA-GTAAATAATGATCTTCAATTTGAAATTGCTAGAATAAATTACCTTAGGGATAACAGCGTAATACTTTTTTATAGGTCATATAGAAAAAAGTGGTTGCGACCTCGATGTTGAATTAGGATAAATTTTAAATGCAGGAGTTTAATAATTAAGTCTGTCGTCGACGGCGTAGGCTACCGTTGGTGCGCGATGCTCCGGATGGACCCCC-GCGGTTCCAT---CGAGGGCACGCCACCCTCGGATCGAACGCTCCTGCGTCGT---CGTGCACTTCTCCCCTAGTAGAACGTCGCGACCCGTTGTGTGTCGGTCTACGGCCCGAGCGGGAGGCTGTCGCGTCGCTTCGGCGCACGCGGCAGACCCTCGGTCGCCCGGCCGGCTGCACGACGGTACACTCACGGTATCGGGCCGCAGCCAATCCATT--CTCGAATGTGTGTGCGTCCATCCCGCCGCAAGCTCGGTCAGTTCTTACCCGGAGGCCACGGACCCAGTGCCGTCCCCGGGCCTGGCCAGCTGTTAGCGGGCGGTGTCCTCGGACCGGCCAAACCTCGGATTACCGGTCGGCGACGCTATTGCTTTGGGTACTCTCAGGACCCGTCTTGAAACACGGACCAAGGAGTCTAACATGTGCGCGAGTCATTGGGATGCATAAACCTAAAGGCGAAATGAAAGTGAAAGTCGGCCTTCGCGCCGATCGAGGGAGGATGGGCCGCGTAACAATGCGGCCCCGCACTCCCGGGGCGTCTCGTTCTCACTGCGAGAAGAGGCG

>HT78 NARASKN29

GGGATTCTCTATATAATTTTTGCTATCTGGTCAGGCATTGCTGGTTCTTCATTTAGAATACTTATTCGAATAGAACTTAATAGACCTGGGACTTGAATTAGTAATGATCAGATCTACAACTCTATTGTAACTAGGCATGCATTTCTAATAATTTTCTTTATAGTAATGCCTTTTATAATTGGAGGGTTTGGAAACTTTCTAATTCCAATAATACTAGGATCTCCTGATATAGCTTTCCCCCGAATAAACAATATTAGATTTTGACTACTACCCCCTTCACTTATACTGCTAATAACAAATAATTTATTTTTTCCCAATTCAGGAACTGGATGGACTGTATATCCTCCTCTTTCCCTTTACATATACCATTCATCTCCTTCTGTAGACTTTACTATTTTTTCAATTCATATGACAGGAATTTCATCAATTCTCGGGTCACTTAACTTTATTGTAACAATTTTTATAATAAAGAATTTTTCTTTAAAGTACGATCAGATTAATCTTTTTTCATGATCTATCTCTATTACTGTGATTTTACTAATTATTTCTCTCCCAGTTCTAGCAGGAGCAATTACTATGCTGTTATTTGATCGAAATTTTAATACATCATTTTTTGATCCGATAGGAGGAGGAGATCCGATTCTTTATCAACATTTATTTATCTGCCCAATGAATTT-----TTTTAATGGCTGCAGTATAACTGACTGTACAAAGGTAGCAT---CAATTGGTTTT---ATGAAATCTGGAATGAAAGGATTAATGAAATATGTACTGTCTCAATTGTACAACTATGAAATTAAAATTTTAATAAAAATGTTAAAATTCACTTATGGGACGATAAGACCCTATAGAATTTTATATTGAAATTACTCAGTAGTTAATTCAGAAATAATTTCAATATTTGATTGGGAGGATTAT---ATT---TCAACTT--TAATT---GTTAACTTTAATTTAAGA-GTAAATAATGATCTTCAATTTGAAATTGCTAGAATAAATTACCTTAGGGATAACAGCGTAATACTTTTTTATAGGTCATATAGAAAAAAGTGGTTGCGACCTCGATGTTGAATTAGGATAAATTTTAAATGCAGGAGTTTAATAATTAAGTCTGTCGTCGACGGCGTAGGCTACCGTTGGTGCGCGATGCTCCGGATGGACCCCC-GCGGTTCCAT---CGAGGGCACGCCACCCTCGGATCGAACGCTCCTGCGTCGT---CGTGCACTTCTCCCCTAGTAGAACGTCGCGACCCGTTGTGTGTCGGTCTACGGCCCGAGCGGGAGACTGTCGCGTCGCTTCGGCGCACGCGGCAGACCCTCGGTCGCCCGGCCGGCTGCACGACGGTACACTCACGGTATCGGGCCGCAGCCAATCCATT--CTCGAATGTGTGTGCGTCCATCCCGCCGCAAGCTCGGTCAGTTCTTACCCGGAGGCCACGGACCCAGTGCCGTCCCCGGGCCTGGCCAGCTGTTAGCGGGCGGTGTCCTCGGACCGGCCAAACCTCGGATTACCGGTCGGCGACGCTATTGCTTTGGGTACTCTCAGGACCCGTCTTGAAACACGGACCAAGGAGTCTAACATGTGCGCGAGTCATTGGGATGCATAAACCTAAAGGCGAAATGAAAGTGAAAGTCGGCCTTCGCGCCGATCGAGGGAGGATGGGCCGCGTAACAATGCGGCCCCGCACTCCCGGGGCGTCTCGTTCTCACTGCGAGAAGAGGCG

>HT79 NARASKN30

GGGATTCTCTATATAATTTTTGCTATCTGGTCAGGCATTGCTGGTTCTTCATTTAGAATACTTATTCGAATAGAACTTAATAGACCTGGGACTTGAATTAGTAATGATCAGATCTACAACTCTATTGTAACTAGGCATGCATTTCTAATAATTTTCTTTATAGTAATGCCTTTTATAATTGGAGGGTTTGGAAACTTTCTAATTCCAATAATACTAGGATCTCCTGATATAGCTTTCCCCCGAATAAACAATATTAGATTTTGACTACTACCCCCTTCACTTATACTGCTAATAACAAATAATTTATTTTTTCCCAATTCAGGAACTGGATGGACTGTATATCCTCCTCTTTCCCTTTACATATACCATTCATCTCCTTCTGTAGACTTTACTATTTTTTCAATTCATATGACAGGAATTTCATCAATTCTCGGGTCACTTAACTTTATTGTAACAATTTTTATAATAAAGAATTTTTCTTTAAAGTACGATCAGATTAATCTTTTTTCATGATCTATCTCTATTACTGTGATTTTACTAATTATTTCTCTCCCAGTTCTAGCAGGAGCAATTACTATGCTGTTATTTGATCGAAATTTTAATACATCATTTTTTGATCCGATAGGAGGAGGAGATCCGATTCTTTATCAACATTTATTTATCTGCCCAATGAATTT-----TTTTAATGGCTGCAGTATAACTGACTGTACAAAGGTAGCAT---CAATTGGTTTT---ATGAAATCTGGAATGAAAGGATTAATGAAATATGTACTGTCTCAATTGTACAACTATGAAATTAAAATTTTAATAAAAATGTTAAAATTCACTTATGGGACGATAAGACCCTATAGAATTTTATATTGAAATTACTCAGTAGTTAATTCAGAAATAATTTCAATATTTGATTGGGAGGATTAT---ATT---TCAACTT--TAATT---GTTAACTTTAATTTAAGA-GTAAATAATGATCTTCAATTTGAAATTGCTAGAATAAATTACCTTAGGGATAACAGCGTAATACTTTTTTATAGGTCATATAGAAAAAAGTGGTTGCGACCTCGATGTTGAATTAGGATAAATTTTAAATGCAGGAGTTTAATAATTAAGTCTGTCGTCGACGGCGTAGGCTACCGTTGGTGCGCGATGCTCCGGATGGACCCCC-GCGGTTCCAT---CGAGGGCACGCCACCCTCGGATCGAACGCTCCTGCGTCGT---CGTGCACTTCTCCCCTAGTAGAACGTCGCGACCCGTTGTGTGTCGGTCTACGGCCCGAGCGGGAGACTGTCGCGTCGCTTCGGCGCACGCGGCAGACCCTCGGTCGCCCGGCCGGCTGCACGACGGTACACTCACGGTATCGGGCCGCAGCCAATCCATT--CTCGAATGTGTGTGCGTCCATCCCGCCGCAAGCTCGGTCAGTTCTTACCCGGAGGCCACGGACCCAGTGCCGTCCCCGGGCCTGGCCAGCTGTTAGCGGGCGGTGTCCTCGGACCGGCCAAACCTCGGATTACCGGTCGGCGACGCTATTGCTTTGGGTACTCTCAGGACCCGTCTTGAAACACGGACCAAGGAGTCTAACATGTGCGCGAGTCATTGGGATGCATAAACCTAAAGGCGAAATGAAAGTGAAAGTCGGCCTTCGCGCCGATCGAGGGAGGATGGGCCGCGTAACAATGCGGCCCCGCACTCCCGGGGCGTCTCGTTCTCACTGCGAGAAGAGGCG

>HT82 NARAWNN31

GGGATTCTCTATATAATTTTTGCTATCTGGTCAGGCATTGCTGGTTCTTCATTTAGAATACTTATTCGAATAGAACTTAATAGACCTGGGACTTGAATTAGTAATGATCAGATCTACAACTCTATTGTAACTAGGCATGCATTTCTAATAATTTTCTTTATAGTAATGCCTTTTATAATTGGAGGGTTTGGAAACTTTCTAATTCCAATAATACTAGGATCTCCTGATATAGCTTTCCCCCGAATAAACAATATTAGATTTTGACTACTACCCCCTTCACTTATACTGCTAATAACAAATAATTTATTTTTTCCCAATTCAGGAACTGGATGGACTGTATATCCTCCTCTTTCCCTTTACATATACCATTCATCTCCTTCTGTAGACTTTACTATTTTTTCAATTCATATGACAGGAATTTCATCAATTCTCGGGTCACTTAACTTTATTGTAACAATTTTTATAATAAAGAATTTTTCTTTAAAGTACGATCAGATTAATCTTTTTTCATGATCTATCTCTATTACTGTGATTTTACTAATTATTTCTCTCCCAGTTCTAGCAGGAGCAATTACTATGCTGTTATTTGATCGAAATTTTAATACATCATTTTTTGATCCGATAGGAGGAGGAGATCCGATTCTTTATCAACATTTATTTATCTGCCCAATGAATTT-----TTTTAATGGCTGCAGTATAACTGACTGTACAAAGGTAGCAT---CAATTGGTTTT---ATGAAATCTGGAATGAAAGGATTAATGAAATATGTACTGTCTCAATTGTACAACTATGAAATTAAAATTTTAATAAAAATGTTAAAATTCACTTATGGGACGATAAGACCCTATAGAATTTTATATTGAAATTACTCAGTAGTTAATTCAGAAATAATTTCAATATTTGATTGGGAGGATTAT---ATT---TCAACTT--TAATT---GTTAACTTTAATTTAAGA-GTAAATAATGATCTTCAATTTGAAATTGCTAGAATAAATTACCTTAGGGATAACAGCGTAATACTTTTTTATAGGTCATATAGAAAAAAGTGGTTGCGACCTCGATGTTGAATTAGGATAAATTTTAAATGCAGGAGTTTAATAATTAAGTCTGTCGTCGACGGCGTAGGCTACCGTTGGTGCGCGATGCTCCGGATGGACCCCC-GCGGTTCCAT---CGAGGGCACGCCACCCTCGGATCGAACGCTCCTGCGTCGT---CGTGCACTTCTCCCCTAGTAGAACGTCGCGACCCGTTGTGTGTCGGTCTACGGCCCGAGCGGGAGACTGTCGCGTCGCTTCGGCGCACGCGGCAGACCCTCGGTCGCCCGGCCGGCTGCACGACGGTACACTCACGGTATCGGGCCGCAGCCAATCCATT--CTCGAATGTGTGTGCGTCCATCCCGCCGCAAGCTCGGTCAGTTCTTACCCGGAGGCCACGGACCCAGTGCCGTCCCCGGGCCTGGCCAGCTGTTAGCGGGCGGTGTCCTCGGACCGGCCAAACCTCGGATTACCGGTCGGCGACGCTATTGCTTTGGGTACTCTCAGGACCCGTCTTGAAACACGGACCAAGGAGTCTAACATGTGCGCGAGTCATTGGGATGCATAAACCTAAAGGCGAAATGAAAGTGAAAGTCGGCCTTCGCGCCGATCGAGGGAGGATGGGCCGCGTAACAATGCGGCCCCGCACTCCCGGGGCGTCTCGTTCTCACTGCGAGAAGAGGCG

>HT83 NARAWNN32

GGGATTCTTTATATAATTTTTGCTATCTGGTCAGGCATTGCTGGTTCTTCATTTAGAATACTTATTCGAATAGAACTTAATAGACCTGGAACTTGAATTAGTAATGATCAGATCTACAACTCTATTGTAACTAGGCATGCATTTCTAATAATTTTCTTTATAGTAATGCCTTTTATAATTGGAGGGTTTGGAAACTTTCTAATTCCAATAATACTAGGATCTCCTGATATAGCTTTCCCCCGAATAAACAATATTAGGTTTTGACTATTACCCCCCTCACTTATACTGCTAATAACAAATAATTTATTTTTTCCCAATTCAGGAACTGGATGGACTGTGTACCCTCCTCTTTCCCTTTACATATACCATTCATCTCCTTCTGTAGACTTTACTATTTTTTCAATTCATATGACAGGAATTTCATCAATTCTTGGGTCACTTAACTTTATTGTAACAATTTTTATAATAAAGAATTTTTCTTTAAAGTACGATCAGATTAATCTTTTTTCATGATCTATCTCGATTACTGTGATTTTACTAATTATTTCTCTCCCAGTTCTAGCAGGAGCAATTACTATGCTGTTATTTGATCGAAATTTTAATACATCATTTTTTGATCCGATAGGAGGAGGAGATCCGATTCTTTATCAACATTTATTTATCTGCCCAATGAATTT-----TTTTAATGGCTGCAGTATAACTGACTGTACAAAGGTAGCAT---CAATTGGTTTT---ATGAAATCTGGAATGAAAGGATTAATGAAATATGTACTGTCTCAATTGTACAACTATGAAATTAAAATTTTAATAAAAATGTTAAAATTCACTTATGGGACGATAAGACCCTATAGAATTTTATATTGAAATTACTCAGTAGTTAATTCAGAAATAATTTCAATATTTGATTGGGAGGATTAT---ATT---TCAACTT--TAATT---GTTAACTTTAATTTAAGA-GTAAATAATGATCTTCAATTTGAAATTGCTAGAATAAATTACCTTAGGGATAACAGCGTAATACTTTTTTATAGGTCATATAGAAAAAAGTGGTTGCGACCTCGATGTTGAATTAGGATAAATTTTAAATGCAGGAGTTTAATAATTAAGTCTGTCGTCGACGGCGTAGGCTACCGTTGGTGCGCGATGCTCCGGATGGACCCCC-GCGGTTCCAT---CGAGGGCACGCCACCCTCGGATCGAACGCTCCTGCGTCGT---CGTGCACTTCTCCCCTAGTAGAACGTCGCGACCCGTTGTGTGTCGGTCTACGGCCCGAGCGGGAGACTGTCGCGTCGCTTCGGCGCACGCGGCAGACCCTCGGTCGCCCGGCCGGCTGCACGACGGTACACTCACGGTATCGGGCCGCAGCCAATCCATT--CTCGAATGTGTGTGCGTCCATCCCGCCGCAAGCTCGGTCAGTTCTTACCCGGAGGCCACGGACCCAGTGCCGTCCCCGGGCCTGGCCAGCTGTTAGCGGGCGGTGTCCTCGGACCGGCCAAACCTCGGATTACCGGTCGGCGACGCTATTGCTTTGGGTACTCTCAGGACCCGTCTTGAAACACGGACCAAGGAGTCTAACATGTGCGCGAGTCATTGGGATGCATAAACCTAAAGGCGAAATGAAAGTGAAAGTCGGCCTTCGCGCCGATCGAGGGAGGATGGGCCGCGTAACAATGCGGCCCCGCACTCCCGGGGCGTCTCGTTCTCACTGCGAGAAGAGGCG

>HT84 NARAWNN33

GGGATTCTCTATATAATTTTTGCTATCTGGTCAGGCATTGCTGGTTCTTCATTTAGAATACTTATTCGAATAGAACTTAATAGACCTGGGACTTGAATTAGTAATGATCAGATCTACAACTCTATTGTAACTAGGCATGCATTTCTAATAATTTTCTTTATAGTAATGCCTTTTATAATTGGAGGGTTTGGAAACTTTCTAATTCCAATAATACTAGGATCTCCTGATATAGCTTTCCCCCGAATAAACAATATTAGATTTTGACTACTACCCCCTTCACTTATACTGCTAATAACAAATAATTTATTTTTTCCCAATTCAGGAACTGGATGGACTGTATATCCTCCTCTTTCCCTTTACATATACCATTCATCTCCTTCTGTAAACTTTACTATTTTTTCAATTCATATGACAGGAATTTCATCAATTCTCGGGTCACTTAACTTTATTGTAACAATTTTTATAATAAAGAATTTTTCTTTAAAGTACGATCAGATTAATCTTTTTTCATGATCTATCTCTATTACTGTGATTTTACTAATTATTTCTCTCCCAGTTCTAGCAGGAGCAATTACTATGCTGTTATTTGATCGAAATTTTAATACATCATTTTTTGATCCGATAGGAGGAGGAGATCCGATTCTTTATCAACATTTATTTATCTGCCCAATGAATTT-----TTTTAATGGCTGCAGTATAACTGACTGTACAAAGGTAGCAT---CAATTGGTTTT---ATGAAATCTGGAATGAAAGGATTAATGAAATATGTACTGTCTCAATTGTACAACTATGAAATTAAAATTTTAATAAAAATGTTAAAATTCACTTATGGGACGATAAGACCCTATAGAATTTTATATTGAAATTACTCAGTAGTTAATTCAGAAATAATTTCAATATTTGATTGGGAGGATTAT---ATT---TCAACTT--TAATT---GTTAACTTTAATTTAAGA-GTAAATAATGATCTTCAATTTGAAATTGCTAGAATAAATTACCTTAGGGATAACAGCGTAATACTTTTTTATAGGTCATATAGAAAAAAGTGGTTGCGACCTCGATGTTGAATTAGGATAAATTTTAAATGCAGGAGTTTAATAATTAAGTCTGTCGTCGACGGCGTAGGCTACCGTTGGTGCGCGATGCTCCGGATGGACCCCC-GCGGTTCCAT---CGAGGGCACGCCACCCTCGGATCGAACGCTCCTGCGTCGT---CGTGCACTTCTCCCCTAGTAGAACGTCGCGACCCGTTGTGTGTCGGTCTACGGCCCGAGCGGGAGACTGTCGCGTCGCTTCGGCGCACGCGGCAGACCCTCGGTCGCCCGGCCGGCTGCACGACGGTACACTCACGGTATCGGGCCGCAGCCAATCCATT--CTCGAATGTGTGTGCGTCCATCCCGCCGCAAGCTCGGTCAGTTCTTACCCGGAGGCCACGGACCCAGTGCCGTCCCCGGGCCTGGCCAGCTGTTAGCGGGCGGTGTCCTCGGACCGGCCAAACCTCGGATTACCGGTCGGCGACGCTATTGCTTTGGGTACTCTCAGGACCCGTCTTGAAACACGGACCAAGGAGTCTAACATGTGCGCGAGTCATTGGGATGCATAAACCTAAAGGCGAAATGAAAGTGAAAGTCGGCCTTCGCGCCGATCGAGGGAGGATGGGCCGCGTAACAATGCGGCCCCGCACTCCCGGGGCGTCTCGTTCTCACTGCGAGAAGAGGCG

>HT85 NARAWNN34

GGGATTCTTTATATAATTTTTGCTATCTGGTCAGGCATTGCTGGTTCTTCATTTAGAATACTTATTCGAATAGAACTTAATAGACCTGGAACTTGAATTAGTAATGATCAGATCTACAACTCTATTGTAACTAGGCATGCATTTCTAATAATTTTCTTTATAGTAATGCCTTTTATAATTGGAGGGTTTGGAAACTTTCTAATTCCAATAATACTAGGATCTCCTGATATAGCTTTCCCCCGAATAAACAATATTAGGTTTTGACTATTACCCCCCTCACTTATACTGCTAATAACAAATAATTTATTTTTTCCCAATTCAGGAACTGGATGGACTGTGTACCCTCCTCTTTCCCTTTACATATACCATTCATCTCCTTCTGTAGACTTTACTATTTTTTCAATTCATATGACAGGAATTTCATCAATTCTTGGGTCACTTAACTTTATTGTAACAATTTTTATAATAAAGAATTTTTCTTTAAAGTACGATCAGATTAATCTTTTTTCATGATCTATCTCGATTACTGTGATTTTACTAATTATTTCTCTCCCAGTTCTAGCAGGAGCAATTACTATGCTGTTATTTGATCGAAATTTTAATACATCATTTTTTGATCCGATAGGAGGAGGAGATCCGATTCTTTATCAACATTTATTTATCTGCCCAATGAATTT-----TTTTAATGGCTGCAGTATAACTGACTGTACAAAGGTAGCAT---CAATTGGTTTT---ATGAAATCTGGAATGAAAGGATTAATGAAATATGTACTGTCTCAATTGTACAACTATGAAATTAAAATTTTAATAAAAATGTTAAAATTCACTTATGGGACGATAAGACCCTATAGAATTTTATATTGAAATTACTCAGTAGTTAATTCAGAAATAATTTCAATATTTGATTGGGAGGATTAT---ATT---TCAACTT--TAATT---GTTAACTTTAATTTAAGA-GTAAATAATGATCTTCAATTTGAAATTGCTAGAATAAATTACCTTAGGGATAACAGCGTAATACTTTTTTATAGGTCATATAGAAAAAAGTGGTTGCGACCTCGATGTTGAATTAGGATAAATTTTAAATGCAGGAGTTTAATAATTAAGTCTGTCGTCGACGGCGTAGGCTACCGTTGGTGCGCGATGCTCCGGATGGACCCCC-GCGGTTCCAT---CGAGGGCACGCCACCCTCGGATCGAACGCTCCTGCGTCGT---CGTGCACTTCTCCCCTAGTAGAACGTCGCGACCCGTTGTGTGTCGGTCTACGGCCCGAGCGGGAGACTGTCGCGTCGCTTCGGCGCACGCGGCAGACCCTCGGTCGCCCGGCCGGCTGCACGACGGTACACTCACGGTATCGGGCCGCAGCCAATCCATT--CTCGAATGTGTGTGCGTCCATCCCGCCGCAAGCTCGGTCAGTTCTTACCCGGAGGCCACGGACCCAGTGCCGTCCCCGGGCCTGGCCAGCTGTTAGCGGGCGGTGTCCTCGGACCGGCCAAACCTCGGATTACCGGTCGGCGACGCTATTGCTTTGGGTACTCTCAGGACCCGTCTTGAAACACGGACCAAGGAGTCTAACATGTGCGCGAGTCATTGGGATGCATAAACCTAAAGGCGAAATGAAAGTGAAAGTCGGCCTTCGCGCCGATCGAGGGAGGATGGGCCGCGTAACAATGCGGCCCCGCACTCCCGGGGCGTCTCGTTCTCACTGCGAGAAGAGGCG

>HT87 NARAWNN35

GGGATTCTTTATATAATTTTTGCTATCTGGTCAGGCATTGCTGGTTCTTCATTTAGAATACTTATCCGAATAGAGCTTAATAGACCTGGGACTTGAATTAGTAACGATCAGATCTACAACTCTATTGTAACTAGGCACGCATTTCTAATAATTTTCTTTATAGTAATGCCTTTTATAATTGGAGGGTTTGGAAACTTTCTAATTCCAATAATACTAGGATCTCCTGATATAGCTTTCCCCCGAATAAACAATATTAGGTTTTGACTATTACCTCCTTCACTTATACTTCTAATAACAAATAATTTATTTTTTCCCAATTCAGGAACTGGATGGACTGTGTACCCTCCTCTTTCCCTTTACATATACCATTCATCTCCTTCTGTAGACTTTACTATTTTTTCAATTCATATGACAGGAATTTCATCAATTCTTGGGTCACTTAACTTTATTGTAACAATTTTTATAATAAAGAATTTTTCTTTAAAGTACGATCAGATTAATCTTTTTTCATGATCTATCTCGATTACTGTGATTTTACTAATTATTTCTCTCCCAGTTCTAGCTGGAGCAATTACTATGCTGTTATTTGATCGAAATTTTAATACATCATTTTTTGATCCGATAGGAGGAGGAGATCCGATTCTTTATCAACATTTATTCATCTGCCCAATGAATTT-----TTTTAATGGCTGCAGTATAACTGACTGTACAAAGGTAGCAT---CAATTGGTTTT---ATGAAATCTGGAATGAAAGGATTAATGAAATATGTACTGTCTCAATTGTACAACTATGAAATTAAAATTTTAATAAAAATGTTAAAATTCACTTATGGGACGATAAGACCCTATAGAATTTTATATTGAAATTACTCAGTAGTTAATTCAGAAATAATTTCAATATTTGATTGGGAGGATTAT---ATT---TCAACTT--TAATT---GTTAACTTTAATTTAAGA-GTAAATAATGATCTTCAATTTGAAATTGCTAGAATAAATTACCTTAGGGATAACAGCGTAATACTTTTTTATAGGTCATATAGAAAAAAGTGGTTGCGACCTCGATGTTGAATTAGGATAAATTTTAAATGCAGGAGTTTAATAATTAAGTCTGTCGTCGACGGCGTAGGCTACCGTTGGTGCGCGATGCTCCGGATGGACCCCC-GCGGTTCCAT---CGAGGGCACGCCACCCTCGGATCGAACGCTCCTGCGTCGT---CGTGCACTTCTCCCCTAGTAGAACGTCGCGACCCGTTGTGTGTCGGTCTACGGCCCGAGCGGGAGACTGTCGCGTCGCTTCGGCGCACGCGGCAGACCCTCGGTCGCCCGGCCGGCTGCACGACGGTACACTCACGGTATCGGGCCGCAGCCAATCCATT--CTCGAATGTGTGTGCGTCCATCCCGCCGCAAGCTCGGTCAGTTCTTACCCGGAGGCCACGGACCCAGTGCCGTCCCCGGGCCTGGCCAGCTGTTAGCGGGCGGTGTCCTCGGACCGGCCAAACCTCGGATTACCGGTCGGCGACGCTATTGCTTTGGGTACTCTCAGGACCCGTCTTGAAACACGGACCAAGGAGTCTAACATGTGCGCGAGTCATTGGGATGCATAAACCTAAAGGCGAAATGAAAGTGAAAGTCGGCCTTCGCGCCGATCGAGGGAGGATGGGCCGCGTAACAATGCGGCCCCGCACTCCCGGGGCGTCTCGTTCTCACTGCGAGAAGAGGCG

>HT93 NARATBC40

GGGATTCTGTATATAATTTTTGCTATCTGATCAGGTATTGCTGGTTCTTCCTTCAGAATACTTATTCGAATGGAACTTAATAGCCCTGGAACTTGAATTAGTAATGATCAGATCTACAACTCTATTGTAACTAGGCATGCATTTCTGATAATTTTTTTTATAGTAATACCTTTTATAATTGGAGGGTTTGGAAATTTTTTGATTCCACTAATACTAGGATCTCCTGATATAACTTTTCCTCGAATAAACAATATTAGCTTTTGACTTCTACCTCCTTCACTTATACTGCTAATAACAAATAATTTATTTTTTCCAAATTCAGGAACTGGATGAACTGTGTACCCTCCTCTTTCTCTCTACATATACCATTCATCCCCTTCAGTAGACTTTACTATTTTTTCAATCCATATAACAGGAATTTCATCAATTCTTGGATCACTTAACTTTATTGTAACAATTTTTATAATAAAGAATTTTTCTTTAAAGTACGATCAAATTAATCTTTTTTCATGATCTATCTCAATTACTGTAATTTTACTAATCCTTTCTCTCCCAGTTCTAGCGGGAGCAATTACTATGCTGTTATTTGACCGAAATTTTAACACTTCCTTCTTTGATCCAATAGGAGGGGGAGATCCAATTCTTTATCAACATCTATTTATCTGCCCAATGAATTT-----TTTTAATGGCTGCAGTATAACTGACTGTACAAAGGTAGCAT---CAATTGGTTTT---ATGAAATCTGGAATGAAAGGATTAATGAAATATGTACTGTCTCAATTGTACAATTATGAAATTAAAATTTTAATAAAAATGTTAAAATTCACTTATGGGACGATAAGACCCTATAGAATTTTATATTGAAATTACTCAGTAGTTAATTCAGAAATAGTTTCAATATTTGATTGGGAGGATTAT---AAT---CCAACTT--TAATT---GTTAACTTTAATTTAAGA-GTAGATAATGATCTTCAATTTGAAATTGCTAGAATAAATTACCTTAGGGATAACAGCGTAATACTTTTTTATAGGCCATATAGAAAAAAGTGGTTGCGACCTCGATGTTGAATTAGGATAAATTTTAAATGCAGGAGTTTAATAATTAAGTCTGTCGTCGACGGCGTAGGCTACCGTTGGTGCGCGATGCTCCGGATGGACCCCC-GCGGTTCCAT---CGAGGGCACGCCACCCTCGGATCGAACGCTCCTGCGTCGT---CGTGCACTTCTCCCCTAGTAGAACGTCGCGACCCGTTGTGTGTCGGTCTACGGCCCGAGCGGGAGACTGTCGCGTCGCTTCGGCGCACGCGGCAGACCCTCGGTCGCCCGGCCGGCTGCACGACGGTACACTCACGGTATCGGGCCGCAGCCAATCCATT--CTCGAATGTGTGTGCGTCCATCCCGCCGCAAGCTCGGTCAGTTCTTACCCGGAGGCCACGGACCCAGTGCCGTCCCCGGGCCTGGCCAGCTGTTAGCGGGCGGTGTCCTCGGACCGGCCAAACCTCGGATTACCGGTCGGCGACGCTATTGCTTTGGGTACTCTCAGGACCCGTCTTGAAACACGGACCAAGGAGTCTAACATGTGCGCGAGTCATTGGGATGCATAAACCTAAAGGCGAAATGAAAGTGAAAGTCGGCCTTCGCGCCGATCGAGGGAGGATGGGCCGCGTAACAATGCGGCCCCGCACTCCCGGGGCGTCTCGTTCTCACTGCGAGAAGAGGCG

>HT94 NARATBC41

GGAATTCTGTATATAATTTTTGCTATCTGATCAGGTATTGTTGGTTCTTCCTTCAGAATACTTATTCGAATGGAACTTAATAGCCCTGGAACTTGAATTAGTAATGATCAGATCTACAACTCTATTGTGACTAGGCATGCATTTCTAATAATTTTTTTTATAGTAATACCTTTTATAATCGGAGGTTTTGGAAATTTTTTAATTCCGCTAATACTAGGTTCTCCTGATATAGCTTTCCCCCGAATGAACAATATCAGCTTCTGACTACTACCTCCTTCACTTATACTGCTAATAACAAATAACTTATTCTTTCCGAATTCAGGAACTGGATGAACTGTATACCCCCCTCTTTCTCTCTACATATATCATTCATCTCCGTCAGTGGACTTTACTATTTTTTCAATTCATATAACAGGAATTTCATCAATTCTTGGGTCACTTAACTTTATTGTAACAATTTTTATAATAAAGAACTTTTCTTTGAAGTACGACCAAATTAATCTTTTTTCATGATCTATCTCAATTACTGTAATTTTACTAATTCTTTCTCTTCCAGTTCTAGCAGGAGCAATTACTATGCTGTTATTTGATCGAAATTTTAATACTTCCTTCTTTGATCCAATAGGAGGGGGAGACCCGATTCTTTATCAACATTTATTTATCTGCCCAATGAATTC-----TTTTAATGGCTGCAGTATAACTGACTGTACAAAGGTAGCAT---CAATTGGTTTT---ATGAAATCTGGAATGAAAGGATTAATGAAATATGTACTGTCTCAATTGTACAATTATGAAATTAAAATTTTAATAAAAATGTTAAAATTCACTTATGGGACGATAAGACCCTATAGAATTTTATATTGAAATTACTCAGTAGTTAATTCAGAAATAGTTTCAATATTTGATTGGGAGGATTAT---ACT---CCAACTT--TAATT---GTTAACTTTAATTTAAGA-GTAGATAATGATCTTCAATTTGAAATTGCTAGAATAAATTACCTTAGGGATAACAGCGTAATACTTTTTTATAGGCCATATAGAAAAAAGTGGTTGCGACCTCGATGTTGAATTAGGATAAATTTTAAATGCAGGAGTTTAATAATTAAGTCTGTCGTCGACGGCGTAGGCTACCGTTGGTGCGCGATGCTCCGGATGGACCCCC-GCGGTTCCAT---CGAGGGCACGCCACCCTCGGATCGAACGCTCCTGCGTCGT---CGTGCACTTCTCCCCTAGTAGAACGTCGCGACCCGTTGTGTGTCGGTCTACGGCCCGAGCGGGAGGCTGTCGCGTCGCTTCGGCGCACGCGGCAGACCCTCGGTCGCCCGGCCGGCTGCACGACGGTACACTCACGGTATCGGGCCGCAGCCAATCCATT--CTCGAATGTGTGTGCGTCCATCCCGCCGCAAGCTCGGTCAGTTCTTACCCGGAGGCCACGGACCCAGTGCCGTCCCCGGGCCTGGCCAGCTGTTAGCGGGCGGTGTCCTCGGACCGGCCAAACCTCGGATTACCGGTCGGCGACGCTATTGCTTTGGGTACTCTCAGGACCCGTCTTGAAACACGGACCAAGGAGTCTAACATGTGCGCGAGTCATTGGGATGCATAAACCTAAAGGCGAAATGAAAGTGAAAGTCGGCCTTCGCGCCGATCGAGGGAGGATGGGCCGCGTAACAATGCGGCCCCGCACTCCCGGGGCGTCTCGTTCTCACTGCGAGAAGAGGCG

>HT95 NARATBC42

GGGATTCTTTATATAATTTTTGCTATCTGGTCAGGCATTGCTGGTTCTTCATTTAGAATACTTATTCGAATAGAACTTAATAGACCTGGGACTTGAATTAGTAATGATCAGATCTACAACTCCATTGTAACTAGGCATGCATTTCTAATAATTTTCTTTATAGTAATGCCTTTTATAATTGGAGGGTTTGGAAACTTTCTAATTCCAATAATACTAGGATCTCCTGATATAGCTTTCCCCCGAATAAACAATATTAGGTTTTGACTATTACCCCCCTCACTTATACTGCTAATAACAAATAATTTATTTTTTCCCAATTCAGGAACTGGATGGACTGTGTACCCTCCTCTTTCCCTTTACATATACCATTCATCTCCTTCTGTAGACTTTACTATTTTTTCAATTCATATGACAGGAATTTCGTCAATTCTTGGGTCACTTAACTTTATTGTAACAATTTTTATAATAAAGAATTTTTCTTTAAAGTACGATCAGATTAATCTTTTTTCATGATCTATCTCGATTACTGTGATTTTACTAATTATTTCTCTCCCAGTTCTAGCAGGAGCAATTACTATGCTGTTATTTGATCGAAATTTTAATACATCATTTTTTGATCCGATAGGAGGAGGAGATCCGATTCTTTATCAACATTTATTTATCTGCCCAATGAATTT-----TTTTAATGGCTGCAGTATAACTGACTGTACAAAGGTAGCAT---CAATTGGTTTT---ATGAAATCTGGAATGAAAGGATTAATGAAATATGTACTGTCTCAATTGTACAACTATGAAATTAAAATTTTAATAAAAATGTTAAAATTCACTTATGGGACGATAAGACCCTATAGAATTTTATATTGAAATTACTCAGTAGTTAATTCAGAAATAATTTCAATATTTGATTGGGAGGATTAT---ATT---TCAACTT--TAATT---GTTAACTTTAATTTAAGA-GTAAATAATGATCTTCAATTTGAAATTGCTAGAATAAATTACCTTAGGGATAACAGCGTAATACTTTTTTATAGGTCATATAGAAAAAAGTGGTTGCGACCTCGATGTTGAATTAGGATAAATTTTAAATGCAGGAGTTTAATAATTAAGTCTGTCGTCGACGGCGTAGGCTACCGTTGGTGCGCGATGCTCCGGATGGACCCCC-GCGGTTCCAT---CGAGGGCACGCCACCCTCGGATCGAACGCTCCTGCGTCGT---CGTGCACTTCTCCCCTAGTAGAACGTCGCGACCCGTTGTGTGTCGGTCTACGGCCCGAGCGGGAGACTGTCGCGTCGCTTCGGCGCACGCGGCAGACCCTCGGTCGCCCGGCCGGCTGCACGACGGTACACTCACGGTATCGGGCCGCAGCCAATCCATT--CTCGAATGTGTGTGCGTCCATCCCGCCGCAAGCTCGGTCAGTTCTTACCCGGAGGCCACGGACCCAGTGCCGTCCCCGGGCCTGGCCAGCTGTTAGCGGGCGGTGTCCTCGGACCGGCCAAACCTCGGATTACCGGTCGGCGACGCTATTGCTTTGGGTACTCTCAGGACCCGTCTTGAAACACGGACCAAGGAGTCTAACATGTGCGCGAGTCATTGGGATGCATAAACCTAAAGGCGAAATGAAAGTGAAAGTCGGCCTTCGCGCCGATCGAGGGAGGATGGGCCGCGTAACAATGCGGCCCCGCACTCCCGGGGCGTCTCGTTCTCACTGCGAGAAGAGGCG

>HT96 NARATBC43

GGGATTCTGTATATAATTTTTGCTATCTGATCAGGTATTGCTGGTTCTTCCTTCAGAATACTTATTCGAATGGAACTTAATAGCCCTGGAACTTGAATTAGTAATGATCAGATCTACAACTCTATTGTAACTAGGCATGCATTTCTGATAATTTTTTTTATAGTAATACCTTTTATAATTGGAGGGTTTGGAAATTTTTTGATTCCACTAATACTAGGATCTCCTGATATAGCTTTTCCTCGAATAAACAATATTAGCTTTTGACTTCTACCTCCTTCACTTATACTGCTAATAACAAATAATTTATTTTTTCCAAATTCAGGAACTGGATGAACTGTGTACCCTCCTCTTTCTCTCTACATATACCATTCATCCCCTTCAGTAGACTTTACTATTTTTTCAATCCATATAACAGGAATTTCATCAATTCTTGGATCACTTAACTTTATTGTAACAATTTTTATAATAAAGAATTTTTCTTTAAAGTACGATCAAATTAATCTTTTTTCATGATCTATCTCAATTACTGTAATTTTACTAATCCTTTCTCTCCCAGTTCTAGCGGGAGCAATTACTATGCTGTTATTTGACCGAAATTTTAACACTTCCTTCTTTGATCCAATAGGAGGGGGAGATCCAATTCTTTATCAACATCTATTTATCTGCCCAATGAATTT-----TTTTAATGGCTGCAGTATAACTGACTGTACAAAGGTAGCAT---CAATTGGTTTT---ATGAAATCTGGAATGAAAGGATTAATGAAATATGTACTGTCTCAATTGTACAATTATGAAATTAAAATTTTAATAAAAATGTTAAAATTCACTTATGGGACGATAAGACCCTATAGAATTTTATATTGAAATTACTCAGTAGTTAATTCAGAAATAGTTTCAATATTTGATTGGGAGGATTAT---AAT---CCAACTT--TAATT---GTTAACTTTAATTTAAGA-GTAGATAATGATCTTCAATTTGAAATTGCTAGAATAAATTACCTTAGGGATAACAGCGTAATACTTTTTTATAGGCCATATAGAAAAAAGTGGTTGCGACCTCGATGTTGAATTAGGATAAATTTTAAATGCAGGAGTTTAATAATTAAGTCTGTCGTCGACGGCGTAGGCTACCGTTGGTGCGCGATGCTCCGGATGGACCCCC-GCGGTTCCAT---CGAGGGCACGCCACCCTCGGATCGAACGCTCCTGCGTCGT---CGTGCACTTCTCCCCTAGTAGAACGTCGCGACCCGTTGTGTGTCGGTCTACGGCCCGAGCGGGAGACTGTCGCGTCGCTTCGGCGCACGCGGCAGACCCTCGGTCGCCCGGCCGGCTGCACGACGGTACACTCACGGTATCGGGCCGCAGCCAATCCATT--CTCGAATGTGTGTGCGTCCATCCCGCCGCAAGCTCGGTCAGTTCTTACCCGGAGGCCACGGACCCAGTGCCGTCCCCGGGCCTGGCCAGCTGTTAGCGGGCGGTGTCCTCGGACCGGCCAAACCTCGGATTACCGGTCGGCGACGCTATTGCTTTGGGTACTCTCAGGACCCGTCTTGAAACACGGACCAAGGAGTCTAACATGTGCGCGAGTCATTGGGATGCATAAACCTAAAGGCGAAATGAAAGTGAAAGTCGGCCTTCGCGCCGATCGAGGGAGGATGGGCCGCGTAACAATGCGGCCCCGCACTCCCGGGGCGTCTCGTTCTCACTGCGAGAAGAGGCG

>HT97 NARATBC44

GGGATTCTGTATATAATTTTTGCTATCTGATCAGGTATTGCTGGTTCTTCCTTCAGAATACTTATTCGAATGGAACTTAATAGCCCTGGAACTTGAATTAGTAATGATCAGATCTACAACTCTATTGTAACTAGGCATGCATTTCTGATAATTTTTTTTATAGTAATACCTTTTATGATTGGAGGGTTTGGAAATTTTTTGATTCCACTAATACTAGGATCTCCTGATATAGCTTTTCCTCGAATAAACAATATTAGCTTTTGACTTCTACCTCCTTCACTTATACTGCTAATAACAAATAACTTATTTTTTCCAAATTCAGGAACTGGATGAACTGTGTACCCTCCTCTTTCTCTCTACATATACCATTCATCCCCTTCAGTAGACTTTACTATTTTTTCAATCCATATAACAGGGATTTCATCAATTCTTGGATCACTTAACTTTATTGTAACAATTTTTATAATAAAGAATTTTTCTTTAAAGTACGATCAAATTAATCTTTTTTCATGATCTATCTCAATTACTGTAATTTTACTAATTCTTTCTCTTCCAGTTCTAGCGGGAGCAATTACTATGCTGTTATTTGACCGAAATTTTAACACCTCCTTCTTTGATCCAATAGGAGGGGGAGATCCAATTCTTTATCAACATCTATTTATCTGCCCAATGAATTT-----TTTTAATGGCTGCAGTATAACTGACTGTACAAAGGTAGCAT---CAATTGGTTTT---ATGAAATCTGGAATGAAAGGATTAATGAAATATGTACTGTCTCAATTGTACAATTATGAAATTAAAATTTTAATAAAAATGTTAAAATTCACTTATGGGACGATAAGACCCTATAGAATTTTATATTGAAATTACTCAGTAGTTAATTCAGAAATAGTTTCAATATTTGATTGGGAGGATTAT---AAT---CCAACTT--TAATT---GTTAACTTTAATTTAAGA-GTAGATAATGATCTTCAATTTGAAATTGCTAGAATAAATTACCTTAGGGATAACAGCGTAATACTTTTTTATAGGCCATATAGAAAAAAGTGGTTGCGACCTCGATGTTGAATTAGGATAAATTTTAAATGCAGGAGTTTAATAATTAAGTCTGTCGTCGACGGCGTAGGCTACCGTTGGTGCGCGATGCTCCGGATGGACCCCC-GCGGTTCCAT---CGAGGGCACGCCACCCTCGGATCGAACGCTCCTGCGTCGT---CGTGCACTTCTCCCCTAGTAGAACGTCGCGACCCGTTGTGTGTCGGTCTACGGCCCGAGCGGGAGGCTGTCGCGTCGCTTCGGCGCACGCGGCAGACCCTCGGTCGCCCGGCCGGCTGCACGACGGTACACTCACGGTATCGGGCCGCAGCCAATCCATT--CTCGAATGTGTGTGCGTCCATCCCGCCGCAAGCTCGGTCAGTTCTTACCCGGAGGCCACGGACCCAGTGCCGTCCCCGGGCCTGGCCAGCTGTTAGCGGGCGGTGTCCTCGGACCGGCCAAACCTCGGATTACCGGTCGGCGACGCTATTGCTTTGGGTACTCTCAGGACCCGTCTTGAAACACGGACCAAGGAGTCTAACATGTGCGCGAGTCATTGGGATGCATAAACCTAAAGGCGAAATGAAAGTGAAAGTCGGCCTTCGCGCCGATCGAGGGAGGATGGGCCGCGTAACAATGCGGCCCCGCACTCCCGGGGCGTCTCGTTCTCACTGCGAGAAGAGGCG

>HT98 NARATBC45

GGAATTCTGTATATAATTTTTGCTATCTGATCAGGTATTGTTGGTTCTTCCTTCAGAATACTTATTCGAATGGAACTTAATAGCCCTGGAACTTGAATTAGTAATGATCAGATCTACAACTCTATTGTGACTAGGCATGCATTTCTAATAATTTTTTTTATAGTAATACCTTTTATAATCGGAGGTTTTGGAAATTTTTTAATTCCGCTAATACTAGGTTCTCCTGATATAGCTTTCCCCCGAATGAACAATATCAGCTTCTGACTACTACCTCCTTCACTTATACTGCTAATAACAAATAATTTATTCTTTCCGAATTCAGGAACTGGATGAACTGTATACCCCCCTCTTTCTCTCTACATATATCATTCATCTCCGTCAGTGGACTTTACTATTTTTTCAATTCATATAACGGGAATTTCATCAATTCTTGGGTCACTTAACTTTATTGTAACAATTTTTATAATAAAGAACTTTTCTTTGAAGTACGACCAAATTAATCTTTTTTCATGATCTATCTCAATTACTGTAATTTTACTAATTCTTTCTCTTCCAGTTCTAGCAGGAGCAATTACTATGCTGTTATTTGATCGAAATTTTAATACTTCCTTCTTTGATCCAATAGGAGGGGGAGACCCGATTCTTTATCAACATTTATTTATCTGCCCAATGAATTC-----TTTTAATGGCTGCAGTATAACTGACTGTACAAAGGTAGCAT---CAATTGGTTTT---ATGAAATCTGGAATGAAAGGATTAATGAAATATGTACTGTCTCAATTGTACAATTATGAAATTAAAATTTTAATAAAAATGTTAAAATTCACTTATGGGACGATAAGACCCTATAGAATTTTATATTGAAATTACTCAGTAGTTAATTCAGAAATAGTTTCAATATTTGATTGGGAGGATTAT---ACT---CCAACTT--TAATT---GTTAACTTTAATTTAAGA-GTAGATAATGATCTTCAATTTGAAATTGCTAGAATAAATTACCTTAGGGATAACAGCGTAATACTTTTTTATAGGCCATATAGAAAAAAGTGGTTGCGACCTCGATGTTGAATTAGGATAAATTTTAAATGCAGGAGTTTAATAATTAAGTCTGTCGTCGACGGCGTAGGCTACCGTTGGTGCGCGATGCTCCGGATGGACCCCC-GCGGTTCCAT---CGAGGGCACGCCACCCTCGGATCGAACGCTCCTGCGTCGT---CGTGCACTTCTCCCCTAGTAGAACGTCGCGACCCGTTGTGTGTCGGTCTACGGCCCGAGCGGGAGACTGTCGCGTCGCTTCGGCGCACGCGGCAGACCCTCGGTCGCCCGGCCGGCTGCACGACGGTACACTCACGGTATCGGGCCGCAGCCAATCCATT--CTCGAATGTGTGTGCGTCCATCCCGCCGCAAGCTCGGTCAGTTCTTACCCGGAGGCCACGGACCCAGTGCCGTCCCCGGGCCTGGCCAGCTGTTAGCGGGCGGTGTCCTCGGACCGGCCAAACCTCGGATTACCGGTCGGCGACGCTATTGCTTTGGGTACTCTCAGGACCCGTCTTGAAACACGGACCAAGGAGTCTAACATGTGCGCGAGTCATTGGGATGCATAAACCTAAAGGCGAAATGAAAGTGAAAGTCGGCCTTCGCGCCGATCGAGGGAGGATGGGCCGCGTAACAATGCGGCCCCGCACTCCCGGGGCGTCTCGTTCTCACTGCGAGAAGAGGCG

>HE

GGAATTCTGTATATAATTTTTGCCATCTGATCAGGAATAGTGGGTTCATCTTTTAGAATACTTATCCGAATAGAACTTAATAGACCTGGATCCTGAATTAGTAATGATCAAATTTACAATTCTATTGTAACTAGACATGCATTTCTAATAATTTTCTTTATAGTTATACCTTTTATAATTGGTGGTTTTGGAAACTTTCTAATTCCATTGATACTAGGTTCACCTGATATAGCGTTTCCTCGAATAAACAACGTTAGATTTTGACTGTTACCTCCATCAATTATGCTGCTGATAACAAACAATTTATTTTTTCCGAGATCGGGAACAGGATGAACCGTGTATCCTCCTCTTTCTCTTTATATATATCATTCATCTCCGTCTGTTGATTTCACTATCTTCTCTATTCACATAACTGGCATCTCTTCTATTCTAGGATCACTTAACTTCATTGTAACAATTTTTATAATAAAGAATTTCTCATTAAAGTACGATCAAATTAATCTTTTCTCATGGTCTATTTCAATTACAGTAATTTTGCTAATTCTTTCATTACCGGTACTAGCAGGAGCAATTACTATGCTACTTTTTGATCGAAATTTTAACACTTCTTTCTTTGATCCAATAGGGGGAGGAGATCCAATTCTTTATCAGCATTTATTCATCTGCCCAATGAAAAT-----TTTTAATGGCTGCAGTATAACTGACTGTACAAAGGTAGCATAATCAATTGGTTTTTAAATGAAATCTGGAATGAAAGGATTAATGAAATATGTACTGTCTCATGTGTATAATTATGAATTTAAAATTTTAATAAAAATGTTAAAATTTACTTATGGGACGATAAGACCCTATAGAATTTTATATTGAAACTTCTCAGTAGTTAATACAGAAAAAGTCTCAATATTTGGTTGGGAGGACCATTAAATTTAATTAACTT--TAATTTAGATTAACTTTGATTTAAGA-ATAAATAATGATCTTCAACTTGAAATTAATAGAATAAATTACCTTAGGGATAACAGCGTAATACTTTTCTATAGGCCACATAGAAAAAAGTGATTGCGACCTCGATGTTGAATTAGGATAAATTTTAAACGCAGGAGTTTAATAATTAAGTCTGTCGTCAACGGCGTGGGCTACCGTTGGTGCGCGATGCTCCGGATGGACCCCC-GCGGTTCCATTAGCGAGGGCACGCCACCTTCGGATCGAACGCTCCTGCGTCGTAGTCGTGCACTTCTCCCCTAGTAGAACGTCGCGACCCGTTGTGTGTCGGTCTACGGCCCGAGCGGGAGACTGTCGCGTCGCTTCGGCGCACGCGGCAGACCCTCGGTCGCCCGGCCGGCTGCACGACGGTACACTCACGGTATCGGGCCGCAGCCAATCCATT--CTCGAATGTGTGTGCGTCCATCCCGCCGCAAGCTCGGTCAGTTCTTACCCGGAGGACACGGACCCAGTGCCGTCCCCGGGCCTGGCCAGCTGTTAGCGGGCGGTGTCCTCGGACCGGCCAAACTTCGGATTACCGGTCAGCGACGCTATTGCTTTGGGTACTCTCAGGACCCGTCTTGAAACACGGACCAAGGAGTCTAACATGTGCGCGAGTCATTGGGATGCATAAACCTAAAGGCGAAATGAAAGTGAAAGTCGGCCTTCGCGCCGATCGAGGGAGGATGGGCCGCGTAACAATGCGGCCCCGCACTCCCGGGGCGTCTCGTTCTCA-TGCGAGAAGAGGCG

>GT

GGTATTTTATATATAATTTTTGCATTATGATCAGGAATTGTAGGATCATCATTTAGAATGTTAATTCGAATAGAACTTAATAATCCAGGATCTTGAATTAATAATGATCAAATTTACAATTCTATTGTAACAAGTCATGCATTTTTAATAATTTTTTTTATAGTTATACCTTTTATAATTGGTGGATTTGGAAATTTTTTAATTCCTTTAATACTAGGTTCTCCAGATATAGCTTTTCCTCGTATAAATAACGTTAGATTTTGACTTTTACCTCCTGCAATTTTAATACTTATAATAAATAATTTTTTTTTTCCAAATTCAGGAACTGGTTGAACTGTATATCCTCCTCTTTCACTATACATTTATCATTCTTCCCCTTCTGTAGATTTTACTATCTTTTCTATTCATATAACTGGTATTTCATCAATTTTAGGTTCATTAAATTTTATTGTAACAATTTTTATAATAAAAAATTTTTCAATAAAATATGATCAAATTAATCTTTTTTCTTGGTCTATTACAATTACTGTAGTTTTATTAATTCTATCATTACCTGTACTAGCTGGTGCAATTACAATATTATTGTTCGATCGAAATTTTAATACATCTTTTTTTGATCCAATAGGAGGAGGTGATCCAATTTTATATCAACACTTATTTACCTGCTCAATGAAATT-----TTTAAATGGCTGCAGTATAACTGACTGTACAAAGGTAGCATAATCAATTGGTTTTTAAATGAAATCTGGAATGAAAGGATTAATGAAATATGTACTGTCTCATATATATTT-AATGAAATTAAAATTTTAATAAAAATGTTAAAATTTATTTATGGGACGATAAGACCCTATAGAATTTTATATTAATTTATTTTATTAGTTAATATTGAATTTAAATTAATATTTAGTTGGGAGGATTATTGAATTTAATTAACTT--CAATTACTATAAACTTTAATTTAAGA-ATAATTAATGATCTTTAATTTAAAATCATTAGAATAAATTACCTTAGGGATAACAGCGTAATATTTTTTTATAGACCATATAGAAAAAAGTGATTGCGACCTCGATGTTGAATTAAGATAAATTTTAAATGCAGGAGTTTAATAATTAAGTCTGTCGTCGACGGCGTAGGCTACCGTTGGTGCGCGATGCTCCGGATGGGCCCCCCGCGGTTCCATTAGCGAGGGCACGCCACCCTCGGATCGAACGTTCCTGCGTCGTGGTCGTGCACTTCTCCCCTAGTAGGACGTCGCGACCCGTTGTGTGTCGGTCTACGGCCCGAGCGGGAGACTGTCGCGTCGCTTCGGCGCACGCGGCAGACCCTCGGTCGCCCGGCCGGCTGCACGACGGTACACTCACGGTATCGGGCCGCAGCCAATCCATT--CTCGAATGTGTGTGCGTCCATCCCGCCGCAAGCTCGGTCAGTTCTTACCCGGAGGCCACGGACCCAGTGCCGTCCCCGGGCCTGGCCAGCTGTTAGCGGGCGGTGTCCTCGGACCGGCCAAACCACGGATTACCGGTCGGCGACGCTATTGCTTTGGGTACTCTCAGGACCCGTCTTGAAACACGGACCAAGGAGTCTAACATGTGCGCGAGTCATTGGGATGCATAAACCTAAAGGCGAAATGAAAGTGAAAGTCGGCCCTCGCGCCGATCGAGGGAGGATGGGCCGCGTGACAATGCGGCCCCGCACTCCCGGGGCGTCTCGTTCTCACTGCGAGAAGAGGCG

>HF

GGAATTCTATATATAATTTTTGCTATTTGATCAGGATTAATTGGATCATCCTTTAGAATAATAATTCGAATAGAGCTTAATAGACCAGGAATATGAATTAATAATGATCAAATCTATAATTCAATTGTTACAAGACATGCATTTCTTATAATTTTTTTTATGGTTATACCTTTCATAATTGGAGGATTTGGAAATTTTTTAATTCCTTTAATATTAGGATCTCCCGATATAGCATTTCCTCGTATAAATAATGTTAGATTTTGATTATTACCACCTGCACTCATAATACTACTAATTAGTATAATATTCTATCCAAGTTCAGGTACTGGATGAACTGTTTATCCTCCACTTTCATTATATATATATCATTCATCTCCATCAGTTGATTTTACTATTTTTTCAATTCATATAACAGGAATTTCATCAATTCTTGGATCATTAAATTTTATCGTAACTATTATACTAATAAAGAATTTTTCATTAAATTTTGATCAAATTAACCTTTTTTCTTGATCAATTTCTATTACTGTAATTTTGTTAATTCTATCTCTTCCTGTATTAGCAGGAGCAATTACAATACTTCTATTTGATCGAAACTTTAATACATCATTTTTTGATCCAATAGGAGGAGGAGATCCAATTCTCTACCAGCATTTATTCATCTGCCCAATGAAATA-----TTTGAATGGCTGCAGTATAACTGACTGTACAAAGGTAGCATAATCAATTGATTTTTAAATGAAATCTGGAATGAAAGGATTAATGAAATATAAACTGTCTCACATATATTT-TATGAATTTAAAATTTTAATAAAAATGTTAAAATTTTTTTATGGGACGATAAGACCCTATAGAATTTTATACTAATTTATTTTAATACTTAATATAAAATTTAAATTAGTATTTGATTGGGAGGATTATTAAATTTATAAAACTTTTTAAATTATGTAAACTTTAATTTAAGA-AATTATAATGATCTTTAATTTAAAATATTTAGAATAAATTACCTTAGGGATAACAGCGTAATACTTTTTTATAGACCACATAGAAAAAAGTGATTGCGACCTCGATGTTGAATTAAGATAAATTTTAAACGCAGGAGTTTAATAATTAAGTCTGTCGTCGACGGCGTTGGCTACCGTTGGTGCGCGATGCTCCGGATGGGCCCCT-GCGGTTCCATTAGCGAGGGCACACCACCTTCGGATCGAACGTTCCTGCGTCGTAGTCGTGCACTTCTCCCCTAGTAGGACGTCGCGACCCGTTGTGTGTCGGTCTACGGCCCGAGCGGGAGACTGTCGCGTCGTTTCGGCGCACGCTGCAGACCCTCGGTCGCCCGGCCGGCTGCACGACGGTACACTAACGGTATCGGGCCGCAGCCAATCCATT--CTCGAATGTGTGTGCGTCCATCCCGCCGCAAGCTCGGTCAGTTCTTACCCGGAGGT-ACGGACCCAGTGCCGTCCCCGGGCCTGGCCAGCTGTTAGCAGGCGGTGTCCTCGGACCGGCCAAACCACGGATTACCGGTCGGCGACGCTATTGCTTTGGGTACTCTCAGGACCCGTCTTGAAACACGGACCAAGGAGTCTAACATGTGCGCGAGTCATTGGGATGCATAAACCTAAAGGCGAAATGAAAGTGAAAGTCGGCCCTCGCGCCGATCGAGGGAGGATGGGCCGCGTAACCATGCGGCCCCGCACTCCCGGGGCGTCTCGTTCTCACTGCGAGAAGAGGCG

>LC

GGTATTTTATATATAATTTTTGCCCTGTGATCTGGAATTATTGGATCATCTTTTAGTATATTAATTCGTATAGAACTTAATAGACCTGGAATATGAATTAATAATGATCAGATCTATAATTCAATTATTACAAGTCATGCGTTTTTGATAATTTTTTTCATAGTTATGCCTTTTATAATTGGTGGATTTGGGAATTTTCTAGTTCCAATAATACTTGGATCACCTGATATAGCATTTCCTCGAATAAACAATATTAGTTTTTGATTATTACCTCCTTCATTGATATTACTTTTACTCAGAAGATTATTTTTTCCAAGATCAGGAACGGGATGAACAATCTATCCTCCATTATCTTTGTATATGTATCATTCGTCTCCTTCAGTTGATTTTACAATTTTCTCTATTCACATAACTGGAATTTCATCTATTTTAGGATCATTGAATTTCATTGTAACTATTATAATAATAAAAAATTTATCTTTAAACTACGATCAGATTAATTTATTTTCATGATCTATTTCAATTACTGTAATTCTATTAATTTTATCACTTCCTGTTTTAGCTGGAGCAATTACAATATTGTTATTTGACCGAAATTTCAACACTTCTTTCTTTGATCCCATAGGAGGAGGAGATCCTATCTTATATCAACATTTATTTATCTGCCCAATGAAATAAATAATTTAAATGGCTGCAGTATAACTAACTGTACAAAGGTAGCATAATCAATTGTTTTTTAAATGAAATCTGGAATGAAAGGATTAATGAAATATGAACTGTCTCATGCATATTTATACAAACTTAAAATTTTAATAAAAATGTTAAAATTTTTTTATGGGACGATAAGACCCTATAGAATTTTATACTAAATTATTTTAATACTTAATATAAAATTTGATTTAGTATTTGGTTGGGAGGATTATTAAATTAGAAAAACTT--TAATTTTTGAAAACTTTAATTTAAGAGAAATATTATGATCTTGAAATTTAAATTATCAGATTAAATTACCTTAGGGATAACAGCGTTATATTTTTTTATAGACCTTATAGAAAAAAGTGATTGCGACCTCGATGTTGAATTAAGATAAATTTTGAACGCAGGAGTTTAATAATTAAGTCTGTCGTCGACGGCGTTGGCTACCGTTGGTGCGCGATGCTCCGGATGGGCCCCT-GCGGTTCCATTAGCGAGGGCACACCACCTTCGGATCGAACGTTCCTGCGTCGTAGTCGTGCACTTCTCCCCTAGTAGGACGTCGCGACCCGTTGTGTGTCGGTCTACGGCCCGAGCGGGAGACTGTCGCGTCGTTTCGGCGCACGCGGCAGACCCTCGGTCGCCCGGCCGGCTGCACGACGGTACACTAACGGTATCGGGCCGCAGCCAATCCATTTTCTCGAATGTGTGTGCGTCCATCCCGCCGCAAGCTCGGTCAGTTCTTACCCGGAGGT-ACGGACCAAGTGCCGTCCCCGGGCCTGGCCAGCTGTTAGCAGGCGGTGTCCTCGGACCGGCCAAACCACGGATTACCGGTCGGCGACGCTATTGCTTTGGGTACTCTCAGGACCCGTCTTGAAACACGGACCAAGGAGTCTAACATGTGCGCGAGTCATTGGGATGCATAAACCTAAAGGCGAAATGAAAGTGAAAGTCGGCCTTCGCGCCGATCGAGGGAGGATGGGCCGCGTAACCATGCGGCCCCGCACTCCCGGGGCGTCTCGTTCTCACTGCGAGAAGAGGCG

>TC

GGAGTTTTATACATAATCTTTGCTTTATGGTCTGGAATTGTTGGTTCCTCGTTTAGAATACTTATTCGCATGGAGTTAAATAGTCCTGGAATATGAATTAATAATGATCAGATTTATAACTCAATTGTTACAAGTCATGCATTTCTAATAATTTTTTTTATAGTTATACCATTTATAATTGGAGGTTTTGGAAACTTTTTGGTTCCTCTGATGCTTGGAGCTCCTGATATGGCTTTTCCTCGAATAAATAATGTAAGATTTTGACTTTTACCTCCTTCTCTTATGTTATTGCTACTTAGAAATTTTATTTTTCCGAGGTCAGGTACAGGGTGAACTGTTTACCCTCCTCTTTCTTCTTTAATGTACCATCCTTCTCCTTCTGTAGATTTTACTATTTTTTCAATTCATATGACTGGGGTTTCATCTATTTTAGGGTCACTAAATTTTATTGTAACAATTTTTATAATAAAAAATTTTTCAATTAACTATGATCAAATTACACTATTTTCGTGATCTATTTCAATTACTGTAATTCTTTTAATCATCTCTCTTCCTGTTTTAGCTGGAGCTATTACAATACTTTTATTTGATCGAAATTTTAATACTTCATTTTTTGATCCAGTGGGAGGGGGTGATCCAATTCTTTACCAACATCTATTTACCTGCCCAATGAA-TA-----CTTGAATGGCTGCAGTATAACTGACTGTACAAAGGTAGCATAATCAATTGGTTTTTAAATGAAATCTGGAATGAAAGGATTAATGAAATATATACTGTCTCATGTATATAA-AATGAACTTAAAATTTTAATTAAAATGTTAAAATTTTTTTATGGGACGATAAGACCCTATAGAATTTTATATTGCTGCATTTTAATACTTAATATAAAATATGTAGTAATATTTGGTTGGGAGGACTATTAAATTTGACTAACTTT-AATTTTTTGATTACTTTGATTAAAGA-AAATACAATGATCTTTAAATTAAAATATCTAGATTAAATTACCTTAGGGATAACAGCGTAATATTTTTTTATAGATCGTATAGAAAAAAATGATTGCGACCTCGATGTTGAATTAAGATAAATTTTAAACGCAGGAGTTTAATGATTAAGTCTGTCGTCGACGGCGTTGGCTACCGTTTGTGCGCGATGCTCCGGGTGGGACCCT-GCGGTTCCATTAGCGAGGGCACGCCACCTTCGGATCGAACTTTCCTGCGTCGTAGTCGTGCACTTCTCCCCTAGTAGGACGTCGCGACCCGTTGTGTGTCGGTCTACGGCCCGAGCGGGAGACTGTCGCGTCGCTTCGGCGCACGCGGCAGACCCTCGGTCGCCCGGCCGGCTGCACGACGGTACACCAACGGTATCGGGCCGCAGCCAATCCATT--CTCGAATGTGTGTGCGTCCATCCCGCCGCAAGCTCGGTCAGTTCTTACCCGGAGGT-ACGGACCCTGTGCCGTCCCCGGGCCTGGCCAGCTGTTAGCAGGCGGTGTCCTCGGACCGGCCAAACCTCGGATTACCGGTCGGCGACGCTATTGCTTTGGGTACTCTCAGGACCCGTCTTGAAACACGGACCAAGGAGTCTAACATGTGCGCGAGTCATTGGGATGCACAAACCTAAAGGCGAAATGAAAGTGAAAGTCGGCCTTCGCGCCGATCGAGGGAGGATGGGCCGCGTAACCATGCGGCCCCGCACTCCCGGGGCGTCTCGTTCTCACTGCGAGAAGAGGCG

>TM

GGAGTTTTATATATAATTTTCGCATTATGATCAGGAATTATTGGATCATCTCTTAGTATATTAATTCGAATAGAACTTAATAATCCAGGTATATGAATCAATAATGATCAAATTTATAATTCAGTTGTTACAAGACATGCATTTATTATAATTTTTTTTATAGTTATACCATTTATAATTGGAGGATTTGGAAATTTTTTAGTTCCTCTAATACTAGGTTCACCAGATATAGCATTTCCCCGAATAAATAATATTAGGTTTTGATTATTACCTCCTGCATTAATACTTTTACTAACTAGAATAATATTTTATCCAAGATCAGGTACTGGATGAACTGTATATCCTCCTCTTTCACTATATATATATCATTCATCTCCATCAGTTGATTTTACTATTTTTTCAATTCACATAACAGGAATTTCATCAATTCTTGGATCATTAAATTTTATTGTTACTATTATAATAATAAAAAATTTCTCATTGAACTATGATCAAATTAATTTGTTTTCATGATCAATTTCTATTACTGTAATTTTATTAATTTTATCGCTTCCTGTATTAGCAGGTGCTATTACAATACTACTCTTTGATCGAAACTTTAACACTTCATTTTTTGATCCAATAGGAGGAGGAGATCCAATTCTCTATCAACATTTATTCATCTGCCCAATGAAATA-----TTTGAATGGCTGCAGTATAACTGACTGTACAAAGGTAGCATAATCAATTGGTTTTTAAATGAAATCTGGAATGAAAGGATTAATGAAATATAAACTGTCTCACGTATATTT-TATGAAATTAAAATTTTAATAAAAATGTTAAAATTTTTTTATGGGACGATAAGACCCTATAGAATTTTATACTAATTTATTTTAATACTTAATATAAAATTTAAATTAGTATTTGATTGGGAGGATTATTAAATTTAAAGAACTT--TAATTTTTGTAAACTTCAATTCAAGA-ATACGTAATGATCTTCAATTTGAAATCTCTAGAATAAATTACCTTAGGGATAACAGCGTAATATTTTTTTATAGACCATATAGAAAAAAATGATTGCGACCTCGATGTTGAATTAAGATAAATTTTAAACGCAGGAGTTTAATAATTAAGTCTGTCGTCGACGGCGTTGGCTACCGTTGGTGCGCGATGCTCCGGATGGGCCCCT-GCGGTTCCATTAGCGAGGGCACACCACCTTCGGATCGAACGTTCCTGCGTCGTAGTCGTGCACTTCTCCCCTAGTAGGACGTCGCGACCCGTTGTGTGTCGGTCTACGGCCCGAGCGGGAGACTGTCGCGTCGTTTCGGCGCACGCTGCAGACCCTCGGTCGCCCGGCCGGCTGCACGACGGTACACTAACGGTATCGGGCCGCAGCCAATCCATT--CTCGAATGTGTGTGCGTCCATCCCGCCGCAAGCTCGGTCAGTTCTTACCCGGAGGT-ACGGACCCAGTGCCGTCCCCGGGCCTGGCCAGCTGTTAGCAGGCGGTGTCCTCGGACCGGCCAAACCACGGATTACCGGTCGGCGACGCTATTGCTTTGGGTACTCTCAGGACCCGTCTTGAAACACGGACCAAGGAGTCTAACATGTGCGCGAGTCATTGGGATGCATAAACCTAAAGGCGAAATGAAAGTGAAAGTCGGCCTTCGCGCCGATCGAGGGAGGATGGGCCGCGTAACCATGCGGCCCCGCACTCCCGGGGCGTCTCGTTCTCACTGCGAGAAGAGGCG
