# Supplementary material for: Genetic structure of the commercial stingless bee Heterotrigona itama (Apidae: Meliponini) in Thailand
Source: PLoS One. 2024 Dec 4;19(12):e0312386. doi: 10.1371/journal.pone.0312386 (PMC11616864; doi:10.1371/journal.pone.0312386)
Supplement: S2 File — (DOCX) [file pone.0312386.s003.docx]

#NEXUS

BEGIN TAXA;

DIMENSIONS NTAX=42;

TAXLABELS

Hap_1

Hap_2

Hap_3

Hap_4

Hap_5

Hap_6

Hap_7

Hap_8

Hap_9

Hap_10

Hap_11

Hap_12

Hap_13

Hap_14

Hap_15

Hap_16

Hap_17

Hap_18

Hap_19

Hap_20

Hap_21

Hap_22

Hap_23

Hap_24

Hap_25

Hap_26

Hap_27

Hap_28

Hap_29

Hap_30

Hap_31

Hap_32

Hap_33

Hap_34

Hap_35

Hap_36

Hap_37

Hap_38

Hap_39

Hap_40

Hap_41

Hap_42

;

END;

BEGIN CHARACTERS;

DIMENSIONS NCHAR=660;

FORMAT DATATYPE=DNA MISSING=? GAP=- MATCHCHAR=.;

MATRIX

Hap_1 GGAATTCTATATATAATTTTTGCTATCTGATCAGGTATTGTTGGTTCTTCCTTCAGAATACTTATTCGAATGGAACTTAATAGCCCTGGAACTTGAATTAGTAATGATCAGATCTACAACTCTATTGTGACTAGGCATGCATTTCTAATAATTTTTTTTATAGTTATACCTTTTATAATCGGGGGTTTTGGAAATTTTTTAATCCCGTTAATACTAGGTTCTCCTGATATAGCTTTCCCTCGAATAAACAATATTAGCTTCTGACTACTACCTCCTTCACTTATACTGCTAATAACAAATAATCTATTTTTTCCGAATTCAGGAACTGGATGAACTGTATACCCTCCTCTTTCTCTCTACATGTATCATCCATCTCCGTCAGTGGACTTCACTATTTTTTCAATTCATATGACAGGAATTTCATCAATTCTGGGGTCACTTAACTTTATTGTGACAATTTTTATAATAAAGAACTTTTCTTTAAAGTACGATCAAATTAATCTTTTTTCATGATCTATCTCAATTACTGTAATTTTACTAATTCTTTCTCTTCCAGTTCTAGCAGGAGCAATTACTATGCTGTTATTTGATCGAAATTTTAATACTTCCTTCTTTGATCCAATAGGAGGGGGAGACCCGATTCTTTATCAACATTTATTT

Hap_2 ........T..............................................................................................................................................................................................................................A..................................................................................................................................................................................................................................................................................................G.........................................................................................................................................

Hap_3 ........T.........................................................................C.............................................................................A......................................................................A.........A..............A................................................................T....................................................................................................................................................A.....................................................................T..............T........................................................................................................

Hap_4 ..............................................................................................................A.....................................................................................................................................................................................................................................................................................................................................................................................................................................................................................................................................................................

Hap_5 ..G.....G...............................C.......................................................................................A.................G.................A..............T..A..G..............G..T..AC..........A.................T....A..................T..........................................T....A.....A.......................G..T....................A..C...........T.....A.....T..............C.....A....................T..A.................AG....C..............T................................................G...................C........C...T..........G..............A........C......................................A.....T..A...............C.....

Hap_6 ........G...........................................................................................................................................................A.................A....................T...C.......................A.......C.....G........C................................................T....C...................................C.................A......T...................T....................A..G.................T....................A.............................G........C..............................G.........................................................................................................................................

Hap_7 ..G.....G...............................C.......................................................................................A.................G.................A..............T..A..G..............G..T...C..........A.........................................T..........................................T....A.....A...............................................A..C...T....C........A.....G..............C.....A...........G........T..A.................A....................T...........................................................C........C........C......................................C...........C..C.............................T..A...............C.....

Hap_8 ..G.....G...............................C.......................................................................................A.................G.................A..............T..A..G..............G..T...C..........A.........................................T..........................................T..........A.......................T.......................A..C...T....C........A.....T..............C.....A....................T..A.................A....................T...........................................................C........C........C..............GA......................C.....C.....C................................T..A...............C.....

Hap_9 ..G.....G...............................C.......................................................................................A.................G.................A..............T..A..G..............G..T...C..........A............A.........A..................T....................................T.....T..........A.......................T.......................A..C...T....C........AA....T..............C.....A....................T..A.................A....................T...........................................................C........C........C...T..................................C.....C.....C.................T..............T..A...............C.....

Hap_10 ..G.....G...............................C.......................................................................................A.................G.................A..............T..A..G..............G..T...C..........A.........................................T..........................................T..........A...............................................A..C...T....C........A.....T..............C.....A....................T..A.................A....................T...........................................................C........C........C......................................C...........C..C.............................T..A...............C.....

Hap_11 ........G...........................................................................................................................................................A.................A.......C............T...C.......................A.......C.....G........C................................................T....C...................................C.................A......T...................T....................A..G.................T....................A.............................G........C........................................................................................................................................................................

Hap_12 ..G.....G...............................C.......................................................................................A.................G.................A..............T..A..G..............G..T...C..........A.........................................T..........................................T..........A...............................................A..C...T....C........A.....T..............C.....A........C...........T..A.................A....................T....................................................................C........C......................................C...........C................................T..A...............C.....

Hap_13 ..G.....G...............................C.......................................................................................A.................G.................A..............T..A..G..............G..T...C..........A.........................................T..........................................T..........A...............................................A..C...T....C........A.....T..............C.....A....................T..A.................A....................T...........................................................C........C........C..............GA......................C...........C................................T..A...............C.....

Hap_14 ..G.....G...............................C...........................................................................................................................A.................A....................T...C.......................A.......C.A...G........C.A..............................................T....C...................................C.................A......T...................T....................A..G.................T....................A.............................G........C........................................................................................................................................................................

Hap_15 ........G...........................................................................................................................................................A.................A....................T...C...............................C.....G........C................................................T....C...................................C.................A......T...................T....................A..G.................T....................A.............................G........C.............................G..........................................................................................................................................

Hap_16 ........G.............................................................................................................................T.............................A.................A..C...................................C...............................................................................................C......................................T.....A...........C..............T..............C.....A....................T....................A...............................................................................................................................................................................................................

Hap_17 ..G.....G...............................C.......................................................................................A.................G.................A..............T..A..G..............G..T..AC..........A.................T.......................T.....T........C...........T...............T..........A.......................G.......................A..C...T....C..T.....A.....T..............C.....A....................T..A.................A....................T....................................................................C........C...........G..........................C...........C..C................................A...........G...C.....

Hap_18 ........G.............................................................................................................................T.............................A.................A..C...................................C...............................................................................................C......................................T.....A..........................T....................A....................T....................A...............................................................................................................................................................................................................

Hap_19 ........G.............................................................................................................................T.............................A.................A..C...................................C.........A........................A............................................................C......................................T.....A..........................T....................A....................T....................A...............................................................................................................................................................................................................

Hap_20 ........G.............................................................................................................................T.............................A.................A..C...................................C.........A.........A..............A............................................................C......................................T.....A..........................T....................A....................T....................A...............................................................................................................................................................................................................

Hap_21 ........G.............................................................................................................................T.............................A.................A..C...................................C.........A.....................................................................................C......................................T.....A..........................T....................A....................T....................A...............................................................................................................................................................................................................

Hap_22 ........G.............................................................................................................................T.............................A.................A..C...................................C.........A.........A..............A...................................................................................................T.....A..........................T....................A....................T....................A...............................................................................................................................................................................................................

Hap_23 ........G.....................................................................................................A.......................T............................AA.................A..C...................................C.........A.........A..............A............................................................C......................................T.....A..........................T....................A....................T....................A...............................................................................................................................................................................................................

Hap_24 ........G.....................................................................................................A.......................T.............................A.................A..C...................................C.........A.........A..............A............................................................G......................................T.....A..........................T....................A....................T....................A.....................................................................G.........................................................................................................................................

Hap_25 ....................................................................................................................................................................A......................................T...C.....................................G.........................................................T....C.............................G.....C.................A......T...................T....................A....................T....................A.............................G.........................................C....................................................................................................................C..............G...

Hap_26 ........G.............................................................................................................................T.............................A.................A..C...................................C...............................................................................................C......................................T.....A...........C..............T....................A....................T....................A...............................................................................................................................................................................................................

Hap_27 ........G.....................................................................................................A.......................T.........................A..AA.................A..C...................................C.........A.........A..............A............................................................C......................................T.....A..........................T....................A....................T....................A...............................................................................................................................................................................................................

Hap_28 ..G.....T....................G.....C....C.........A..T...........C.....A..G........A.....G..............C.......................A........C.................C........A..G...........T..A..G........C...C....T..AA..........A....................C.................G..T......T...................T...............T..........C.................G.....G..............C..T.....A..C...T....C..T..T..A.....T.........................................T....................A....................T....................G..........................G........G............A.......C...........T.........................................A..A..T........G........A.....T........................

Hap_29 ..G.....T....................G.....C....C.........A..T...........C.....A..G........A.....G..............C.......................A........C.................C........A..G...........T..A..G........C...C....T..AA..........A....................C.................G..T......T...................T...............T..........C.................G.....G..............C..T.....A..C...T.......T..T..A.....T.........................................T....................A....................T....................G..........................G........G............A.......C...........T.........................................A..A..T........G........A.....T........................

Hap_30 ..G.....G...............................C.......................................................................................A.................G.................A..............T..A..G..............G..T..AC..........A.................T.......................T.....T....................................T..........A.......................G.......................A..C...T....C..T.....A.....T..............C.....A....................T..A.................A....................T....................................................................C........C...........G..........................C...........C................................T..A...............C.....

Hap_31 ..G.....G...............................C.......................................................................................A.................G........C........A..............T..A..G..............G..T..AC..........A.................T.......................T.....T....................................T..........A.......................G.......................A..C...T....C..T.....A.....T..C...........C.....A.....G..............T..A.................A....................T......C.............................................................C........C...........G..........................C...........C..C.............................T..A...............C.....

Hap_32 ..G.....G...............................C.......................................................................................A.................G.................A...........G..T..A..G..............G..T..AC..........A.................T.......................T.....T....................................T..........A.......................G.......................A..C...T....C..T.....A.....T..............C.....A.....G..............T..A.................A....................T.........................................................................................G..........................C...........C..C.............................T..A...............C.....

Hap_33 ..G.....T....................G.....C....C.........A..T.................A...........A.....G................................C.....A..........................C........A..G...........T..A..G........C...C....T..AA..........A....................C.................G..T......T....C..C...........................T..........C.................G.....G..............C..T.....A..C...T.......T..T..A.....T.........................................T....................A....................T....................G..........................G........G............A.......C.....................................................A..A..T........G........A.....T........................

Hap_34 ..G.....G...............................C.......................................................................................A.................G.................A..............T..A..G..............G..T..AC..........A............A....T.......................T.....T....................................T..........A.......................G.......................A..C...T....C..T.....A.....T..............C.....A....................T..A.................A....................T....................................................................C........C...........G..........................C...........C................................T..A...............C.....

Hap_35 ........G...........................................................................................................................................................A.................A....................T...C...............................C.....G........C...............................................CT....C...................................C.................A......T...................T....................A....................T....................A.............................G........C........................................................................................................................................................................

Hap_36 ..G.....T....................G.....C....C.........A..T.................A...........A.....G................................C.....A..........................C........A..G...........T..A..G........C...C....T..AA..........A....................C.................G..T......T....C..C...........................T..........C.................G.....G..............C..T.....A..C...T.......T..T..A.....T................................G........T....................A....................T....................G..........................G........G............A.......C.....................................................A..A..T........G........A.....T........................

Hap_37 ..G.....G...............................C.......................................................................................A.................G.................A...........G..T..A..G..............G..T..AC..........A.................T.......................T.....T...................................CT..........A.......................G.......................A..C...T....C..T.....A.....T..............C.....A.....G..............T..A.................A....................T.........................................................................................G..........................C...........C..C.............................T..A...............C.....

Hap_38 ........G...........................................................................................................................................................A.................A....................T...C...............................C.....G........C................................................T....C...................................C.................A......T...................T....................A..G.................T....................A.............................G........C........................................................................................................................................................................

Hap_39 ..G.....T....................G.....C....C.........A..T.................A...........A............................................A..........................C........A..G...........T..A..G........C...C....T..AA..........A....................C.................G..T......T....C..C...........................T..........C.................G.....G..............C..T.....A..C...T.......T..T..A.....T.........................................T....................A....................T....................G..........................G........G............A.......C.....................................................A..A..T........G........A.....T........................

Hap_40 ..G.....C....................G.....C....C.........A..T.................A...........A.....G......................................A..........................C........A..G...........T..A..G........C...C....T..AA..........A....................C.................A..T...........C..............................T..........C.................G........T...........C..T.....A..C...T.......T..T..A.....T.........................................C....................A....................T....................G..........................T........G............A.......C.....................................................A..A..T........G........A.....T........................

Hap_41 ..G.....C....................G.....C....C.........A..T.................A...........A.....G......................................A..........................C........A..G...........T..A..G........C...C....T..AA..........A....................C.................A..T...........C..............................T..........C.................G........T...........C..T.....A..C...T.......T..T..AA....T.........................................C....................A....................T....................G..........................T........G............A.......C.....................................................A..A..T........G........A.....T........................

Hap_42 ..G.....T....................G.....C....C.........A..T...........C.....A..G........A.....G..............C.......................A........C.................C........A..G...........T..A..G........C...C....T..AA..........A....................C.................G..T......T...................T...............T..........C.................G.....G..............C..T.....A..C...T.......T..T..A.....T.........................................T....................A....................T....................G..........................G........G............A.......C...........T.........................................A..A..T........G........A.....T.......................C

;

END;

BEGIN TRAITS;

Dimensions NTRAITS=3;

Format labels=yes missing=? separator=Comma;

TraitLabels Krabi Nakhon Nara;

Matrix

Hap_1 2, 0, 0

Hap_2 1, 0, 0

Hap_3 1, 0, 0

Hap_4 1, 0, 0

Hap_5 1, 0, 0

Hap_6 2, 0, 0

Hap_7 2, 0, 0

Hap_8 1, 0, 0

Hap_9 1, 0, 0

Hap_10 1, 0, 0

Hap_11 0, 1, 0

Hap_12 0, 1, 0

Hap_13 0, 1, 0

Hap_14 0, 1, 0

Hap_15 0, 1, 0

Hap_16 0, 1, 0

Hap_17 0, 1, 0

Hap_18 0, 4, 0

Hap_19 0, 1, 0

Hap_20 0, 2, 0

Hap_21 0, 1, 0

Hap_22 0, 1, 0

Hap_23 0, 1, 0

Hap_24 0, 1, 0

Hap_25 0, 2, 0

Hap_26 0, 1, 0

Hap_27 0, 1, 0

Hap_28 0, 0, 3

Hap_29 0, 0, 4

Hap_30 0, 0, 3

Hap_31 0, 0, 2

Hap_32 0, 0, 1

Hap_33 0, 0, 1

Hap_34 0, 0, 1

Hap_35 0, 0, 1

Hap_36 0, 0, 1

Hap_37 0, 0, 1

Hap_38 0, 0, 1

Hap_39 0, 0, 3

Hap_40 0, 0, 11

Hap_41 0, 0, 1

Hap_42 0, 0, 1

;

END;
