# Supplementary material for: Genetic structure of the commercial stingless bee Heterotrigona itama (Apidae: Meliponini) in Thailand
Source: PLoS One. 2024 Dec 4;19(12):e0312386. doi: 10.1371/journal.pone.0312386 (PMC11616864; doi:10.1371/journal.pone.0312386)
Supplement: S3 File — (DOCX) [file pone.0312386.s004.docx]

#NEXUS

BEGIN TAXA;

DIMENSIONS NTAX=10;

TAXLABELS

Hap_1

Hap_2

Hap_3

Hap_4

Hap_5

Hap_6

Hap_7

Hap_8

Hap_9

Hap_10

;

END;

BEGIN CHARACTERS;

DIMENSIONS NCHAR=440;

FORMAT DATATYPE=DNA MISSING=? GAP=- MATCHCHAR=.;

MATRIX

Hap_1 ATCTGCCCAATGAATTTTTTTAATGGCTGCAGTATAACTGACTGTACAAAGGTAGCATCAATTGGTTTTATGAAATCTGGAATGAAAGGATTAATGAAATATGTACTGTCTCAATTGTACAATTATGAAATTAAAATTTTAATAAAAATGTTAAAATTCACTTATGGGACGATAAGACCCTATAGAATTTTATATTGAAATTACTCAGTAGTTAATTCAGAAATAGTTTCAATATTTGATTGGGAGGATTATACTCCAACTTTAATTGTTAACTTTAATTTAAGAGTAGATAATGATCTTCAATTTGAAATTGCTAGAATAAATTACCTTAGGGATAACAGCGTAATACTTTTTTATAGGCCATATAGAAAAAAGTGGTTGCGACCTCGATGTTGAATTAGGATAAATTTTAAATGCAGGAGTTTAATAATTAAGTCTGT

Hap_2 ...........................................................................................................................................................................................................................................................................................................................................T...................................A....................T...........T.........................T.......A.....

Hap_3 .............G...............................................................................................................................................................................................................................................A..........................................................................................................................................................................................

Hap_4 ................C.......................................................................................................................................................................................................................................................................................................................................................................................................................................

Hap_5 .............................................................................................................................................................................................................................................................A..........................................................................................................................................................................................

Hap_6 ....................................................................................................................A........................................................................................................................................A..........................................................................................................................................................................................

Hap_7 ................C.............................................................................................................................................T.............................................................................................................................G...........................................................................................................................................................

Hap_8 ......................TA.........................................................................................................................................................................................................A...........................T.T................................A.......................................................................T...............................................................................

Hap_9 .................................................................................................................................................................................................................................A...........................T.T................................A.......................................................................T...............................................................................

Hap_10 ..........................................................................................................................C......................................................................................................A...........................T.T................................A.......................................................................T...............................................................................

;

END;

BEGIN TRAITS;

Dimensions NTRAITS=3;

Format labels=yes missing=? separator=Comma;

TraitLabels Krabi Nakhon Nara;

Matrix

Hap_1 4, 14, 0

Hap_2 1, 0, 0

Hap_3 1, 0, 0

Hap_4 2, 2, 2

Hap_5 3, 3, 8

Hap_6 2, 0, 0

Hap_7 0, 2, 0

Hap_8 0, 0, 1

Hap_9 0, 0, 7

Hap_10 0, 0, 17

;

END;
