# Supplementary material for: Genetic structure of the commercial stingless bee Heterotrigona itama (Apidae: Meliponini) in Thailand
Source: PLoS One. 2024 Dec 4;19(12):e0312386. doi: 10.1371/journal.pone.0312386 (PMC11616864; doi:10.1371/journal.pone.0312386)
Supplement: S4 File — (DOCX) [file pone.0312386.s005.docx]

#NEXUS

BEGIN TAXA;

DIMENSIONS NTAX=5;

TAXLABELS

Hap_1

Hap_2

Hap_3

Hap_4

Hap_5

;

END;

BEGIN CHARACTERS;

DIMENSIONS NCHAR=605;

FORMAT DATATYPE=DNA MISSING=? GAP=- MATCHCHAR=.;

MATRIX

Hap_1 CGTCGACGGCGTAGGCTACCGTTGGTGCGCGATGCTCCGGATGGACCCCCGCGGTTCCATCGAGGGCACGCCACCCTCGGATCGAACGCTCCTGCGTCGTCGTGCACTTCTCCCCTAGTAGAACGTCGCGACCCGTTGTGTGTCGGTCTACGGCCCGAGCGGGAGACTGTCGCGTCGCTTCGGCGCACGCGGCAGACCCTCGGTCGCCCGGCCGGCTGCACGACGGTACACTCACGGTATCGGGCCGCAGCCAATCCATTCTCGAATGTGTGTGCGTCCATCCCGCCGCAAGCTCGGTCAGTTCTTACCCGGAGGCCACGGACCCAGTGCCGTCCCCGGGCCTGGCCAGCTGTTAGCGGGCGGTGTCCTCGGACCGGCCAAACCTCGGATTACCGGTCGGCGACGCTATTGCTTTGGGTACTCTCAGGACCCGTCTTGAAACACGGACCAAGGAGTCTAACATGTGCGCGAGTCATTGGGATGCATAAACCTAAAGGCGAAATGAAAGTGAAAGTCGGCCTTCGCGCCGATCGAGGGAGGATGGGCCGCGTAACAATGCGGCCCCGCACTCCCGGGGCGTCTCGTTCTCACTGCGAGAAGAGGCG

Hap_2 .....................................................................................................................................................................G.......................................................................................................................................................................................................................................................................................................................................................................................................................................................

Hap_3 ....................................................................................................................................................................................................................................................................................................................................................................................................................................................................................................................................................AG...................................................................A...

Hap_4 ..................................................................................................................................................................................................................................................................................................................................................................................................................................................................G..........................................................................................................................A...............................

Hap_5 .....................................................................................................................................................................................................................................................................................................................................................................................................................................................................................................................................................G.......................................................................

;

END;

BEGIN TRAITS;

Dimensions NTRAITS=3;

Format labels=yes missing=? separator=Comma;

TraitLabels Krabi Nakhon Nara;

Matrix

Hap_1 9, 15, 27

Hap_2 2, 4, 7

Hap_3 1, 0, 0

Hap_4 1, 0, 0

Hap_5 0, 2, 1

;

END;
